# Development of a CNS-permeable reactivator for nerve agent exposure: an iterative, multi-disciplinary approach

**DOI:** 10.1038/s41598-021-94963-2

**Published:** 2021-07-30

**Authors:** Brian J. Bennion, Michael A. Malfatti, Nicholas A. Be, Heather A. Enright, Saphon Hok, C. Linn Cadieux, Timothy S. Carpenter, Victoria Lao, Edward A. Kuhn, M. Windy McNerney, Felice C. Lightstone, Tuan H. Nguyen, Carlos A. Valdez

**Affiliations:** 1 Biosciences and Biotechnology Division, Lawrence Livermore National Laboratory, Livermore, CA 94550 USA; 2 Nuclear and Chemical Sciences Division, Lawrence Livermore National Laboratory, Livermore, CA 94550 USA; 3 Forensic Science Center, Lawrence Livermore National Laboratory, Livermore, CA 94550 USA; 4 United States Army Medical Research Institute of Chemical Defense, Aberdeen, MD 21010 USA; 5 Global Security Directorate, Lawrence Livermore National Laboratory, Livermore, CA 94550 USA; 6 Affiliation: Mental Illness Research, Education and Clinical Center, Veterans Affairs, Palo Alto, CA 94304 USA; 7 Affiliation: Department of Psychiatry, Stanford University School of Medicine, Stanford, CA 94305 USA

**Keywords:** Computational biology and bioinformatics, Drug discovery, Chemistry

## Abstract

Nerve agents have experienced a resurgence in recent times with their use against civilian targets during the attacks in Syria (2012), the poisoning of Sergei and Yulia Skripal in the United Kingdom (2018) and Alexei Navalny in Russia (2020), strongly renewing the importance of antidote development against these lethal substances. The current standard treatment against their effects relies on the use of small molecule-based oximes that can efficiently restore acetylcholinesterase (AChE) activity. Despite their efficacy in reactivating AChE, the action of drugs like 2-pralidoxime (2-PAM) is primarily limited to the peripheral nervous system (PNS) and, thus, provides no significant protection to the central nervous system (CNS). This lack of action in the CNS stems from their ionic nature that, on one end makes them very powerful reactivators and on the other renders them ineffective at crossing the Blood Brain Barrier (BBB) to reach the CNS. In this report, we describe the use of an iterative approach composed of parallel chemical and in silico syntheses, computational modeling, and a battery of detailed in vitro and in vivo assays that resulted in the identification of a promising, novel CNS-permeable oxime reactivator. Additional experiments to determine acute and chronic toxicity are ongoing.

## Introduction

Organophosphorus-based nerve agents (OPNAs) as a class are arguably the most toxic chemicals ever manufactured due to their lethality and ease of production^1^. With members like sarin, soman, and VX, this class of compounds possesses a high potential to cause mass casualties^2,3^. The re-emergence of nerve agents in recent assassinations and trans-national conflicts has resulted in a renewed notoriety in the public eye^4^. Exposure to OPNAs results in the rapid inactivation of the enzyme acetylcholinesterase (AChE) in both the peripheral and central nervous systems (PNS, CNS) by forming a covalently adducted AChE, which can result in seizures, convulsions, respiratory distress and ultimately death^5,6^. Due to the swift toxicity observed after OPNA exposure, a fast acting, efficient therapeutic is required to counter these effects rapidly after exposure. Currently, several antidotes exist that can reactivate the adducted AChE back into its functional form. One antidote is 2-pralidoxime (2-PAM), a quaternary pyridinium oxime, that was developed over 60 years ago and is approved for use by the United States Food and Drug Administration (FDA)^7,8^. However, 2-PAM’s positively charged nitrogen center prevents it from crossing the blood–brain barrier (BBB), essentially negating CNS efficacy. In contrast, OPNAs readily cross the BBB due to their hydrophobic make-up, resulting in impaired CNS function^9^. The current challenge lies in developing a neutral, more hydrophobic small molecule that crosses the BBB and reactivates inhibited AChE for both CNS and PNS efficacy, thus significantly improving the standard of care for exposed individuals.

Several major hurdles need to be overcome simultaneously to create a CNS active treatment for nerve agent exposure. Chemical moieties that increase passive diffusion across the BBB need to be designed into or encapsulated around the molecule. At the same time, chemical moieties that trigger active cellular export (i.e. P-glycoprotein) need to be minimized or eliminated entirely. In addition, efficacy needs to be increased and toxicity needs to be reduced by respectively enhancing the antidote-nerve agent complex off rates in adducted AChE and abating off-target binding. Satisfying these requirements is a daunting endeavor and the subject of many efforts; several of which show promise^10–28^.

**Figure 1:**
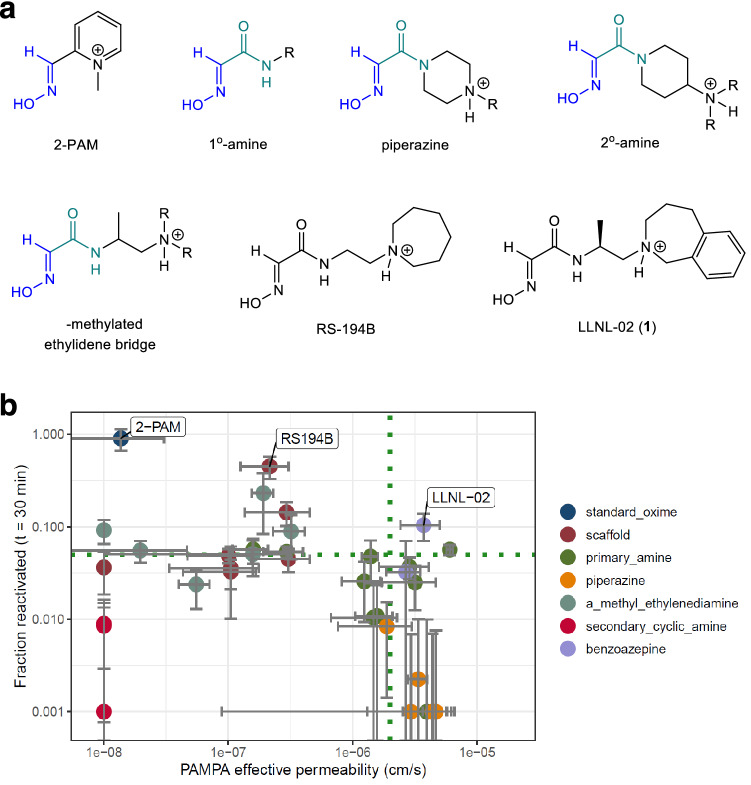
Structures of oxime scaffolds that were explored in our synthetic campaigns. (**a**) Explored chemical moieties are aligned by the oxime warhead. 2-PAM, RS-194B, and LLNL-02 (**1**) compounds are shown for comparison. 2-PAM contains a permanently positively charged quaternary nitrogen atom which limits its passive diffusion across the BBB. LLNL-02 (**1**) and RS-194B both contain a tertiary amine in the azepine ring that is subject to an equilibrium between charged and uncharged chemical species at a synaptic pH of 7.2. All three compounds contain an oxime moiety with *p*K_a_ values of about 8, thereby driving the equilibrium towards a predominantly neutral oxime at pH 7.2. (**b**) The fraction of AChE reactivated is plotted against permeability. Ideal candidates occupy the upper right quadrant, exhibiting properties of both elevated permeability and moderate or better reactivation efficacy. Reactivation of NIMP-adducted acetylcholinesterase measured 30 min after addition of compound. Compound class is specified by the indicated color legend, where each compound represented is an analog of the indicated class.

Here, we have used an iterative sequence that links parallel efforts in synthetic chemistry, computationally supervised virtual-library creation or in silico synthesis, computational docking and permeability predictions and in vitro and in vivo capabilities (Figure S1) that has resulted in the identification of a promising reactivator candidate. This cyclical approach seeks to balance the competing aims of achieving reactivation activity and BBB permeability, toward simultaneous development of compounds that exhibit both properties^29,30^. Each capability feeds information back to the others, allowing the near-simultaneous optimization of CNS penetration, antidotal activity, and reduction of toxicity. This iterative and parallel approach ensures efficient use of resources and accelerated optimization of the compound. With this iterative approach, we have designed and characterized a novel oxime molecule, LLNL-02 (**1**), with high BBB penetration and significant reactivation capacity that shows promise for further development (Fig. 1a).

## Results and discussion

In the current work we have developed an iterative workflow for the design and characterization of BBB permeable and active oxime compounds. This approach allows for parallel efforts in computational prediction, chemical synthesis, and bioassays for rapid optimization of two seemingly opposite chemical functions (Figure S1). For example, synthesis of more tractable novel compounds was started at the same time a computational library of virtual compounds was being created. Virtual compounds were ranked according to synthetic tractability. In silico predictions for binding and permeability were made for compounds that were ranked as more synthetically challenging as well as for the more tractable compounds. All synthesized compounds were subjected to in vitro reactivation and permeability assays. The results of these experiments informed the next round of synthesis and in silico predictions. Specifically, for the benzoazepine scaffold described below, our predicted in silico and in vitro permeability data are in good agreement with the in vivo results reported herein.

### General synthetic campaigns to determine structure activity relationship for BBB permeability and AChE reactivation

To improve passive diffusion of non-quaternary amido-oximes across the BBB, five synthetic campaigns were undertaken to determine a structural-activity relationship (SAR) (Fig. 1a). As shown in Fig. 1b, we found that when compound permeability was increased, reactivation activity decreased. From the generated data, we observed four major structural features of the oxime that were important for reactivation and permeability. First, an ionizable nitrogen atom distal to the amide nitrogen was required for significant reactivation, especially when this distal nitrogen was part of a ring system. Second, alkyl ring size was an important feature with larger rings showing greater activity, specifically 7-membered rings were more potent than five or six-membered alkyl rings. Third, an additional chiral methyl group was tolerated between the amide and distal nitrogen atoms and most specifically at the carbon nearest to the amide nitrogen (Fig. 1a). Fourth, addition of a benzo moiety on the azepine ring increased permeability. With the resulting SAR we succeeded in developing a compound with increased permeability while retaining significant reactivation activity which we describe below.

### Prediction of positive determinants for in-line conformational binding and BBB permeability

**Figure 2:**
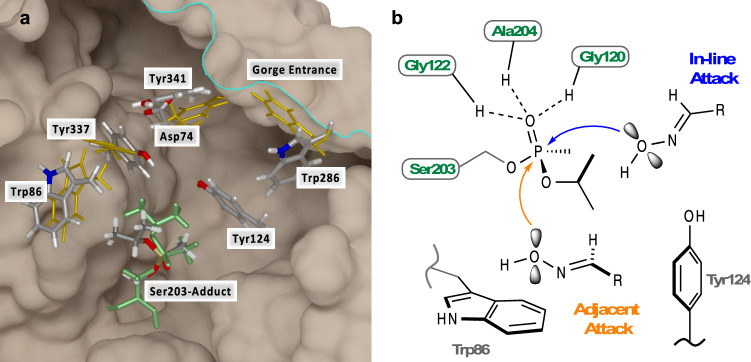
(**a**) Five AChE protein structures used in the docking/rescoring calculations are aligned by key active site residues, [Human Xtal (5FPQ), Human Model (derived from 1B41), Mouse 2Y2V, Mouse 2WHP, and Mouse 5FPP]. The sidechains of several residues that mediate binding of oximes have significant spatial variability. Alternate conformations are shown in gold; Asp74, Trp86, Trp286, Trp337, and Trp341. The isopropyl tail (green) of the adduct is rotated by ~ 50° around the isopropyl-O–P bond, placing the methyl groups closer to the Trp86 sidechain. Reactivator design needs to account for the active site plasticity to prevent unproductive binding. Figure created with VMD^60^. (**b**) Key AChE active site residues with two putative nucleophilic reaction mechanisms. The in-line attack mechanism places the approaching oxime group at 180° to the Ser203 oxygen atom in the Ser-O–P=O plane. The serine residue then becomes the leaving group, and the phosphate-oxime complex can leave the active site or recombine. In the adjacent attack scenario, there are two possible leaving groups, Ser203 and the isopropyl group. If the isopropyl group leaves then the enzyme is not reactivatable. A close interaction of the isopropyl group with the indole sidechain of Trp86 will fill the space available for binding and reduces the possibility of adjacent attack as seen in the 5FPP crystal structure.

To aid the development of the SAR, we computationally enumerated compounds and screened them in silico using five AChE proteins as a target panel and a model lipid bilayer. Each of these protein targets is unique in the sidechain conformations of various active site residues, allowing for scaffold diversity in screening for possible hits as shown in Fig. 2a. These sidechains may serve as gating functions for oxime reactivators similar to what has been reported for other AChE substrates^31–37^. The sidechain gating of the OP-inhibited AChE is not well understood. Based on the 3D crystal structures of AChE used in the study, the Tyr124 position is conserved across all protein targets. Other residues such as Trp286, Asp74, Tyr337, Tyr341, and Trp86 have sidechain positions that appear to be dependent on the presence of a ligand (Fig. 2a).

Two general binding modalities near the phosphorus atom are possible and are illustrated in Fig. 2b. In-line binding of the oxime promotes the removal of the protein from the oxime-adduct complex. Adjacent binding allows for the protein or the isopropyl group to leave the oxime-adduct complex. Thermodynamically, the isopropyl group is the more stable leaving group, therefore oxime binding in the adjacent conformation is not viable in therapeutic design efforts.

Examination of the structures also shows that parallel stacking of the aromatic sidechains of Tyr124 and Trp286 is ligand dependent. In addition, the distance between the hydroxyl groups of Tyr341 and Tyr124 varies across several crystal structures (5.4–7.22 Å). We hypothesize that the variation of this distance in tandem with the interaction between Trp86 sidechain and the adduct isopropyl group modulate the docking pose of the compound to be in either the adjacent (non-productive) or in-line (productive) conformations as illustrated in Fig. 2b. Interestingly, the sidechain orientation of Tyr124 was invariant among all the protein targets, suggesting that protein motion in the region of Tyr341 is important. The Asp74 sidechain also shows significant variation across protein targets as seen in Fig. 2a.

Molecular docking to these five static protein structures reveals possible reasons why compounds may bind in unproductive, adjacent attack poses as well as in the productive, in-line attack conformation. That is, the larger distances between the sidechains of Tyr124 and Tyr341 and the larger distances between the Trp86 sidechain and phosphate adduct accommodate smaller oxime compounds in the adjacent site. The changes in the conformation and dynamics of Tyr341 due to adduct binding has been proposed in previous work^31^. We predict that binding in the adjacent site decreases the efficiency of the actual reactivation reaction by causing the isopropyl group to be the leaving group and/or by locking the nucleophile into an unproductive pose. Moreover, as in vitro data became available, we adjusted our in silico prediction cutoffs for docking/rescoring of candidate hits.

Two of the protein targets, 5FPP and HumanModel, show the that the torsion angle between the methyl and isopropyl groups of the adduct is ~ 50° larger compared to other mouse and human adducted structures^31,38^ as illustrated in Fig. 2a. The larger torsion angle exposes more surface area of the phosphorus atom and promotes a closer interaction between the isopropyl methyl groups and the sidechain of Trp86 by 1 Å thereby promoting an in-line reaction where the leaving group is the Ser203 residue of the protein and not the isopropyl tail of the adduct (Fig. 2b). At the same time, this interaction effectively blocks docking of ligands in an adjacent angle of attack, which we propose as non-productive as it sequesters compounds and may even permanently inhibit the enzyme. Interestingly, this torsional rotation is not observed in the 2WHP protein target which suggests that this alternate conformation is uncommon and may be dependent on protein dynamics after ligand binding. Additional molecular dynamics simulations will address this question. Other protein targets (HumanXtal and 2Y2V) have a larger gap between the sidechain of Trp86 and the adducts’ isopropyl group, allowing for adjacent docking poses to be observed in the computational screening.

**Figure 3:**
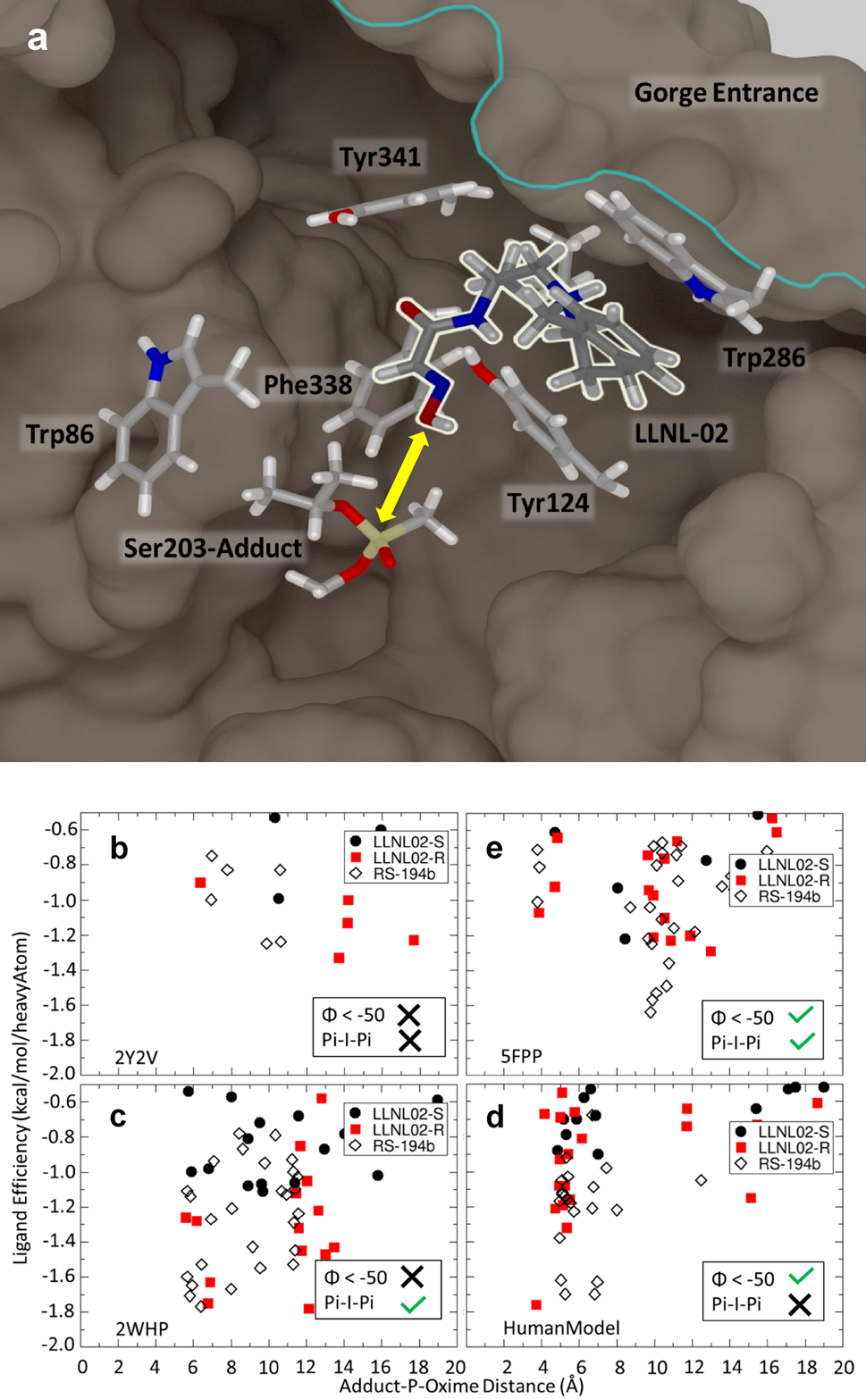
Example LLNL-02 (**1**) pose from the docking calculations. (**a**) AChE active site residues with LLNL-02 docked in an in-line orientation. The addition of the aromatic moiety creates a π–π stacking interaction with Tyr124 and Trp286. A hydrophobic pocket is filled by the LLNL-02 methyl group which interacts with Ile293, Tyr341, and Phe338 in the rat AChE crystal structure (5FPP). The phosphorus-oxime distance is ~ 4.7 Å which approximates an enzymatic encounter complex (yellow arrow). Figure was created with VMD^60^. (**b**) Docking results for the (**c**) 2Y2V, (**d**) 2WHP, (**e**) HumanModel, and (**e**) 5FPP protein targets. LLNL-02 enantiomers and RS-194B ligand efficiencies versus the distance between the oxime oxygen and phosphorous atoms (P–O distance) are shown. The selection of R over S enantiomers in 5FPP and 2WHP is a result of the π–π sandwich interaction. In addition, the shortened P–O distances in the 5FPP and HumanModel targets are a result of the ~ 50° rotation in the torsion angle between the methyl and isopropyl groups of the adduct. This large rotation also shortens the distance between the isopropyl tail and Trp86 sidechain by 1 Å, effectively blocking adjacent binding. The removal of the π–π sandwich interaction in the Human Model allows both LLNL-02 enantiomers to interact with Trp286 with either face of the benzoazepine ring. Together, these results show that the addition of the methyl group and benzo moiety slightly altered ligand efficiencies and P–O distances compared to RS-194B while significantly increasing permeability.

Subsequent in silico studies on LLNL-02 (**1**) revealed several key properties of the molecule that supported its superior reactivation activity relative to previous oximes that showed promising reactivation profiles but lacked the permeability power to become serious candidates for consideration. A docking pose of LLNL-02 that highlights several key features of an optimal in-line docking conformation is shown in Fig. 3a. An anchoring π–π sandwich interaction is made between the aromatic rings of Tyr124 and Trp286 and the benzo moiety of LLNL-02. This interaction stabilizes the more flexible oxime portion of the molecule. Additional stabilization of the oxime is gained by the methyl group adjacent to the amide nitrogen. This methyl group fills a hydrophobic pocket between Tyr341 and Phe338 and Ile293. The distance between the oxime oxygen and phosphorous atom is ~ 4.7 Å, therefore qualifying it as a putative enzyme encounter complex (Fig. 3a).

Interestingly, docking calculations of the *R* and *S*-enantiomers of LLNL-02 (**1**) show a difference in pose location and ligand efficiencies, depending on the protein target as shown in Fig. 3b–e). The protein targets are arranged in a hypothetical binding continuum with 2Y2V (Fig. 3b) and 5FPP (Fig. 3e) as the least and most oxime-accessible conformations, respectively. Results for the HumanXtal target are similar to those of 2Y2V and have been omitted for clarity. The 5FPP and 2WHP protein targets allow for the π–π sandwich interaction, which appears to favor the docking of the *R*-enantiomer (Fig. 3d,e). The torsional rotation of the adduct isopropyl group is only present in the HumanModel and 5FPP targets (Fig. 3c–e) and allows for smaller P-O distances without favoring the docking of one enantiomer over another. Importantly, all oxime poses with P-O distances less than 8 Å are observed to be in the non-productive adjacent orientation in the 2Y2V and 2WHP protein targets. Only the productive in-line attack orientation of the oxime is possible for the HumanModel and 5FPP protein targets. The comparison of ligand efficiencies and P-O distances between the two LLNL-02 enantiomers and RS-194B appear to show a slight preference for RS-194B.

Modulating the *p*K_a_ of the alkylamine is crucial for the increased BBB permeability of LLNL-02 (**1**). The in silico calculations predict that modulating the *p*K_a_ of RS-194B could have a large effect on the permeability of the compound. Therefore, synthetic efforts to increase BBB permeability of the azepine oximes focused on increasing general lipophilicity and reducing the *p*K_a_ of the azepine nitrogen. These efforts resulted in the addition of a chiral methyl group distal to the amide nitrogen and later the fusion of a benzo moiety to the azepine ring. We posit that this increase in predicted permeability is due to the reduction of the *p*K_a_ via electronic effects between the nitrogen lone-pair electrons and π electrons in the benzo moiety. Structures with these adjustments were subjected to docking and permeability calculations and were found to significantly increase predicted permeability (Supporting Information, Figure S3). Observed ligand efficiencies and P-Oxime distances appear to show a slight preference for RS-194B when compared to the values from the LLNL-02 enantiomers. Additionally, the permeability of RS-194B was predicted to be very low whereas LLNL-02 was predicted to be significantly more permeable in comparison. The predicted effective permeability is in good agreement with the PAMPA results shown below.

### Synthetic strategies

Our approach included a synthetic effort based on tractable modifications on the RS-194B scaffold. One of the initial modifications on RS-194B was the introduction of a methyl group at the carbon closer to the amido nitrogen. In introducing this methyl group, we created a chiral center in the molecule. As initially the synthetic route for these compounds (bearing the methyl group) included using the aldehyde version of the amino acid alanine, the stereochemistry at this carbon was set from the outset (i.e*.* (*S*)-enantiomer). A second structural modification that proved to be crucial in the process was the appending of a benzo moiety to the azepine nucleus to yield a benzoazepine cyclic moiety. The purpose of the construction of a benzoazepine ring was to decrease the pK_a_ and increase the clogP value and thus the overall hydrophobicity of the compound with the expectation of increasing BBB permeability. These two initial modifications proved to be successful at yielding an oxime that possesses an increased permeability profile while retaining potency as a reactivator for AChE. This finding highlights the importance of working in parallel on complementary tasks towards the ultimate goal of a candidate compound that could fulfill the highly-demanding characteristics of a CNS-permeable reactivator.

### In vitro assessment of oxime membrane permeability

**Figure 4:**
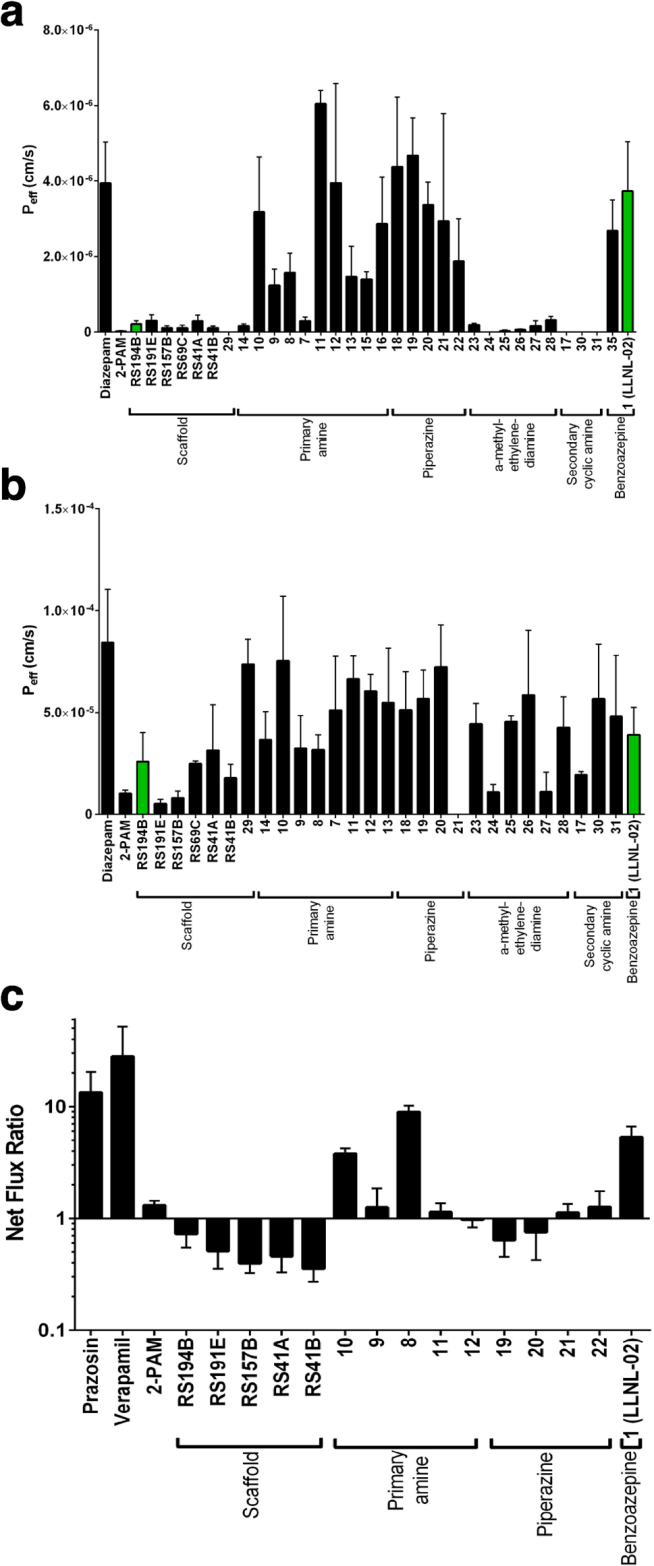
In vitro diffusion and transcellular permeability of oxime candidates. LLNL-02 (**1**) demonstrated moderate diffusion and permeability at levels between that of RS-194B and diazepam. (**a**) The PAMPA system was applied to examine degree of passive diffusion for each compound. Results showed a range of permeability across tested compounds and indicate elevated permeability of LLNL-02. Compounds with quantities in acceptor wells below the UPLC limit of detection are shown as zero. (**b**) A HCMEC monolayer was applied to examine transcellular diffusion across an in vitro representation of the brain endothelium. Results for compound **21** were below the limit of detection for this assay system. Compounds **22**, **15**, **16**, and **35** could not be solubilized and successfully formulated in culture media, and thus were omitted from analysis by this platform. Diazepam is shown as a positive control for permeability. (**c**) The in vitro MDR1-MDCK assay system was applied to assess substrate specificity for efflux by p-gp, for a select subset of compounds. Net flux ratio (NFR) greater than one indicates that a compound may be a possible substrate. Prazosin and verapamil are shown as a positive control for efflux substrate specificity. Compound chemical class is shown below the horizontal axis.

Following the guidance of the in silico model iterations over various functional groups, several candidate compounds from different chemical classes were subsequently synthesized and evaluated by in vitro experiments. The 2-PAM standard of care exhibited maximal reactivation within in vitro models, but extremely low permeability as shown in Fig. 4a,b. Certain scaffold compounds, including RS194B, exhibited reactivation approaching or similar to 2-PAM, with slightly higher permeability. Functional groups such as piperazine and certain primary amines increased the scaffold’s permeability; however, the reactivation capacity was substantially reduced in those cases. Secondary cyclic amines exhibited both low permeability and reactivation. The benzoazepine class characterized by LLNL-02 (**1**) performed reasonably well in both permeability and reactivation, thus breaking the noted trend towards lower reactivation as permeability increased as shown in Fig. 1b. These results agree with the in silico binding and permeability predictions of LLNL-02 when compared to RS-194B (Fig. 3b).

**Figure 5:**
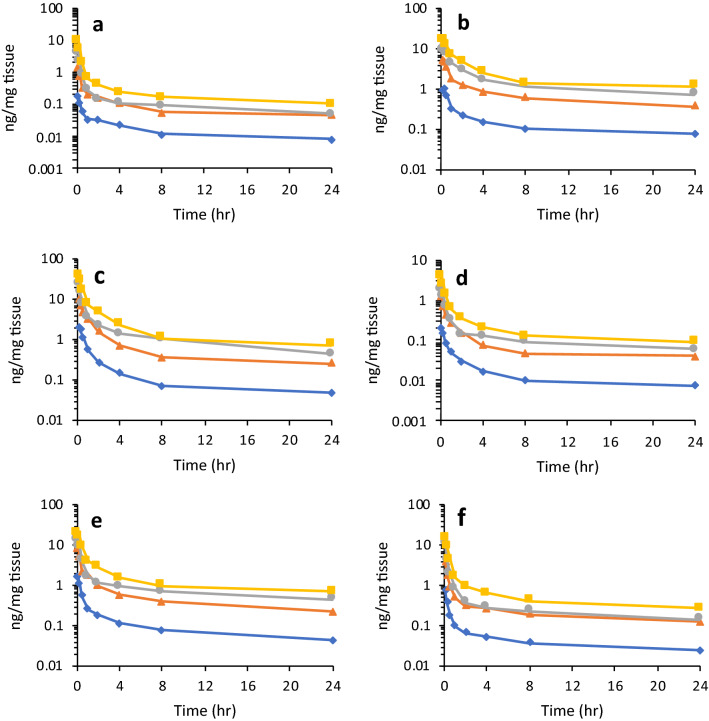
Mean concentration–time profiles of LLNL-02 in (**a**) brain; (**b**) liver; (**c**) kidney; (**d**) heart; (**e**) lung; (**f**) spleen following a single, intravenous administration of 1 (blue diamond), 5 (orange triangle), 10 (grey circle), or 20 (yellow square) mg/kg ^14^C-LLNL-02 to male guinea pigs. Data are expressed as the mean of 6 animals ± the standard error.

A variety of factors have the potential to affect the capacity of a given compound to cross the BBB. This barrier is composed of tightly-apposed microvascular endothelial cells, which limits molecular traversal. Access across this barrier may occur through diffusion or active transport, depending on the molecular properties of a given compound. This permeability may, however, be further limited by the presence of efflux pumps such as p-glycoprotein (p-gp), also termed multidrug resistance protein 1 (MDR1)^39^. To examine each of these properties, three metrics of permeability were assessed to infer and predict compound behavior at the BBB. The PAMPA system was applied to assess membrane diffusion; human cerebral microvascular endothelial cell (HCMEC) traversal was used to examine cell diffusion and active uptake; and the MDR1-MDCK assay was employed to assess p-gp efflux substrate specificity and the results are described in Fig. 5a,b. For several compounds in the current work, RS-194B^14^ was employed as an initial scaffold for further iteration and thus was evaluated with all other compounds in each of the assays described below.

#### PAMPA assay

The PAMPA system is composed of a lipid–oil–lipid trilayer intended to emulate passive diffusion across a lipid membrane for given compound. Transfer of compound from the donor side to the acceptor side of the membrane was assessed, and effective permeability was calculated for each compound. Diffusion in this system ranged from undetectable to high levels of permeation (Fig. 4a). Permeability of LLNL-02 (**1**) (3.7 ± 1.3 × 10^–6^ cm/s) across the lipid membrane was similar to that of diazepam (3.9 ± 1.1 × 10^–6^ cm/s), a known CNS-permeable therapeutic compound, and a magnitude more permeable compared to the initial RS-194B scaffold (2.1 ± 0.8 × 10^–7^ cm/s) (P < 0.0001).

#### HCMEC traversal

Transcellular diffusion and active transport were assessed by examining permeability across an in vitro representation of the brain endothelium. This system was composed of an HCMEC/D3 monolayer. Each compound was incubated in the donor well and traversal to the acceptor well was quantified by ultra-performance liquid chromatography (UPLC). Four compounds (**22**, **15**, **16**, and **35**) included in PAMPA testing were omitted from HCMEC testing due to challenges related to solubilization or formulation compatibility with the endothelial tissue culture platform. Effective endothelial permeability was subsequently calculated (Fig. 4b). Endothelial permeability of LLNL-02 (**1**) (3.9 ± 1.4 × 10^–5^ cm/s) was reduced relative to diazepam (8.4 ± 2.6 × 10^–5^ cm/s) (0.5-fold, P = 0.0003) and elevated relative to 2-PAM (1.0 ± 0.2 × 10^–5^ cm/s) (3.9-fold, P = 0.0007). LLNL-02 endothelial permeability was elevated relative to RS-194B (2.6 ± 1.4 × 10^–5^ cm/s); however, this difference was not statistically significant at P < 0.05 (P = 0.07).

#### MDR1-MDCK assay

Despite elevated diffusion and active transport, CNS access for a compound may be further affected by p-gp efflux pump activity. Efflux substrate specificity was therefore tested for a selected subset of compounds that exhibited promising empirical properties. Effective permeability was markedly reduced for a-methyl ethylenediamines and secondary cyclic amines (Fig. 4c). These compounds were therefore not identified as viable candidates and were omitted from further testing. Efflux was tested using the established MDR1-MDCK tissue culture system with a net flux ratio (NFR) greater than one indicating possible substrate specificity. Prazosin and verapamil were both tested as positive controls for efflux. NFR of LLNL-02 (**1**) was observed at 5.3 ± 1.3. These results suggest that, while LLNL-02 is not a strong substrate for p-gp, its permeability may be affected by efflux. The previously described HCMEC assay does employ a cell line (HCMEC/D3) documented to express the p-gp efflux pump^40^. Our observation of elevated permeability for LLNL-02 in the HCMEC assay relative to the 2-PAM standard of care, despite potential occurrence of efflux, indicate that this compound retains value for future study. Further, delivery could potentially benefit from co-administration with efflux inhibitors; however, this could also result in elevated organophosphate penetrance in the CNS, thus such an approach would need to be carefully considered in context of the nature of exposure, as it could be detrimental to overall outcome. These results also suggest a possible explanation for LLNL-02 demonstrating high permeability in the PAMPA platform, while not exhibiting the highest observed brain endothelial cell permeability relative to other compounds.

### Acetylcholinesterase reactivation capacity

In parallel with the permeability assays, newly synthesized compounds were screened for capacity to reactivate nerve agent-adducted AChE. Initial screening assays were performed using the GB (sarin) surrogate nitrophenyl isopropyl methylphosphonate (NIMP). Reactivation was assessed via measurement of substrate cleavage using a modified Ellman’s assay. For purposes of direct comparison within this study, fraction-of-enzyme reactivated relative to control AChE was examined at an endpoint of 30 min following introduction of candidate reactivators. Although the endpoint method does not yield absolute kinetic data, it is regarded as an acceptable approach for screening larger numbers of candidates for relative evaluation and down-selection^41^. Given that the aim of the study was to identify permeable reactivators anticipated to exhibit elevated CNS access, reactivation efficacy at 30 min for each tested compound was plotted relative to PAMPA-defined effective permeability and the data is shown in Fig. 1b. Achievement of optimal parameters would result in a compound being assigned to the upper right quadrant of Fig. 1b. The compounds assessed demonstrated lower reactivation in each case relative to 2-PAM, which was selected for comparison in this study due to its status as the only oxime compound approved by FDA. The anticipated and empirically-observed result was that heightened permeability resulted in a trend toward reduced reactivation efficacy. Correspondingly, those compounds with high efficacy, including the 2-PAM standard of care, exhibit lower measured in vitro permeability.

As represented in the upper-right quadrant of Fig. 1b, LLNL-02 (**1**) was the only compound retaining some success related to both evaluated parameters. AChE reactivation capacity is critical to achieving the therapeutic aim of these tested compounds. Aside from 2-PAM and the RS194B and RS41A scaffolds, only one tested compound (**23**) demonstrated higher reactivation than LLNL-02. Relative to this compound (**23**), LLNL-02 demonstrated > 10-fold higher PAMPA permeability. Given the balance of promising observations from the full in vitro pipeline, LLNL-02 was selected for further in vitro and in vivo characterization, due to its profile exhibiting high membrane permeation and elevated endothelial cell permeation while maintaining moderate function as a reactivator.

### LLNL-02 in vitro reactivation and metabolic stability

#### GB and VX-adducted acetylcholinesterase reactivation by LLNL-02 (1)

To confirm in vitro efficacy of LLNL-02 against nerve agent exposure, reactivation of adducted AChE was measured at reactivator concentrations ranging from 2 mM to 18 µM over the course of approximately 30 min (Table 1). Testing was performed for both GB- and VX-adducted enzyme. The maximum reactivation percentage observed was 9.5% for GB-inhibited enzyme (measured at 33.2 min) and 24.8% for VX-inhibited enzyme (measured at 28.9 min) with 2 mM compound at the final examined timepoint. Measurements were used to calculate the reactivation rate (*k*_*r*_), binding dissociation constant (K_D_), and overall reactivation rate (*k*_*r2*_) (Table 1). Historical reactivation kinetic data for 2-PAM^42^, measured under identical in vitro assay conditions (with the exception of DMSO inclusion), are shown for comparison. LLNL-02 displayed similar binding affinity relative to 2-PAM but a reduced reactivation rate, thereby resulting in a reduced overall reactivation rate, consistent with results obtained using the NIMP surrogate.

**Table 1:** Reactivation of nerve agent adducted acetylcholinesterase by LLNL-02 (**1**) and 2-PAM

Agent	Reactivation rate, *k*_*r*_ (min^−1^)		Dissociation constant, K_D_ (µM)		Overall reactivation rate, *k*_*r2*_ (min^−1^ M^−1^)	
	2-PAM	LLNL-02	2-PAM	LLNL-02	2-PAM	LLNL-02
GB	0.06 ± 0.02	0.004 ± 0.0001	706 ± 135	551.4 ± 61.3	81 ± 16	6.8 ± 0.8
VX	0.06 ± 0.005	0.01 ± 0.0007	541 ± 155	656 ± 84	121 ± 29	20.4 ± 2.9

Oxime concentration ranged from 18 µM to 2 mM. Reaction time was 30 min.

#### In vitro stability and cytochrome P450 inhibition assessment

The stability of LLNL-02 (**1**) was assessed in vitro in human plasma and human liver microsomes as described in detail previously^43^. Table S1 shows that in plasma, greater than 90% of the parent compound remains after a one-hour incubation at 37° C. Substrate depletion experiments in human liver microsomes revealed a half-life of greater than 60 min, indicating good stability of LLNL-02 in this matrix. P450 inhibition studies showed LLNL-02 to have IC_50_ values of more than 10 μM in 3 of the 5 enzyme/substrate assays, indicating poor inhibition (Table S1). The remaining two assays (CYP3A4/BFC and CYP2D6/AMMC) showed moderate inhibition. Overall, LLNL-02 was determined to be a poor/moderate inhibitor of P450 in the isoforms tested.

### In vivo pharmacokinetics, tissue and brain distribution in guinea pigs

#### In vivo PK

Given the promising outcomes observed in vitro, the pharmacokinetic (PK) parameters of LLNL-02 (**1**) were evaluated in vivo in the guinea pig model. PK was assessed over a 20-fold dose range that was designed to capture the potentially feasible therapeutic doses. The mean PK parameters are presented in Table 2. Across the four doses studied, the mean apparent distribution half-life (t_1/2α_) ranges from 0.25 to 0.4 h, and the terminal half-life (t_1/2 β_) spans from 9.61 to 21.92 h. The t_1/2α_ is similar to what has been reported for the current standard of care 2-PAM of 0.31 h^44^ whereas, the t_1/2 β_ is 2–3 times longer than the 4.6 h-5.0 h range that has been observed for 2-PAM (unpublished data). Total clearance of LLNL-02 from plasma (CL) ranges from 167.77 to 335.60 mL/h/kg and the apparent volume of distribution (V_d_) is 3747.90 to 5341.0 mL/kg, suggesting rapid and extensive distribution beyond the plasma compartment. Power regression analysis revealed that the AUC_0-t_ is linear across the four doses examined with an R^2^ value of 0.9229 (Figure S2), indicating linear first-order kinetics.

**Table 2:** Mean pharmacokinetic parameters of LLNL-02 (**1**) following a single IV administration of ^14^C-LLNL-02 to male guinea pigs.

Dose (mg/kg)	t_1/2α_ (h)	t_½β_ (h)	AUC_0-t_ (µg h/mL)	AUC_0-inf_ (µg h/mL)	V_d_ (mL/kg)	CL (mL/h/kg)
1	0.39 ± 0.01	21.92 ± 3.49	3.88 ± 0.42	7.20 ± 1.14	4402.28 ± 247.12	167.77 ± 46.53
5	0.40 ± 0.02	17.13 ± 2.69	20.52 ± 1.93	31.50 ± 4.61	3747.90 ± 228.94	178.64 ± 35.69
10	0.25 ± 0.03	9.61 ± 0.88	25.87 ± 2.15	30.50 ± 3.39	5132.86 ± 374.57	335.60 ± 34.98
20	0.39 ± 0.06	18.94 ± 5.75	51.72 ± 6.28	87.88 ± 25.49	5341 ± 473.22	311.07 ± 74.71

Data is expressed as the mean of six animals ± the standard error.

#### Tissue distribution

Analysis of ^14^C-LLNL-02 in tissue revealed time and dose dependent concentrations in all tissues examined (Fig. 5A–F). After intravenous (IV) administration, the initial biodistribution of LLNL-02 was rapid with the tissue C_max_ occurring between 5 and 15 min post dose for all tissues examined. The concentrations of LLNL-02 in the tissues varied with the highest concentrations per gram of tissue occurring in the kidney and lung, followed by liver, spleen, brain and heart for all dose concentrations tested (Fig. 5A–F).

#### Brain penetration

The C_max_ of LLNL-02 in the brain occurred at the first sampling time-point of 5 min post dose with mean values ranging from 0.19 to 10.04 ng/mg tissue over the four doses (Fig. 5A), indicating rapid distribution to the brain. The 4.25 ng/mg tissue observed for the 10 mg/kg dose was 20 times higher than what was observed for the similar RS-194B oxime at the same dose level^30^. In comparison to the current standard of care, a dose of 50 µmol 2-PAM in rats resulted in a C_max_ brain concentration of 1.17 ng/mg tissue^45^. A dose–dependent brain/plasma ratio was also observed, indicating dose dependent differences in brain and plasma LLNL-02 concentrations (Table 3). The brain/plasma ratio decreased by an average of 62.7% over the time course of the experiment (0.08 h–24 h) for the 4 doses examined indicating rapid elimination from the brain and back into the bloodstream.

**Table 3:** Brain-plasma ratio of LLNL-02 (**1**) at T_max_ following a single IV dose of ^14^C-LLNL-02 to male guinea pigs.

LLNL-02 dose (mg/kg)	Brain/plasma	logBB
1	0.132	− 0.879
5	0.224	− 0.650
10	0.488	− 0.311
20	0.529	− 0.276

Based on the increase in brain penetration (compared to 2-PAM) together with the favorable pharmacokinetic data of short half-life, suitable biodistribution and rapid clearance, LLNL-02 has the potential to be considered as a viable candidate for treatment of nerve agent exposure. However, more studies to examine the efficacy and toxicity are required before a determination can be made on the usefulness of LLNL-02 as a treatment for nerve agent exposure.

The increase in transcellular traversal across an in vitro representation of the brain endothelium by LLNL-02, compared to 2-PAM and other previously characterized oximes^29,30^, validated the in silico model predictions and correlated well with the observed in vivo data showing higher concentrations of LLNL-02 in brain tissue. Analysis of brain tissue revealed a C_max_ brain:plasma ratio of 0.488 at a dose of 10 mg/kg LLNL-02. This ratio is higher than the 0.138, 0.06, and 0.017 ratios that has been reported for 2-PAM^45–47^, and the similar non-quaternary oxime RS-194B (0.04)^30^. With an administered dose of 10 mg/kg LLNL-02, we observed a 20 × increase in brain accumulation compared to the starting scaffold, equating to 0.61% of the total administered dose^30^.

This increase in permeability was accompanied by a moderate reduction in observed in vitro reactivation (12% relative to 2-PAM after 30 min (Fig. 1; Table 1), 23% relative to 2-PAM after 4 h). The reduced overall reactivation rate for LLNL-02 for both GB and VX compared to 2-PAM is most likely due to the benzo moiety of LLNL-02 intended to enhance permeability. However, it is anticipated that the increase in brain penetration would offset the decrease in activity by substantially improving the quantity of bioavailable compound in the CNS, thereby amplifying LLNL-02’s overall effectiveness as a CNS reactivator. Additional studies are needed to verify this postulate. In addition, ADMET analysis of our compound has shown no major stability or toxicity issues, as well as favourable results from P450 inhibition studies, thus warranting increased characterization of LLNL-02 as a molecular scaffold without the use of extra encapsulation techniques (Table S1).

## Conclusion

We have developed a nearly end-to-end pipeline for discovery and screening of potential CNS-permeable reactivators. This iterative approach utilizes a combination of parallel chemical and in silico syntheses, computational modeling, and a battery of detailed in vitro and in vivo assessments. Use of this pipeline has greatly increased the efficiency in which potential therapeutics can be developed. Our results have identified a novel CNS-permeable oxime reactivator (LLNL-02) with favorably results in both in vitro and in vivo tests. Further studies will examine the in vivo efficacy and toxicity of LLNL-02 to determine if future development is warranted.

## Experimental section

### Chemicals

All reagents were used as received from commercial vendors and used as such. Ethanol and hydroxylamine hydrochloride were purchased from Alfa Aesar (Ward Hill, MA). Ethyl glyoxylate and heparin sodium salt were purchased from Sigma-Aldrich (St. Louis, MO). Collagenase Type IV was obtained from Gibco, (Waltham, MA). ^14^C-labeled ethyl glyoxylate was purchased from American Radiolabeled Chemicals, Inc. (St. Louis, MO). Deuteriochloroform (CDCl_3_), methanol-_d4_ and DMSO-_d6_ were purchased from Cambridge Isotope Laboratories (Tewksbury, MA). 1-(Azepan-1-yl)propan-2-amine, 1-(piperidin-1-yl)propan-2-amine, (2-aminopropyl)dimethylamine, (2-aminopropyl)diethylamine, α-methyl-1-pyrrolidineethanamine, α,4-dimethyl-1-piperidineethanamine, were purchased from Enamine Building Blocks (Cincinnati, OH). 2-(*S*)-2-(Boc-amino)propanal (Boc-ala-aldehyde), 4-Piperidinopiperidine, were purchased from Combiblocks (San Diego, CA.). Aminocyclopropane, aminocyclobutane, 1-aminobutane, benzylamine, homopiperazine and (*S*)-( +)-α-Methoxy-α-(trifluoromethyl)phenylacetyl chloride (Mosher’s acyl chloride) were purchased from Sigma-Aldrich (St Louis, MO). Ethyl glyoxylate oxime and all RS-based compounds were synthesized according to Sit et al.^14^ and obtained as a pale-yellow liquid that was stored at 4 °C and only taken out when used.

### General synthetic procedure

The prediction-set of compounds used in this study was synthesized via an amide-forming reaction between an amine and an ethyl ester species as described previously^29^. Briefly, the library of generated materials can be assembled from an ethyl ester and a series of amines that serve as the point structural-diversity origin. Since the oxime ethyl glyoxylate is not an activated species, the amine was used in slight excess to it (1.2 equiv.) and the mixture was heated to 70 °C in an overnight basis in ethanol. In a few instances, the products precipitated out of the solution (purity > 99% after ethanol washes during filtration) while in some cases flash column chromatography employing a range of gradient combinations was needed for their purification. Purity of the compounds was assessed by ^1^H NMR and in all cases found to be > 98%. The synthetic procedure for LLNL-02 (**1**) is shown in Figure S4.

### Synthetic procedure for LLNL-02

#### LLNL-02 (1)

(*S*)-1-(1,3,4,5-Tetrahydro-2*H*-benzo[*c*]azepin-2-yl)propan-2-amine **5** (1.5 g, 7.35 mmol) was dissolved in ethanol (100 mL) along with ethyl glyoxylate oxime **6** (1.08 g, 9.2 mmol, 1.25 equiv. to **5**) in a 250 mL round bottom flask equipped with a stir bar (Figure S4). The resulting solution was heated to 70 °C overnight. The mixture was transferred to a separatory funnel and partitioned (H_2_O//DCM). The aqueous phase was extracted with DCM (2 × 50 mL) and then the combined organic phases were extracted with brine (NaCl/H_2_O, 3 × 50 mL), dried over anhydrous sodium sulfate and evaporated in vacuo at 60 °C to give an amber-brown solid that was purified by flash column chromatography (DCM → 2:8 MeOH/DCM) to furnish **1** as an off-white solid (0.73 g, 36%). R_*f*_ (1:9 MeOH/DCM): 0.68; ^1^H NMR (DMSO-_d6_, 600 MHz) δ 11.84 (s, 1H, N–OH), 7.76 (d, *J* = 7.8 Hz, 1H), 7.4 (s, 1H), 7.11–7.08 (m, 4H), 3.98–3.93 (m, 1H), 3.85–3.79 (m, 2H), 3.07–3.01 (m, 2H), 2.86–2.79 (m, 2H), 2.30 (dd, *J* = 12.6, 7.8, 1H), 2.19 (dd, *J* = 12.6, 7.8, 1H), 1.58–1.57 (m, 2H), 1.00 (d, *J* = 6.6, 3H, *CH*_*3*_); ^13^C NMR (DMSO-_d6_, 151 MHz) δ 161.6 (C=O), 144.3 (C=N–OH), 143.1, 139.7, 130.0, 129.1, 127.4, 126.1, 59.2, 58.7, 56.9, 42.8 (*C*–H), 35.9, 24.8, 19.2 (CH_3_); Anal. Calcd. for C_15_H_21_N_3_O_2_: C, 65.43; H, 7.69; N, 15.26; found: C, 65.32; H, 7.55; N, 15.11; HRMS (CI) *m/z* calcd for C_15_H_21_N_3_O_2_ [M^+^]: 275.1634; found 275.1632.

### Radiolabeling of LLNL-02

^14^C-LLNL-02 (specific activity = 2.5 μCi/mmol) was synthesized in two steps using ^14^C-labeled ethyl glyoxylate as a precursor. The radiolabeled product was synthesized as follows: ethyl glyoxylate (50% in toluene, 9.2 g, 45.1 mmol, 102.1 g/mol) and 0.5 mL of ^14^C-labeled glyoxylate (~ 1.7 mg, 0.017 mmol, 0.250 mCi/mL, specific activity = 7.5 mCi/mmol) were dissolved in a 9:1 acetonitrile:water (50 mL) solution in a 100 mL round bottom flask equipped with a stir bar treated with hydroxylamine hydrochloride (3.13 g, 45.1 mmol, 69.49 g/mol). The resulting suspension was treated with triethylamine (6.35 mL, 45.1 mmol, 101.2 g/mol, 0.726 g/mL) dropwise over 10–20 min using an addition funnel and then stirred at room temperature overnight. The solvents were removed in vacuo at 70 °C for 1–2 h to yield a semisolid oily residue. This semisolid was dissolved with 10 mL water and the aqueous phase was washed with diethyl ether (3 × 15 mL). The organic phase was dried over anhydrous Na_2_SO_4_, suction filtered, and evaporated in vacuo to yield the crude oxime-ethyl glyoxylate. The crude oxime-ethyl glyoxylate (350 mg, 2.96 mmol, 1.5 equiv.) and *N*-2- aminoethylbenzoazepine(400 mg, 1.96 mmol, 1 equiv.) were dissolved in ethanol (5 mL) in a 50 mL glass vial equipped with a stir bar. The resulting mixture was stirred at 70 °C overnight. The mixture was then cooled to room temperature and the solid was suspended in 10 mL of cold ethanol. The fine white solid was collected after filtration, washed with cold ethanol (3 × 10 mL), dissolved in 50% methanol-dichloromethane (MeOH/DCM; 2 mL), purified by silica column chromatography (5% MeOH/DCM mobile phase), and clean product fractions were dried in vacuo for 1–2 h. Radiopurity was assessed by HPLC and liquid scintillation counting. The product was determined to be 98% pure.

### General synthetic procedure for remaining oximes

The coupling procedure between the amine and ethyl glyoxylate oxime is the same as the one developed by Radić et al.^26^ Most of the products obtained by this protocol did not precipitate out of the reaction mixture, and, as such, a work-up procedure involving the partitioning of the mixture (in EtOH) between H_2_O and DCM was needed. The aqueous phase was washed with DCM (2 × 40 mL), and then the combined organics were washed with brine (NaCl/H_2_O, 2 × 50 mL), dried over anhydrous sodium sulfate and evaporated in vacuo at 60 °C to yield a brown or amber residue. In all cases, except for the ones where the product precipitated out of the solution, flash column chromatography using the Biotage purification system was employed to purify the material using EtOAc and hexanes or methanol and dichloromethane as solvent pairs. Synthetic routes specific to each compound are given in the SI.

### In silico docking and permeability predictions

Virtual libraries of test compounds were created using RDKit (https://www.rdkit.org) and our high-performance computing resources. The nucleophilic amino-oxime was maintained while additional moieties were added via known chemical reactions in coordination with our synthetic chemist. The reagent structures were downloaded from the zinc15 database (https://zinc15.docking.org) to increase the probability of rapid synthesis and testing. Protein targets were taken from the PDB (2Y2V, 2WHP, and 5FPP) and from previous published homology models which were based on 1B41 (HumanModel) and early structure of 5FPQ (HumanXtal) provided by Dr. Fredrik Ekström (Personal Communication, 2014)^31,48^. The ConveyorLC workflow was used to prepare the proteins and ligands^49^ for high-throughput docking/rescoring. Compounds were selected with predicted poses that satisfied the following rules: (1) oxime oxygen to sarin phosphorus atom distance < 8 A, (2) in-line attack conformation with an angle of 180 degrees between Ser203 hydroxyl oxygen-phosphorus-oxime oxygen atoms, (3) ligand docking efficiency between − 2 and − 0.9 kcal/mol/#heavy atoms (this is the average value for currently fielded oximes in our calculations). Selected compounds from the docking steps were then screened for permeability. Procedures for the permeability calculations followed those given in^29,50^. cpKa values were calculated using ChemDraw (Chemoffice 16).

### In vitro assays

#### PAMPA assay

Passive diffusion of LLNL-02 was assessed using the Gentest Precoated PAMPA Plate System (Corning Discovery Labware). The system is composed of a 96-well plate/insert system. Two fluid-filled compartments (donor well and receiver well) are separated by a polyvinylidene fluoride (PVDF) filter plate precoated with a phospholipid-oil-phospholipid trilayer primarily consisting of DOPC phospholipids^51^. Each compound was dissolved at 100 μM in Hanks Balanced Salt Solution (HBSS) and added to the lower (donor) well of the plate, followed by addition of the insert to the plate. The system was incubated for 5 h at 25 °C, after which buffer was removed from the lower (donor) and upper (acceptor) well. Five replicate wells were employed in each experiment. Each compound was tested in two independent experiments. All compounds were quantified by UPLC separation and UV detection, and effective permeability (cm/s) was calculated. All UPLC detection protocols and calculation parameters have been previously described^29^.

#### Brain endothelial cell traversal

Traversal of LLNL-02 across brain endothelial cells was assessed using human cerebral microvascular cells (HCMEC/D3), obtained from Cornell University. The cells were checked for mycoplasma before shipping, and ID confirmed by staining for cell surface receptors after receipt. Cells were grown in EndoGRO-MV Complete media (EMD Millipore). For transcellular assays, cells were seeded at approximately 5 × 10^4^ cells/cm^2^ in 12-well transwell tissue culture inserts (0.4 μm pore size, polycarbonate membrane) (Corning Life Sciences). All inserts were collagen coated prior to use. Cells were seeded in media containing 10 mM LiCl, and media was exchanged every 2–3 days. Monolayer integrity was monitored continuously via transendothelial electrical resistance using the EVOM2 volt-ohmmeter (World Precision Instruments). Inserts were used in assays 6–7 days following seeding. Compound was dissolved at 100 μM in HBSS for testing and added to the upper (apical) well of the insert. Buffer samples were removed from the lower (basolateral) well at 0, 15, 30, 60, 90, and 120 min after dosing. Each compound was tested in at least triplicate in 1–2 experiments, with each replicate representing an independent well containing a distinct cell monolayer. As above, compound in buffer was quantified by UPLC as previously described^29^. Permeability of each compound across inserts not containing cells was examined as a control in each assay plate. Effective permeability (P_eff_ [cm/s]) was calculated using the permeability surface area product method, as described by^52^.

#### MDR1 efflux assessment

Efflux capacity of LLNL-02 was assessed in MDR1-MDCK and MDCK parent cells, obtained from the National Institutes of Health (Bethesda, MD). Both cell types were grown in Dulbecco's modified Eagle's medium (DMEM) containing glucose (4.5 g/L) and sodium pyruvate (110 mg/L), supplemented with 10% fetal bovine serum (FBS), l-glutamine (5 mM), and penicillin/streptomycin (50 U/mL; 50 μg/mL). The cells were checked for mycoplasma before shipping, and ID confirmed by staining for cell surface receptors after receipt. MDR1 expression was maintained in MDR1-MDCK cells through culture in the presence of colchicine (80 ng/mL). For traversal studies, cells were seeded at approximately 5 × 10^5^ cells/cm^2^ in 24-well HTS Transwell supports (0.4 μm pore size, polycarbonate membrane) (Corning Life Sciences). Media was exchanged every 2–3 days, and cells were used 5–6 days after seeding. For testing, compounds were dissolved at 100 μM in HBSS and added to the donor well. Transport was examined in both the A to B (apical to basolateral) and B to A (basolateral to apical) directions. Each tested compound was processed in at least triplicate in 1–2 experiments, with each replicate representing an independent well containing a distinct cell monolayer. Transport was examined in both the A to B (apical to basolateral) and B to A (basolateral to apical) directions. The transport experiment was run for 180 min at 37 °C, 5% CO_2_. Buffer was sampled from the acceptor compartment (apical or basolateral, dependent on transport condition). This experiment was performed in both MDR1-MDCK cells and control MDCK parent cells. The efflux ratio was calculated as the ratio of permeability in the B to A direction to the permeability in the A to B direction. As above, quantity of compound in buffer was quantified by UPLC as previously described^29^. Results are represented as the net flux ratio (NFR), calculated as the efflux ratio in MDR1-MDCK cells to efflux ratio in MDCK cells. NFR value greater than one indicates that the compound is a possible substrate for efflux by p-glycoprotein (MDR1). The calculations outlined above were performed as described by^53^.

#### Acetylcholinesterase reactivation screening

A modified Ellman’s assay was adapted for assessment of AChE reactivation by candidate oximes. Purified acetylcholinesterase enzyme (erythrocytic origin, ≥ 500 U/mg protein), acetylthiocholine, 5,5′-dithiobis-2-nitrobenzoic acid (DTNB), and 2-PAM were obtained commercially from Sigma-Aldrich. The surrogate, nitrophenyl isopropyl methylphosphonate (NIMP) was synthesized according to previously documented methods^54^. AChE enzyme was diluted in phosphate-buffered saline pH 7.4 (PBS). NIMP was added to this solution at a concentration achieving 1.2X molar equivalents relative to AChE. Enzyme was incubated at 25 °C for 15 min. Excess NIMP was removed by filtration through a 10 kD MWCO cellulose membrane filter unit (EMD Millipore), followed by two washes with PBS. Resultant enzyme was resuspended at a concentration of 2 mU/µL. A non-inhibited AChE control aliquot was subjected to identical filtering and washing procedures. AChE (0.5 mU/µL final concentration) and oxime (100 µM final concentration) were combined in wells of a 96-well plate. At 30 min following initiation of the reactivation, the reaction mix was diluted 1:20 into the substrates acetylthiocholine (1 mM) and DTNB (1 mM). Enzyme activity was examined by measuring absorbance (410 nm) at 25 °C using a 96-well microplate reader. Activity of reactivated enzyme was normalized to control AChE measurements taken in the same plate. Initial reactivation screening was performed using four replicates in one experiment. Candidates exhibiting positive reactivation profiles were prioritized for additional confirmatory testing using at least one additional experiment of four replicates. Reactivation profile of LLNL-02 was confirmed using six independent experiments of four replicates each. Control wells were included in each plate to confirm successful inactivation of AChE and assessment of spontaneous substrate hydrolysis. Potential for oximolysis was tested for selected oximes by addition of 100 µM oxime to acetylthiocholine/DTNB. Possible inhibition of AChE by oxime was tested by addition of 100 µM oxime to fully active AChE at 0.5 mU/µL. No appreciable oximolysis or inhibition was observed under these conditions.

Additional details for the creation of Fig. 1B are as follows. Both axes are shown on a log_10_ scale. For graphical representation, candidates with a negative fraction reactivated (due to negative measured activity relative to control) were assigned a fraction reactivated value of 0.001, and candidates with permeability below the limit of detection for the PAMPA platform were assigned an effective permeability value of 1 × 10^–8^ cm/s. To similarly facilitate visualization, error (standard deviation) bar lower bounds with negative values were bounded at zero. The horizontal dashed line is placed at a reactivation level of 5%, while the vertical dashed line is placed at a PAMPA effective permeability of 2 × 10^–6^ cm/s. Reactivation of NIMP-adducted acetylcholinesterase measured 30 min after addition of compound.

#### Reactivation of agent-adducted acetylcholinesterase

To achieve a thorough and independent confirmation of the capacity of LLNL-02 for reactivation of VX- and GB-adducted AChE, additional assays were performed at the US Army Medical Research Institute of Chemical Defense (USAMRICD). These agents are extremely dangerous and should only be used in strict accordance with regulations from the Organization for the Prohibition of Chemical Weapons (OPCW). For all assays, three replicates for every sample were individually analyzed in a row-dependent manner and resulting kinetic parameters for each replicate were averaged to generate the value and the standard errors for the experiment. For 2-PAM, because it was the field-standard for comparison, three independent experiments were performed for each agent and the resulting kinetic parameters were averaged to generate the resulting values (previously reported in Cadieux CL et al. Chemico-Biological Interactions, 2016. 259(B)). For LLNL-02, one independent experiment for each agent was conducted as described previously by Cadieux et al.^42^ using recombinant human AChE (AT1002, Allotropic Tech, LLC, Halethorpe, MD). In brief, enzyme was inhibited with a molar excess of nerve agent and excess free nerve agent was removed using small scale size-exclusion columns. The resulting inhibited sample and an uninhibited positive control sample were diluted to an appropriate working concentration. Enzyme samples were mixed with reactivator compound at concentrations ranging from 2 mM to 18 µM to initiate reactivation, and aliquots were removed at various time points, diluted 20-fold and measured for activity. Percent reactivation was calculated by comparing each sample to a row-matched positive control. Nonlinear regression assuming maximal reactivation of 100% was used to determine *k*_*obs*_^14,55^ at each reactivator concentration. These *k*_*obs*_ values were plotted against reactivator concentration and fit using a modified Michaelis–Menten equation to calculate reactivation rate (*k*_*r*_) and dissociation constant (*K*_*D*_)^56^. Overall reactivation rate (*k*_*r2*_) was then calculated using these values.

#### In vitro stability and cytochrome P450 inhibition assessment

Plasma, microsomal and cytochrome P450 inhibition assessment was conducted by US Army Medical Research Institute of Chemical Defense (USAMRICD) as described in^43^. Briefly, human plasma stability was assessed by LC–MS–MS (liquid chromatography/mass spectrometry) after incubation with plasma and solid phase extraction. LLNL-02 Microsomal stability was assessed using LC–MS–MS after incubation. Cytochrome P450 inhibition was assessed using fluorescent substrates and inhibitors for 3A4, 2D6 and 2C19; CYP3A4/BQ (inh: Ketoconazole), CYP3A4/DBF (inh: Ketoconazole), CYP3A4/BFC (inh: Ketoconazole), CYP2D6/AMMC (inh: Quinidine), CYP2C19/CEC (inh: Tranylcypromine).

### In vivo pharmacokinetics

#### Animals

All experiments were conducted at the Lawrence Livermore National Laboratory (LLNL) AAALAC accredited animal care facility. The animal study protocol was reviewed and approved by the LLNL Institutional Animal Care and Use Committee prior to the initiation of the study. All experiments were performed in accordance with relevant guidelines and regulations set forth by the Guide for the Care and Use of Laboratory Animals (Eighth Ed 2011). In addition, the study was carried out in compliance with the ARRIVE guidelines”.

Male Hartley guinea pigs weighing 250–300 g with a surgically implanted jugular vein catheter were obtained from Charles River Laboratories (Wilmington, MA). Guinea pigs were housed individually in polystyrene cages containing hardwood bedding and kept on a 12 h light/dark cycle in a ventilated room maintained at 24 °C. Food and water were provided ad libitum.

#### Pharmacokinetics

LLNL-02 plasma pharmacokinetics and tissue distribution, were determined as described previously^30^. Briefly, male guinea pigs (n = 6 per dose concentration group) were administered a single IV dose of 1, 5, 10, or 20 mg/kg ^14^C-LLNL-02 dissolved in sterile saline through an implanted jugular vein catheter. Following dose administration, whole blood samples (approximately 0.3 mL) were collected from each animal via the jugular vein at 0.08, 0.25, 0.5, 1, 2, 4, 8 and 24 h post dose. Within one hour of collection the plasma was separated from the whole blood by centrifugation. The volume of plasma obtained was recorded and the sample was stored at − 80 °C until analysis. Total radiocarbon content of the plasma samples was quantified by accelerator mass spectrometry (AMS) as described previously^57^.

Pharmacokinetic parameters of LLNL-02 were calculated by non-compartmental analysis using PK Solutions software (Summit Research Services, Montrose, CO). A two-stage approach was used to independently fit the plasma concentration data from each guinea pig, and then determine the means ± standard errors. The half-life (t_1/2_) and the initial concentration observed in plasma (C_0_) were determined from the concentration-versus-time data. The area under the concentration vs. time curve (AUC) was calculated for the intervals from time zero to time t (AUC_0–t_), where t is the time of the last measurable concentration (24 h), and for time zero to infinity (AUC_0–inf_), using the linear trapezoidal method. The volume of distribution (V_d_) was determined on the basis of the AUC determination and reflects the V_d_ during the elimination phase. The clearance (CL) calculation is based on the AUC_0–inf_.

#### Biodistribution

Biodistribution of LLNL-02 was determined as described previously^30^. Briefly, guinea pigs (n = 6 per time point) were administered a single IV dose of 1, 5, 10, or 20 mg/kg ^14^C-LLNL-02 as described above. Following dose administration animals were euthanized by CO_2_ asphyxiation at 0.08, 0.25, 0.5, 1, 2, 4, 8 and 24 h post dose. Immediately following euthanasia, whole animal perfusion (heparinized (50 K U/L) PBS), was performed to ensure all the blood was removed from the tissues prior to collection. After perfusion, tissues (brain, liver, kidney, heart, spleen and lung) were excised from the carcass and rinsed twice in PBS. All tissues were then stored in glass vials (28 × 60 mm) at − 80 °C until analysis for carbon-14 content by AMS^30^.

### Statistical analysis

For in vitro assays, data are reported as the mean ± standard deviation. The PAMPA membrane is sensitive to physical manipulation, and small perturbations may lead to outlier metrics; outliers were removed using the robust nonlinear regression method in Graphpad Prism (version 6.07). P values for in vitro assay results were calculated from unpaired t tests. Two-dimensional dot plot was created using ggplot2 (version 3.0.0) in R (version 3.5.1). For the in vivo studies, values are expressed as the mean ± SE (*n* = 6), unless otherwise noted. Dose linearity was assessed using the power regression model on log-transformed data for AUC_0–t_^58^. Coefficients of determination (R^2^ values), and 95% confidence limits were determined from the power regression analysis. Results were considered linear when R^2^ was ∼1, and lower confidence limits were ≥ 0.8 and upper confidence limits were ≤ 1.25^59^.

### Associated content

The human homology model labelled HumanModel will be made available upon publication. Compound structures with labels and smiles strings are supplied as supporting information.

### Disclaimer

This document was prepared as an account of work sponsored by an agency of the United States government. Neither the United States government nor Lawrence Livermore National Security, LLC, nor any of their employees makes any warranty, expressed or implied, or assumes any legal liability or responsibility for the accuracy, completeness, or usefulness of any information, apparatus, product, or process disclosed, or represents that its use would not infringe privately owned rights. Reference herein to any specific commercial product, process, or service by trade name, trademark, manufacturer, or otherwise does not necessarily constitute or imply its endorsement, recommendation, or favoring by the United States government or Lawrence Livermore National Security, LLC. The views and opinions of authors expressed herein do not necessarily state or reflect those of the United States government or Lawrence Livermore National Security, LLC, and shall not be used for advertising or product endorsement purposes.

## Supplementary Information

Supplementary Information 1.

Supplementary Information 2.

Supplementary Information 3.

## Abbreviations

AChE: Acetylcholinesterase
2-PAM: 2-Methyl pralidoxime
CNS: Central nervous system
PNS: Peripheral nervous system
OPNAs: Organophosphate nerve agents
NIMP: Nitrophenyl isopropyl methylphosphonate
SAR: Structure activity relationship
UPLC: Ultraperformance liquid chromatography
PAMPA: Parallel artificial membrane permeability assay
MDR1 and pGp: Multidrug resistance protein 1
MDCK: Madin-darbin canine kidney cells
NFR: Net flux ratio
HBSS: Hanks balanced salt solution
PVDF: Polyvinylidene fluoride
HCME: Human cerebral microvascular endothelial cells
FBS: Fetal bovine serum
AUC: Area under the curve
