# Supplementary material for: Development of a CNS-permeable reactivator for nerve agent exposure: an iterative, multi-disciplinary approach
Source: Sci Rep. 2021 Jul 30;11:15567. doi: 10.1038/s41598-021-94963-2 (PMC8324913; doi:10.1038/s41598-021-94963-2)
Supplement: Supplementary file 1 — Supplementary Information 1. [file 41598_2021_94963_MOESM1_ESM.docx]

**Supporting Information**

**Part 1**

Development of a CNS-permeable Reactivator for Nerve Agent Exposure: An Iterative, Multi-Disciplinary Approach

Brian J. Bennion^a^, Michael ­A. Malfatti^a^, Nicholas A. Be^a^, Heather A. Enright^a^, Saphon Hok^b,c^, C. Linn Cadieux^d^, Timothy S. Carpenter^a^, Victoria Lao^a^, Edward A. Kuhn^a^, M. Windy. McNerney^a^, Felice C. Lightstone^a^, Tuan H. Nguyen^e^, Carlos A. Valdez^b,c,*^

**Table of Contents**

**Content** **Page**

Table of contents S1

Figure S1 “Integrated Workflow” S2

Figure S2 Plasma Dose vs AUC S3

Figure S3. LLNL compounds predicted membrane permeabilities S4

Figure S4. LLNL-02 Synthetic Route S5

Table S1 LLNL-02 Plasma and P450 Stability S7

Specific Compound Syntheses S7

Figure S5. LLNL-05 Synthetic Route S15


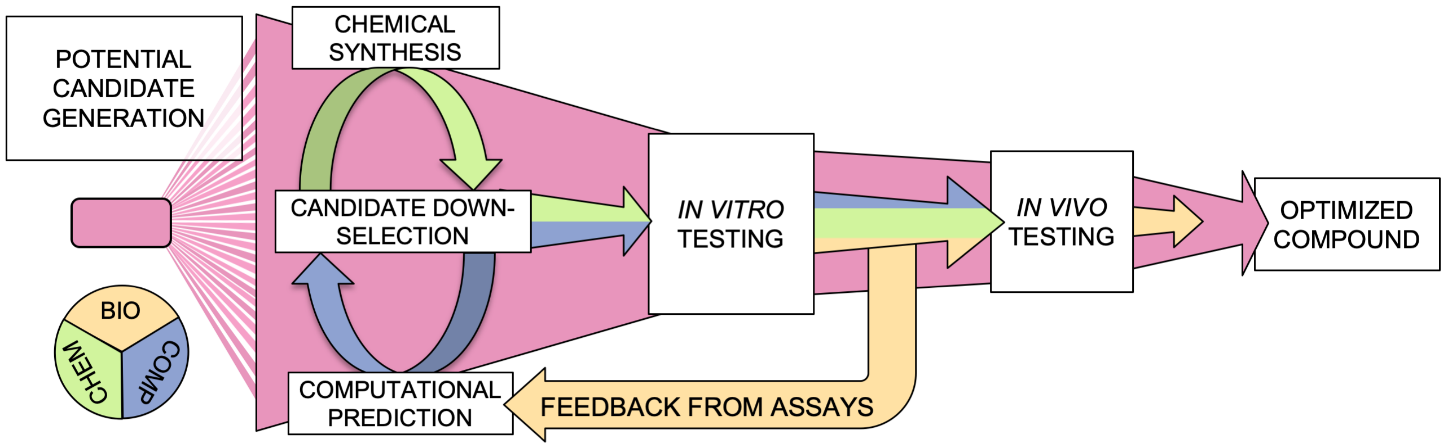


**Figure S1.**  Our project flowchart illustrates the integrated, iterative nature of our approach. Both synthetic and computational methods are cycled through to refine potential candidates. These candidates are tested *in vitro* and the results from these assays are used to refine our computational predictive models. Finally, the best candidates are down-selected from the *in vitro* assays and put forward for full *in vivo* testing to produce an optimized compound.

**Figure S2**. Power regression analysis of the log plasma dose of LLNL-02 versus the log plasma AUC_0-t_ following single intravenous administrations of 1.0, 5.0, 10 or 20 mg/kg ^14^C-LLNL-02 to male guinea pigs; R^2^ = 0.9229.


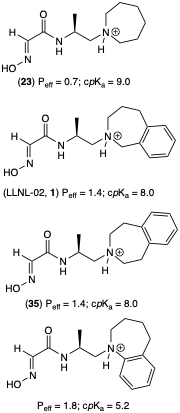


**Figure S3.**  LLNL compounds with predicted membrane permeabilities and calculated *p*K_a_ values. The addition of a benzo moiety near the tertiary nitrogen atom on the azepine ring increases the predicted membrane permeability while reducing the nitrogen atom *p*K_a_. When combined with an oxime *p*K_a_ of ~8, the neutral azepine nitrogen increases the concentration of uncharged compound thereby increasing the probability of passive diffusion through the lipid membrane. All cpKa calculations used ChemDraw16.

**Figure S4.** Synthetic route to LLNL-02 (**1**).

Solvents used during the syntheses were removed by using a Büchi rotary evaporator R-200 equipped with a Büchi heating bath B-490 and coupled to a KNF Laboport Neuberger UN820 vacuum pump. Analytical thin layer chromatography (TLC) was conducted on Agela Technologies silica gel glass plates coupled with detection ceric ammonium molybdate (CAM), exposure to iodine vapor and/or UV light (l = 254 nm). Purification of all synthetic intermediates and the final oximes for their *in vitro*/*in vivo* testing was accomplished using a Biotage Purification System using silica gel cartridges coupled to a UV detection system (l = 245, 365 nm). ^1^H NMR (600 MHz) and ^13^C NMR (151 MHz) were recorded in CDCl_3_ and DMSO*_d6_*. Spectra were obtained using a Bruker Avance III 600 MHz instrument equipped with a Bruker TCI 5 mm cryoprobe (Bruker Biospin, Billerica, MA) at 30.0 ± 0.1 °C. NMR data is reported as follows: chemical shift (d) (parts per million, ppm); multiplicity: s (singlet), d (doublet), t (triplet), q (quartet) and br (broad); coupling constants (*J*) are given in Hertz (Hz). ^1^H NMR chemical shifts are calibrated with respect to residual chloroform in CDCl_3_ centered at 7.26 ppm, CD_3_OD centered at 3.31 ppm or DMSO*_d6_* centered at 2.54 ppm, whereas for ^13^C NMR, the center peak for CDCl_3_, centered at 77.0 ppm, CD_3_OD centered at 49.0 ppm or DMSO*_d6_* centered at 40.5 ppm, were used for the calibration. In some ^13^C spectra the specific assignment of a given carbon to a signal was done with the aid of ^13^C DEPT-135. HRMS analyses were obtained at the Forensic Science Center at the Lawrence Livermore National Laboratory using Chemical Ionization (CI). Deuteriochloroform (CDCl_3_), methanol-_d4_ and DMSO-_d6_ were purchased from Cambridge Isotope Laboratories (Tewksbury, MA). 1-(Azepan-1-yl)propan-2-amine, 1-(piperidin-1-yl)propan-2-amine, (2-aminopropyl)dimethylamine, (2-aminopropyl)diethylamine, a-methyl-1-pyrrolidineethanamine, a,4-dimethyl-1-piperidineethanamine, were purchased from Enamine Building Blocks (Cincinnati, OH). 2(S)-2-(Boc-amino)propanal (Boc-ala-aldehyde), 4-Piperidinopiperidine, were purchased from Combiblocks (San Diego, CA.). Aminocyclopropane, aminocyclobutane, 1-aminobutane, benzylamine, homopiperazine and (*S*)-(+)-α-Methoxy-α-(trifluoromethyl)phenylacetyl chloride (Mosher’s acyl chloride) were purchased from Sigma-Aldrich (St Louis, MO). Ethyl glyoxylate oxime and all RS-based compounds were synthesized according to Sit ^14^ and obtained as a pale-yellow liquid that was stored at 4 °C and only taken out when used. Elemental analyses were conducted at Galbraith Laboratories (Knoxville, TN).

**(*S*)-1-(1,3,4,5-tetrahydro-2*H*-benzo[*c*]azepin-2-yl)propan-2-amine** (**5**). Boc-protected benzoazepine **4** (3.3 g, 10.9 mmol) was dissolved in diethyl ether (100 mL) in a 250 mL round bottom flask equipped with a stir bar. To this solution, 2 M HCl/Et_2_O (22 mL, 4 equiv. to **4**) was added dropwise using an addition funnel over 5 minutes at ambient temperature. The resulting mixture which became a white suspension was stirred at ambient temperature overnight. The mixture was filtered, and the combined filtrates evaporated to yield a white solid which was combined with the initially collected white precipitate. The solid was dissolved with 1 N NaOH (100 mL) in a 250 mL Erlenmeyer flask and then transferred to a separatory funnel where it was extracted with diethyl ether (4 x 50 mL). The combined organics were dried over anhydrous sodium sulfate and evaporated *in vacuo* to give **5** as an amber oil of high purity by NMR (2.13 g, 96%). R*_f_* (1:9 MeOH/DCM): 0.45 (long streak, UV + I_2_ vapor positive); ^1^H NMR (CDCl_3_, 600 MHz) δ 7.15-7.07 (m, 4H), 3.90 (br s, 3H), 3.14-3.12 (m, 1H), 3.10-3.08 (m, 1H), 3.04-3.00 (m, 1H), 2.91-2.89 (m, 2H), 2.32 (dd, *J* = 12.6, 3.6 Hz, 1H), 1.99 (app dd, *J* = 9.6, 3.0 Hz, 1H), 1.71-1.68 (m, 3H), 0.95 (d, *J* = 6.6 Hz, 3H, CH_3_); ^13^C NMR (151 MHz) δ 142.9, 139.6, 129.6, 128.8, 127.0, 125.8, 61.4, 59.7, 59.4, 43.8, 36.2, 25.1, 21.3; Anal. Calcd. for C_13_H_20_N_2_: C, 76.42; H, 9.87; N, 13.71; Found: C, 76.23; H, 9.98; N, 13.59; HRMS (CI) *m/z* calcd for C_13_H_20_N_2_ [M^+^]: 204.1626; found 204.1618. At this amine stage, we needed to check that the chiral center had not inadvertently epimerized when performing the reductive amination step. To this end, we reacted **5** with (*S*)-(+)-α-Methoxy-α-(trifluoromethyl)phenylacetyl chloride (Mosher’s acyl chloride) and found that the doublet originating from the methyl group shifted from δ = 0.95 ppm to δ = 1.18 ppm upon amide formation. Furthermore, this was the only signal arising from the reaction, thus proving that there is one single (S) enantiomer in **5**.

**Table S1.** *In vitro* evaluation of LLNL-02 stability and cytochrome P450 inhibition

|  | **Plasma stability**  **(percent remaining)^a^** | **Microsomal stability**  **t_1/2_ (min)^b^** | **P450 inhibition^c^**  **Enzyme/substrate (µM)** | | | | |
| --- | --- | --- | --- | --- | --- | --- | --- |
|  |  |  | **CYP3A4/ BQ** | **CYP3A4/ DBF** | **CYP3A4/ BFC** | **CYP2D6**  **/AMMC** | **CYP2C19**  **/CEC** |
| LLNL-02 | > 90.0 | > 60.0 | >10 | >10 | 7.4 | 1.5 | >10 |

^a^Plasma stability (percent remaining after 1 hour) was determined following incubation for 1 hour at 37 °C; > 85% (good), 20-85% (moderate), <20% (poor).

^b^Substrate depletion experiments were performed by incubating LLNL-02 with human liver microsomes for 1 hour at 37 °C; t_1/2_ >30 min (good), >15 min and <30 min (Moderate), <15 min (poor).

**^c^**P450 Enzyme Inhibition of 3A4, 2D6, 2C19, using Fluorescent Substrates. The IC50 value for each drug was determined from the net fluorescent signal from incubation at 37 °C for a set time with an active cytochrome P450 enzyme and a fluorescent probe substrate.

High IC50 (> 10 µM) = Poor Inhibitor

Mid IC50 (1 to 10 µM) = Moderate Inhibitor

Low IC50 (< 1 µM) = Good

***tert*-butyl (*E*)-(2-(2-(hydroxyimino)acetamido)ethyl)carbamate** (**7**) **[CV9-095]**. *tert*-butyl (2-aminoethyl)carbamate (0.5 g, 3.1 mmol) and ethyl glyoxylate oxime (**6**) (0.33 g, 2.8 mmol, 0.9 equiv. to amine) were reacted using the general synthetic procedure to give **7** as a white solid that precipitated out of the hot EtOH solution upon overnight stirring (0.27 g, 41%). R*_f_* (1:1 EtOAc/hexanes): 0.34; ^1^H NMR (DMSO-_d6_, 600 MHz) δ 11.91 (s, 1H), 8.11 (t, *J* = 5.4 Hz, 1H), 7.39 (s, 1H), 6.84 (t, *J* = 5.4 Hz, 1H), 3.15 (q, *J* = 6.0 Hz, 2H), 2.99 (q, *J* = 6.0 Hz, 2H), 1.34 (s, 9H); ^13^C NMR (DMSO-_d6_, 151 MHz) δ 162.4, 156.0, 144.1, 78.1, 39.1, 28.6; Anal. Calcd. for C_9_H_17_N_3_O_4_: C, 46.75; H, 7.41; N, 18.17; Found: C, 46.82; H, 7.24; N, 18.02; HRMS (CI) *m/z* calcd for C_9_H_17_N_3_O_4_ [M^+^]: 231.1219; found 231.1214.

***tert*-butyl (*R*,*E*)-3-(2-(hydroxyimino)acetamido)pyrrolidine-1-carboxylate** (**8**) **[CV9-087]**. *tert*-butyl (*R*)-3-aminopyrrolidine-1-carboxylate (0.22 g, 1.18 mmol) and ethyl glyoxylate oxime (**6**) (0.12 g, 1.06 mmol, 0.9 equiv. to amine) were reacted using the general synthetic procedure to give **8** as a white solid (89 mg, 33%) after purification by flash column chromatography (2:8 🡪 7:3 EtOAc/hexanes). R*_f_* (1:1 EtOAc/hexanes): 0.30; ^1^H NMR (CDCl_3_, 600 MHz) δ 10.98 (s, 1H), 7.44 (s, 1H), 6.83 (d, *J* = 7.6 Hz, 1H), 4.48-4.44 (m, 1H), 3.63-3.59 (m, 1H), 3.43-3.38 (m, 2H), 3.26-3.20 (m, 1H), 2.16-2.11 (m, 1H), 1.91-1.87 (m, 1H), 1.42 (s, 9H), ^13^C NMR (CDCl_3_, 151 MHz) δ 162.8, 155.4, 144.9, 78.8, 59.6, 48.1, 47.6, 33.5, 28.7; Anal. Calcd. for C_11_H_19_N_3_O_4_: C, 51.35; H, 7.44; N, 16.33; Found: C, 51.40; H, 7.49; N, 16.21; HRMS (CI) *m/z* calcd for C_11_H_19_N_3_O_4_ [M^+^]: 257.1376; found 257.1368.

***tert*-butyl (*E*)-3-(2-(hydroxyimino)acetamido)azetidine-1-carboxylate** (**9**) **[CV9-086].** *tert*-butyl 3-aminoazetidine-1-carboxylate (0.5 g, 2.9 mmol) and ethyl glyoxylate oxime (**6**) (0.3 g, 2.6 mmol, 0.9 equiv. to amine) were reacted using the general synthetic procedure to give **9** as a yellow oil (195 mg, 31%) after purification by flash column chromatography (3:7 🡪 7:3 EtOAc/hexanes). R*_f_* (1:1 EtOAc/hexanes): 0.28; ^1^H NMR (DMSO-_d6_, 600 MHz) δ 12.0 (br s, 1H), 8.77 (d, *J* = 6.0 Hz, 1H), 7.42 (s, 1H), 4.52-4.48 (m, 1H), 4.03 (br s, 2H), 3.78 (br s, 2H), 1.37 (s, 9H); ^13^C NMR (DMSO-_d6_, 151 MHz) δ 162.2, 156.0, 143.8, 79.1, 38.9, 28.5; Anal. Calcd. for C_10_H_17_N_3_O_4_: C, 49.37; H, 7.04; N, 17.27; Found: C, 49.28; H, 6.84; N, 17.13; HRMS (CI) *m/z* calcd for C_10_H_17_N_3_O_4_ [M^+^]: 243.1219; found 243.1215.

***tert*-butyl (*E*)-3-(2-(hydroxyimino)acetamido)piperidine-1-carboxylate** (**10**) **[CV9-085]**. *tert*-butyl 3-aminopiperidine-1-carboxylate (0.52 g, 2.6 mmol) and ethyl glyoxylate oxime (**6**) (0.27 g, 2.34 mmol, 0.9 equiv. to amine) were reacted using the general synthetic procedure to give **10** as an off-white solid (222 mg, 35%) after purification by flash column chromatography (3:7 🡪 7:3 EtOAc/hexanes). R*_f_* (1:1 EtOAc/hexanes): 0.44; ^1^H NMR (DMSO-_d6_, 600 MHz) δ 11.92 (br s, 1H), 8.00 (d, *J* = 6.0 Hz, 1H), 7.48 (s, 1H), 3.67-3.54 (m, 4H), 2.98-2.68 (m, 2H), 1.79-1.77 (m, 1H), 1.65-1.63 (m, 1H), 1.49-1.47 (m, 1H), 1.38 (s, 9H), ^13^C NMR (DMSO-_d6_, 151 MHz) δ 161.7, 154.4, 144.1, 79.2, 48.1, 45.6, 44.2, 29.8, 28.5, 23.3; Anal. Calcd. for C_12_H_21_N_3_O_4_: C, 53.12; H, 7.80; N, 15.49; Found: C, 53.22; H, 7.66; N, 15.43; HRMS (CI) *m/z* calcd for C_12_H_21_N_3_O_4_ [M^+^]: 271.1532; found 271.1531.

**(*E*)-*N*-butyl-2-(hydroxyimino)acetamide** (**11**) **[CV9-116]**. 1-aminobutane (0.7 mL, 0.52 g, 7.1 mmol) and ethyl glyoxylate oxime (**6**) (0.75 g, 6.38 mmol, 0.9 equiv. to amine) were reacted using the general synthetic procedure to give **11** as a pale yellow solid (514 mg, 56%) after purification by flash column chromatography (3:7 🡪 7:3 EtOAc/hexanes). R*_f_* (1:1 EtOAc/hexanes): 0.60 (long streak); ^1^H NMR (DMSO-_d6_, 600 MHz) δ 11.90 (s, 1H), 8.10 (s, 1H), 7.43 (s, 1H), 3.13 (t, *J* = 6.7 Hz, 2H), 1.42 (quint, *J* = 7.4 Hz, 2H), 1.26 (sext, *J* = 7.6 Hz, 2H), 0.87 (d, *J* = 7.6 Hz, 3H); ^13^C NMR (DMSO-_d6_, 151 MHz) 162.1, 144.2, 38.5, 31.5, 19.9, 14.0; Anal. Calcd. for C_6_H_12_N_2_O_2_: C, 49.99; H, 8.39; N, 19.43; Found: C, 50.21; H, 8.18; N, 19.57; HRMS (CI) *m/z* calcd for C_6_H_12_N_2_O_2_ [M^+^]: 144.0899; found 144.0893.

**(*E*)-*N*-cyclopentyl-2-(hydroxyimino)acetamide** (**12**) **[CV9-118]**. Aminocyclopentane (0.6 g, 7.05 mmol) and ethyl glyoxylate oxime (**6**) (0.74 g, 6.35 mmol, 0.9 equiv. to amine) were reacted using the general synthetic procedure to give **12** as a white solid (446 mg, 45%) after purification by flash column chromatography (2:8 🡪 1:1 EtOAc/hexanes). R*_f_* (1:1 EtOAc/hexanes): 0.65; ^1^H NMR (CDCl_3_, 600 MHz) δ 9.56 (s, 1H), 7.50 (s, 1H), 6.57 (d, *J* = 6.6 Hz, 1H), 4.27 (sext, *J* = 7.3 Hz, 1H), 2.04-2.01 (m, 2H), 1.73-1.70 (m, 2H), 1.65-1.63 (m, 2H), 1.49-1.44 (m, 2H); ^13^C NMR (CDCl_3_, 151 MHz) δ 162.0, 144.4, 50.9, 32.9, 23.7; Anal. Calcd. for C_7_H_12_N_2_O_2_: C, 53.83; H, 7.74; N, 17.94; Found: C, 54.01; H, 7.79; N, 18.10; HRMS (CI) *m/z* calcd for C_7_H_12_N_2_O_2_ [M^+^]: 156.0899; found 156.0896.

**(*E*)-*N*-benzyl-2-(hydroxyimino)acetamide** (**13**) **[CV9-119]**. Benzylamine (1.0 g, 9.3 mmol) and ethyl glyoxylate oxime (**6**) (0.98 g, 8.4 mmol, 0.9 equiv. to amine) were reacted using the general synthetic procedure to give **13** as a yellow oil (568 mg, 38%) after purification by flash column chromatography (2:8 🡪 7:3 EtOAc/hexanes). R*_f_* (1:1 EtOAc/hexanes): 0.66; ^1^H NMR (DMSO-_d6_, 600 MHz) δ 11.68 (br s, 1H), 8.44 (br s, 1H), 7.85 (s, 1H), 7.34-7.21 (m, 5H), 4.38 (s, 2H); ^13^C NMR (DMSO-_d6_, 151 MHz) δ 161.1, 144.8, 143.0, 128.8, 127.7, 126.9, 46.3; Anal. Calcd. for C_9_H_10_N_2_O_2_: C, 60.66; H, 5.66; N, 15.72; Found: C, 60.78; H, 5.80; N, 15.77; HRMS (CI) *m/z* calcd for C_9_H_10_N_2_O_2_ [M^+^]: 178.0742; found 178.0733.

**(1*E*,1'*E*)-2,2'-(1,4-diazepane-1,4-diyl)bis(2-oxoacetaldehyde) dioxime** (**14**) **[CV9-081]**. Homopiperazine (0.7 g, 6.98 mmol) and ethyl glyoxylate oxime (**6**) (1.55 g, 13.3 mmol, 1.9 equiv. to amine) were reacted using the general synthetic procedure to give **14** as a yellow oil (354 mg, 21%) after purification by flash column chromatography (1:1 EtOAc/hexanes 🡪 EtOAc). R*_f_* (7:3 EtOAc/hexanes): 0.35; ^1^H NMR (DMSO-_d6_, 600 MHz) δ 11.49 (s, 2H, 2 x N-OH), 7.36 (br s, 2H), 3.51-3.42 (m, 4H), 3.12-3.07 (m, 4H), 1.65-1.61 (m, 2H); ^13^C NMR (DMSO-_d6_, 151 MHz) δ 166.6, 148.3, 48.1, 44.2, 33.8; Anal. Calcd. for C_9_H_14_N_4_O_4_: C, 44.63; H, 5.83; N, 23.13; Found: C, 44.51; H, 5.65; N, 23.06; HRMS (CI) *m/z* calcd for C_9_H_14_N_4_O_4_ [M^+^]: 242.1015; found 242.1009.

**(*E*)-*N*-cyclopropyl-2-(hydroxyimino)acetamide** (**15**) **[CV10-122-A]**. Aminocyclopropane (0.2 g, 3.5 mmol) and ethyl glyoxylate oxime (**6**) (0.37 g, 3.2 mmol, 0.9 equiv. to amine) were reacted using the general synthetic procedure to give **15** as a pale yellow solid (131 mg, 32%) after purification by flash column chromatography (2:8 🡪 1:1 EtOAc/hexanes). R*_f_* (3:7 EtOAc/hexanes): 0.30; ^1^H NMR (CDCl_3_, 600 MHz) δ 8.33 (br s, 1H), 7.47 (s, 1H), 6.60 (br s, 1H), 2.78-2.74 (m, 1H), 0.83-0.80 (m, 2H), 0.59-0.56 (m, 2H); ^13^C NMR (CDCl_3_, 151 MHz) δ 163.3, 144.6, 22.2, 6.6; Anal. Calcd. for C_5_H_8_N_2_O_2_: C, 46.87; H, 6.29; N, 21.86; Found: C, 46.80; H, 6.22; N, 21.73; HRMS (CI) *m/z* calcd for C_5_H_8_N_2_O_2_ [M^+^]: 128.0586; found 128.0585.

**(*E*)-*N*-cyclobutyl-2-(hydroxyimino)acetamide** (**16**) **[CV10-122-B]**. Aminocyclobutane (0.3 g, 4.2 mmol) and ethyl glyoxylate oxime (**6**) (0.45 g, 3.8 mmol, 0.9 equiv. to amine) were reacted using the general synthetic procedure to give **16** as a pale yellow solid (151 mg, 28%) after purification by flash column chromatography (2:8 🡪 1:1 EtOAc/hexanes). R*_f_* (3:7 EtOAc/hexanes): 0.35; ^1^H NMR (CD_3_OD, 600 MHz) δ 7.40 (s, 1H), 4.39-4.34 (m, 1H), 2.31-2.27 (m, 2H), 2.03-1.99 (m, 2H), 1.76-1.71 (m, 2H), ^13^C NMR (CD_3_OD, 151 MHz) δ 163.5, 143.8, 45.5, 31.1, 15.8; Anal. Calcd. for C_6_H_10_N_2_O_2_: C, 50.69; H, 7.09; N, 19.71; Found: C, 50.72; H, 7.02; N, 19.66; HRMS (CI) *m/z* calcd for C_6_H_10_N_2_O_2_[M^+^]: 142.0742; found 142.0742.

***tert*-Butyl (E)-(1-(2-(hydroxyimino)acetyl)pyrrolidin-3-yl)carbamate** (**17**) **[CV10-138-B]**. *tert*-Butyl pyrrolidin-3-ylcarbamate (0.5 g, 2.7 mmol) and ethyl glyoxylate oxime (**6**) (0.28 g, 2.4 mmol, 0.9 equiv. to amine) were reacted using the general synthetic procedure to give **17** as a white solid (137 mg, 22%) after purification by flash column chromatography (3:7 🡪 7:3 EtOAc/hexanes). R*_f_* (1:1 EtOAc/hexanes): 0.55; ^1^H NMR (DMSO-_d6_, 600 MHz) δ 11.81 (s, 1H), 8.34 (s, 1H), 6.81 (br s, 1H), 3.81 (br s, 1H), 2.81-2.78 (m, 2H), 2.66-2.64 (m, 1H), 2.48-2.47 (m, 1H), 1.84-179 (m, 1H), 1.46-1.44 (m, 1H), 1.36 (s, 9H), ^13^C NMR (DMSO-_d6_, 151 MHz) δ 161.1, 156.0, 145.9, 78.3, 53.7, 52.0, 45.7, 33.3, 28.9; Anal. Calcd. for C_11_H_19_N_3_O_4_: C, 51.35; H, 7.44; N, 16.33; Found: C, 51.21; H, 7.43; N, 16.23; HRMS (CI) *m/z* calcd for C_11_H_19_N_3_O_4_ [M^+^]: 257.1376; found 257.1375.

***(E*)-2-oxo-2-(4-(2-(thiophen-2-yl)ethyl)piperazin-1-yl)acetaldehyde oxime** (**18**) **[CV9-165-E]**. 1-(2-(thiophen-2-yl)ethyl)piperazine (0.4 g, 2.0 mmol) and ethyl glyoxylate oxime (**6**) (0.2 g, 1.84 mmol, 0.9 equiv. to amine) were reacted using the general synthetic procedure to give **18** as an off-white solid (177 mg, 36%) after purification by flash column chromatography (3:7 🡪 7:3 EtOAc/hexanes). R*_f_* (1:1 EtOAc/hexanes): 0.64; ^1^H NMR (DMSO-_d6_, 600 MHz) δ 11.76 (s, 1H), 7.77 (s, 1H), 7.28 (dd, *J* = 4.8, 1.2 Hz, 1H), 6.91 (dd, *J* = 5.4, 3.6 Hz, 1H), 6.87-6.86 (m, 1H), 3.58 (t, *J* = 4.8 Hz, 2H), 3.51 (t, *J* = 4.8 Hz, 2H), 2.94 (t, *J* = 7.2 Hz, 2H), 2.56 (t, *J* = 7.2 Hz, 2H), 2.44-2.42 (m, 4H); ^13^C NMR (DMSO-_d6_, 151 MHz) δ 161.2, 145.6, 138.8, 128.3, 126.8, 124.3, 56.6, 56.3, 54.8, 50.2; Anal. Calcd. for C_12_H_17_N_3_O_2_S: C, 53.91; H, 6.41; N, 15.72; Found: C, 53.80; H, 6.24; N, 15.58; HRMS (CI) *m/z* calcd for C_12_H_17_N_3_O_2_S [M^+^]: 267.1041; found 267.1037.

**(*E*)-2-(4-(4-chlorobenzyl)piperazin-1-yl)-2-oxoacetaldehyde oxime** (**19**) **[CV10-042-B]**. 1-(4-Chlorobenzyl)piperazine (0.7 g, 3.3 mmol) and ethyl glyoxylate oxime (**6**) (0.35 g, 3.0 mmol, 0.9 equiv. to amine) were reacted using the general synthetic procedure to give **19** as an off-white solid (337 mg, 40%) after purification by flash column chromatography (2:8 🡪 8:2 EtOAc/hexanes). R*_f_* (1:1 EtOAc/hexanes): 0.68; ^1^H NMR (DMSO-_d6_, 600 MHz) δ 11.80 (s, 1H), 7.80 (s, 1H), 7.36 (d, *J* = 8.3 Hz, 2H), 7.31 (d, *J* = 8.3 Hz, 2H), 3.41 (s, 2H), 3.09-3.07 (m, 4H), 2.61-2.58 (m, 4H); ^13^C NMR (DMSO-_d6_, 151 MHz) δ 161.3, 144.9, 138.4, 132.5, 131.8, 129.2, 66.8, 55.0, 46.6; Anal. Calcd. for C_13_H_16_ClN_3_O_2_: C, 55.42; H, 5.72; N, 14.91; Found: C, 55.56; H, 5.86; N, 15.01; HRMS (CI) *m/z* calcd for C_13_H_16_ClN_3_O_2_ [M^+^]: 281.0931; found 281.0929.

**(*E*)-2-(4-(cyclopentylmethyl)piperazin-1-yl)-2-oxoacetaldehyde oxime** (**20**) **[CV10-042-C]**. 1-(Cyclopentylmethyl)piperazine (0.3 g, 1.8 mmol) and ethyl glyoxylate oxime (**6**) (0.19 g, 1.6 mmol, 0.9 equiv. to amine) were reacted using the general synthetic procedure to give **20** as a white solid (160 mg, 42%) after purification by flash column chromatography (3:7 🡪 7:3 EtOAc/hexanes). R*_f_* (1:1 EtOAc/hexanes): 0.68; ^1^H NMR (DMSO-_d6_, 600 MHz) δ 11.67 (s, 1H), 7.77 (s, 1H), 3.11-3.06 (m, 4H), 2.24-2.20 (m, 4H), 2.10-2.07 (m, 2H), 2.01-1.90 (m, 1H), 1.65-1.63 (m, 2H), 1.49-1.43 (m, 4H), 1.14-1.09 (m, 2H); ^13^C NMR (DMSO-_d6_, 151 MHz) δ 161.1, 144.8, 66.3, 56.1, 47.1, 37.8, 32.3, 26.2; Anal. Calcd. for C_12_H_21_N_3_O_2_: C, 60.23; H, 8.85; N, 17.56; Found: C, 60.18; H, 8.88; N, 17.59; HRMS (CI) *m/z*calcd for C_12_H_21_N_3_O_2_ [M^+^]: 239.1634; found 239.1628.

**(*E*)-2-(4-(3-chlorobenzyl)piperazin-1-yl)-2-oxoacetaldehyde oxime** (**21**) **[CV10-049-A]**. 1-(3-Chlorobenzyl)piperazine (0.4 g, 1.9 mmol) and ethyl glyoxylate oxime (**6**) (0.2 g, 1.7 mmol, 0.9 equiv. to amine) were reacted using the general synthetic procedure to give **21** as an off-white solid (158 mg, 33%) after purification by flash column chromatography (2:8 🡪 8:2 EtOAc/hexanes). R*_f_* (1:1 EtOAc/hexanes): 0.70; ^1^H NMR (DMSO-_d6_, 600 MHz) δ 11.77 (s, 1H), 7.81 (s, 1H), 7.32-7.31 (m, 2H), 7.27-7.26 (m, 1H), 7.22-7.21 (m, 1H), 3.39 (s, 2H), 3.14-3.13 (m, 4H), 2.26-2.23 (m, 4H); ^13^C NMR (DMSO-_d6_, 151 MHz) δ 161.4, 144.8, 137.5, 129.4, 126.5, 125.0, 124.0, 123.3, 58.5, 50.4, 46.3 ; Anal. Calcd. for C_13_H_16_ClN_3_O_2_: C, 55.42; H, 5.72; N, 14.91; Found: C, 55.60; H, 5.79; N, 14.99; HRMS (CI) *m/z* calcd for C_13_H_16_ClN_3_O_2_ [M^+^]: 281.0931; found 281.0930.

**(*E*)-2-(4-isobutylpiperazin-1-yl)-2-oxoacetaldehyde oxime** (**22**) **[CV10-049-B]**. 1-Isobutylpiperazine (0.2 g, 1.41 mmol) and ethyl glyoxylate oxime (**6**) (0.15 g, 1.27 mmol, 0.9 equiv. to amine) were reacted using the general synthetic procedure to give **22** as a white solid (103 mg, 38%) after purification by flash column chromatography (3:7 🡪 8:2 EtOAc/hexanes). R*_f_* (1:1 EtOAc/hexanes): 0.65; ^1^H NMR (CDCl_3_, 600 MHz) δ 8.22 (s, 1H), 7.78 (s, 1H), 4.04 (br s, 4H), 3.67 (br s, 4H), 2.14-2.12 (m, 2H), 1.81-1.76 (m, 1H), 0.90 (d, *J* = 6.6 Hz, 6H); ^13^C NMR (CDCl_3_, 151 MHz) δ160.7, 144.5, 67.3, 57.2, 49.3, 45.4, 20.8; Anal. Calcd. for C_10_H_19_N_3_O_2_: C, 56.32; H, 8.98; N, 19.70; Found: C, 56.45; H, 9.03; N, 19.81; HRMS (CI) *m/z* calcd for C_10_H_19_N_3_O_2_ [M^+^]: 213.1477; found 213.1474.

**(*E*)-*N*-(1-(azepan-1-yl)propan-2-yl)-2-(hydroxyimino)acetamide** (**23**) **[CV10-155]**. 1-(Azepan-1-yl)propan-2-amine (0.5 g, 3.2 mmol) and ethyl glyoxylate oxime (**6**) (0.34 g, 2.9 mmol, 0.9 equiv. to amine) were reacted using the general synthetic procedure to give **23** as an off-white solid (296 mg, 45%) after purification by flash column chromatography (3:7 🡪 8:2 EtOAc/hexanes). R*_f_* (1:1 EtOAc/hexanes): 0.36; ^1^H NMR (CDCl_3_, 600 MHz) δ 8.03 (br s, 1H), 7.51 (s, 1H), 4.42 (br s, 1H), 3.37-3.29 (m, 1H), 3.17-3.12 (m, 3H), 2.92-2.90 (m, 1H), 1.84-1.81 (br s, 4H), 1.66-1.63 (br m, 6H), 1.26 (d, *J* = 6.6 Hz, 3H); ^13^C NMR (CDCl_3_, 151 MHz) δ 163.1, 143.5, 60.7, 55.1, 41.5, 27.1, 24.2, 19.4; Anal. Calcd. for C_11_H_21_N_3_O_2_: C, 58.12; H, 9.31; N, 18.49; Found: C, 58.01; H, 9.18; N, 18.39; HRMS (CI) *m/z* calcd for C_11_H_21_N_3_O_2_[M^+^]: 227.1634; found 227.1632.

**(*E*)-*N*-(1-(dimethylamino)propan-2-yl)-2-(hydroxyimino)acetamide** (**24**) **[CV10-156]**. (2-Aminopropyl)dimethylamine (0.3 g, 2.9 mmol) and ethyl glyoxylate oxime (**6**) (0.31 g, 2.6 mmol, 0.9 equiv. to amine) were reacted using the general synthetic procedure to give **24** as a pale yellow solid (198 mg, 44%) after purification by flash column chromatography (3:7 🡪 8:2 EtOAc/hexanes). R*_f_* (1:1 EtOAc/hexanes): 0.33; ^1^H NMR (DMSO-_d6_, 600 MHz) δ 11.83 (s, 1H), 7.79 (d, *J* = 7.9 Hz, 1H), 7.40 (s, 1H), 3.92 (sept, *J* = 6.7 Hz, 1H), 3.15 (s, 1H), 2.29 (dd, *J* = 12.1, 8.0 Hz, 1H), 2.11 (s, 6H), 1.04 (d, *J* = 6.7 Hz, 3H); ^13^C NMR (DMSO-_d6_, 151 MHz) δ 161.5, 144.3, 64.4, 45.8, 42.9, 19.1; Anal. Calcd. for C_7_H_15_N_3_O_2_: C, 48.54; H, 8.73; N, 24.26; Found: C, 48.64; H, 8.78; N, 24.33; HRMS (CI) *m/z* calcd for C_7_H_15_N_3_O_2_ [M^+^]: 173.1164; found 173.1161.

**(*E*)-*N*-(1-(diethylamino)propan-2-yl)-2-(hydroxyimino)acetamide** (**25**) **[CV10-160-A].** (2-Aminopropyl)diethylamine (0.4 g, 3.1 mmol) and ethyl glyoxylate oxime (**6**) (0.32 g, 2.77 mmol, 0.9 equiv. to amine) were reacted using the general synthetic procedure to give **25** as a pale yellow solid (178 mg, 32%) after purification by flash column chromatography (3:7 🡪 8:2 EtOAc/hexanes). R*_f_* (1:1 EtOAc/hexanes): 0.30; ^1^H NMR (CDCl_3_, 600 MHz) δ 7.74 (br s, 1H), 7.48 (s, 1H), 4.35 (br s, 1H), 3.09 (br s, 1H), 2.92-2.67 (br m, 6H), 1.27 (d, *J* = 6.5 Hz, 3H), 1.15 (t, *J* = 6.9 Hz, 6H); ^13^C NMR (CDCl_3_, 151 MHz) δ 163.0, 143.8, 56.6, 46.6, 42.0, 19.4, 9.3; Anal. Calcd. for C_9_H_19_N_3_O_2_: C, 53.71; H, 9.52; N, 20.88; Found: C, 53.57; H, 9.44; N, 20.69; HRMS (CI) *m/z* calcd for C_9_H_19_N_3_O_2_ [M^+^]: 201.1477; found 201.1475.

**(*E*)-2-(hydroxyimino)-*N*-(1-(pyrrolidin-1-yl)butan-2-yl)acetamide** (**26**) **[CV10-160-B]**. α-ethyl-1-pyrrolidineethanamine (832 mg, 3.9 mmol) and ethyl glyoxylate oxime (**6**) (0.41 g, 3.5 mmol, 0.9 equiv. to amine) were reacted using the general synthetic procedure to give **26** as an off-white solid (278 mg, 40%) after purification by flash column chromatography (3:7 🡪 8:2 EtOAc/hexanes). R*_f_* (1:1 EtOAc/hexanes): 0.33; ^1^H NMR (CDCl_3_, 600 MHz) δ 7.73 (d, *J* = 7.5 Hz, 1H), 7.47 (s, 1H), 4.31-4.28 (m, 1H), 3.22 (br s, 1H), 3.10-2.71 (br m, 5H), 2.60-2.58 (m, 1H), 1.90 (m, 4H), 1.65-1.59 (m, 1H), 1.54-1.49 (m, 1H), 0.95 (t, *J* = 7.4 Hz, 3H); ^13^C NMR (CDCl_3_, 151 MHz) δ 163.5, 143.9, 58.5, 54.4, 48.2, 27.1, 23.2, 10.1; Anal. Calcd. for C_10_H_19_N_3_O_2_: C, 56.32; H, 8.98; N, 19.70; Found: C, 56.21; H, 8.79; N, 19.62; HRMS (CI) *m/z* calcd for C_10_H_19_N_3_O_2_ [M^+^]: 213.1477; found 213.1472.

**(*E*)-2-(hydroxyimino)-*N*-(1-(piperidin-1-yl)propan-2-yl)acetamide** (**27**) **[CV10-160-C]**. 1-(Piperidin-1-yl)propan-2-amine (0.4 g, 2.8 mmol) and ethyl glyoxylate oxime (**6**) (0.3 g, 2.5 mmol, 0.9 equiv. to amine) were reacted using the general synthetic procedure to give **27** as a cream-colored solid (245 mg, 46%) after purification by flash column chromatography (3:7 🡪 8:2 EtOAc/hexanes). R*_f_* (1:1 EtOAc/hexanes): 0.33; ^1^H NMR (CDCl_3_, 600 MHz) δ 7.75 (br s, 1H), 7.44 (s, 1H), 4.48 (br s, 1H), 2.80-2.38 (br m, 7H), 1.69-1.67 (m, 2H), 1.61-1.58 (m, 2H), 1.48-1.45 (m, 2H), 1.16 (d, *J* = 6.4 Hz, 3H); ^13^C NMR (CDCl_3_, 151 MHz) δ 163.1, 143.9, 62.3, 50.8, 40.9, 24.2, 23.4, 19.9; Anal. Calcd. for C_10_H_19_N_3_O_2_: C, 56.32; H, 8.98; N, 19.70; Found: C, 56.29; H, 8.94; N, 19.58; HRMS (CI) *m/z* calcd for C_10_H_19_N_3_O_2_[M^+^]: 213.1477; found 213.1478.

**(*E*)-2-(hydroxyimino)-*N*-(1-(4-methylpiperidin-1-yl)propan-2-yl)acetamide** (**28**) **[CV10-160-D]**. α,4-dimethyl-1-piperidineethanamine (0.55 g, 3.52 mmol) and ethyl glyoxylate oxime (**6**) (0.37 g, 3.17 mmol, 0.9 equiv. to amine) were reacted using the general synthetic procedure to give **28** as a light yellow solid (259 mg, 36%) after purification by flash column chromatography (3:7 🡪 8:2 EtOAc/hexanes). R*_f_* (1:1 EtOAc/hexanes): 0.36; ^1^H NMR (CDCl_3_, 600 MHz) δ 7.85 (d, *J* = 7.6 Hz, 1H), 7.48 (s, 1H), 4.48-4.46 (m, 1H), 3.33-3.30 (m, 1H), 3.11-3.06 (m, 2H), 2.54-2.51 (m, 1H), 2.39-2.37 (m, 1H), 2.24-2.22 (m, 1H), 1.73-1.69 (m, 3H), 1.49-1.47 (m, 2H), 1.38-1.36 (m, 1H), 1.21 (d, *J* = 6.2 Hz, 3H, Me), 0.94 (br s, 3H, Me); ^13^C NMR (CDCl_3_, 151 MHz) δ 163.0, 143.8, 61.5, 55.1, 51.8, 41.0, 32.1, 31.9, 29.8, 21.2, 19.7; Anal. Calcd. for C_11_H_21_N_3_O_2_: C, 58.12; H, 9.31; N, 18.49; Found: C, 58.04; H, 9.27; N, 18.57; HRMS (CI) *m/z* calcd for C_11_H_21_N_3_O_2_ [M^+^]: 227.1634; found 227.1631.

**(*E*)-2-(4-methyl-1,4-diazepan-1-yl)-2-oxoacetaldehyde oxime** (**29**) **[CV10-143-A]**. 1-Methyl-1,4-diazepane (0.1 g, 0.88 mmol) and ethyl glyoxylate oxime (**6**) (92 mg, 0.80 mmol, 0.9 equiv. to amine) were reacted using the general synthetic procedure to give **29** as an off-white solid (44 mg, 30%) after purification by flash column chromatography (3:7 🡪 8:2 EtOAc/hexanes). R*_f_* (1:1 EtOAc/hexanes): 0.45; ^1^H NMR (CDCl_3_, 600 MHz) δ 8.30 (br s, 1H), 7.82 (br s, 1H), 2.98-2.94 (m, 4H), 2.63-2.59 (m, 5H), 2.36 (s, 3H), 1.81-1.78 (m, 2H); ^13^C NMR (CDCl_3_, 151 MHz) δ 161.4, 144.9, 60.8, 57.2, 48.3, 47.3, 47.1 29.7; Anal. Calcd. for C_8_H_15_N_3_O_2_: C, 51.88; H, 8.16; N, 22.69; Found: C, 51.68; H, 8.12; N, 22.54; HRMS (CI) *m/z* calcd for C_8_H_15_N_3_O_2_: [M^+^]: 185.1164; found 185.1160.

**(*E*)-2-([1,4'-bipiperidin]-1'-yl)-2-oxoacetaldehyde oxime** (**30**) **[CV10-143-B].** 4-Piperidinopiperidine (0.5 g, 3.0 mmol) and ethyl glyoxylate oxime (**6**) (0.31 g, 2.67 mmol, 0.9 equiv. to amine) were reacted using the general synthetic procedure to give **30** as an off-white solid (211 mg, 33%) after purification by flash column chromatography (3:7 🡪 8:2 EtOAc/hexanes). R*_f_* (1:1 EtOAc/hexanes): 0.31; ^1^H NMR (DMSO-_d6_, 600 MHz) δ 7.88 (br s, 1H), 7.46 (s, 1H), 2.88 (t, *J* = 12.7 Hz, 1H), 2.48 (t, *J* = 12.4 Hz, 1H), 2.37-2.30 (m, 7H), 1.63-1.60 (m, 2H), 1.35-1.32 (m, 4H), 1.22-1.17 (m, 4H); ^13^C NMR (DMSO-_d6_, 151 MHz) δ 163.6, 143.3, 61.5, 49.7, 45.2, 27.9, 26.0, 24.5; Anal. Calcd. for C_12_H_21_N_3_O_2_: C, 60.23; H, 8.85; N, 17.56; Found: C, 60.14; H, 8.70; N, 17.33; HRMS (CI) *m/z* calcd for C_12_H_21_N_3_O_2_: [M^+^]: 239.1634; found 239.1631.

**(*E*)-2-(3-(dimethylamino)pyrrolidin-1-yl)-2-oxoacetaldehyde oxime** (**31**) **[CV10-143-D]**. *N*,*N*-dimethylpyrrolidin-3-amine (0.52, 4.5 mmol) and ethyl glyoxylate oxime (**6**) (0.47 g, 4.0 mmol, 0.9 equiv. to amine) were reacted using the general synthetic procedure to give **31** as a cream-colored solid (215 mg, 35%) after purification by flash column chromatography (3:7 🡪 8:2 EtOAc/hexanes). R*_f_* (1:1 EtOAc/hexanes): 0.34; ^1^H NMR (DMSO-_d6_, 600 MHz) δ 11.59 (s, 1H), 7.48 (s, 1H), 3.70-3.68 (m, 2H), 3.64-3.62 (m, 2H), 2.87 (m, 1H), 2.08 (s, 6H), 1.76-1.73 (m, 1H), 1.46-1.42 (m, 1H); ^13^C NMR (DMSO-_d6_, 151 MHz) δ 161.9, 145.7, 68.1, 51.6, 46.4, 44.8, 31.1; Anal. Calcd. for C_8_H_15_N_3_O_2_: C, 51.88; H, 8.16; N, 22.69; Found: C, 51.77; H, 8.18; N, 22.51; HRMS (CI) *m/z* calcd for C_8_H_15_N_3_O_2_: [M^+^]: 185.1164; found 185.1163.

**Figure S5.** Synthesis of benzoazepine analog **35**.

***tert*-butyl (*S*)-(1-(1,2,4,5-tetrahydro-3*H*-benzo[*d*]azepin-3-yl)propan-2-yl)carbamate** (**33**). Boc-protected alanine aldehyde (**2**) (3.0 g, 17.3 mmol) and 2,3,4,5-tetrahydro-1*H*-benzo[*d*]azepine hydrochloride (**32**) (3.47 g, 19.0 mmol) were taken up in anhydrous dichloromethane (200 mL) in a 500 mL round bottom flask equipped with a stir bar. To the suspension, sodium triacetoxyborohydride (4.0 g, 19 mmol) was added in small portions over three minutes. Once all the borohydride was added, the resulting mixture was vigorously stirred at ambient temperature overnight. The following day, methanol (50 mL) was added and the mixture was transferred to a separatory funnel where it was partitioned (H_2_O//DCM). The organic phase was washed with saturated sodium bicarbonate (NaHCO_3_/H_2_O, 3 x 50 mL), brine (NaCl/H_2_O, 2 x 50 mL), dried over anhydrous sodium sulfate and evaporated *in vacuo* at 60 ^o^C to yield a light brown solid residue that was purified by flash column chromatography to give Boc-protected benzoazepine intermediate **33** as a light brown solid (4.10 g, 78 %). R*_f_* (1:1 EtOAc/hexanes): 0.68; ^1^H NMR (CDCl_3_, 600 MHz) δ 8.01 (br s, 1H), 7.22 (d, *J* = 7.2 Hz, 2H), 7.17 (d, *J* = 7.2 Hz, 2H), 3.78 (m, 1H), 2.73-2.70 (m, 2H), 2.68-2.63 (m, 8H), 1.29 (s, 9H), 1.27 (d, *J* = 6.4 Hz, 3H), ^13^C NMR (151 MHz) δ 156.6, 143.3, 128.0, 126.1, 78.8, 66.2, 57.3, 50.4, 28.7, 28.3, 16.3; HRMS (CI) *m/z* calcd for C_18_H_28_N_2_O_2_ [M^+^]: 304.2151; found 304.2150.

**(*S*)-1-(1,2,4,5-tetrahydro-3*H*-benzo[*d*]azepin-3-yl)propan-2-amine** (**34**). Boc-protected benzoazepine **33** (2.2 g, 7.24 mmol) was dissolved in diethyl ether (30 mL) in a 100 mL round bottom flask equipped with a stir bar. To this solution, 2 M HCl/Et_2_O (14 mL) was added dropwise using an addition funnel over 5 minutes at ambient temperature. The resulting mixture which became a white suspension was stirred at ambient temperature overnight. The following day, the mixture was filtered and the combined filtrates evaporated to yield a white solid which was combined with the initially collected white precipitate. The solid was dissolved with 1 N NaOH (50 mL) in a 250 mL Erlenmeyer flask and then transferred to a separatory funnel where it was extracted with diethyl ether (4 x 50 mL). The combined organics were dried over anhydrous sodium sulfate and evaporated *in vacuo* to give **34** as a light brown oil that was taken as it is onto the next step (1.43 g, ~97%). R*_f_* (1:9 MeOH/DCM): 0.45 (long streak, I_2_ positive); HRMS (CI) *m/z* calcd for C_13_H_20_N_2_ [M^+^]: 204.1626; found 204.1623.

**(*S*,*E*)-2-(hydroxyimino)-*N*-(1-(1,2,4,5-tetrahydro-3*H*-benzo[*d*]azepin-3-yl)propan-2-yl)acetamide** (**35**) **[CV11-061-B]**. (*S*)-1-(1,2,4,5-Tetrahydro-3*H*-benzo[*d*]azepin-3-yl)propan-2-amine **34** (1.43, 7.0 mmol) and ethyl glyoxylate oxime (**6**) (1.03 g, 8.80 mmol, 1.25 equiv. to amine) were reacted using the general synthetic procedure to give **35** as a white solid (596 mg, 31%) after purification by flash column chromatography (3:7 🡪 7:3 EtOAc/hexanes). R*_f_* (1:9 MeOH/DCM): 0.70; ^1^H NMR (CDCl_3_, 600 MHz) δ 7.58 (d, *J* = 8.5 Hz, 1H), 7.51 (s, 1H), 7.15-7.08 (m, 5H), 4.47-4.40 (m, 1H), 3.02-2.98 (m, 2H), 2.92-2.85 (m, 5H), 2.79-2.64 (m, 4H), 2.54 (dd, *J* = 13.1, 4.2 Hz, 1H), 1.23 (d, *J* = 6.4 Hz, 3H); ^13^C NMR (CDCl_3_, 151 MHz) δ 162.7, 144.3, 141.2, 128.9, 126.5, 62.2, 55.1, 41.8 (*C*-H), 34.6, 19.9 (CH_3_); Anal. Calcd. for C_15_H_21_N_3_O_2_: C, 65.43; H, 7.69; N, 15.26; Found: C, 65.52; H, 7.80; N, 15.36; HRMS (CI) *m/z* calcd for C_15_H_21_N_3_O_2_: [M^+^]: 275.1634; found 275.1637.
