# Supplementary material for: Development of a CNS-permeable reactivator for nerve agent exposure: an iterative, multi-disciplinary approach
Source: Sci Rep. 2021 Jul 30;11:15567. doi: 10.1038/s41598-021-94963-2 (PMC8324913; doi:10.1038/s41598-021-94963-2)
Supplement: Supplementary file 2 — Supplementary Information 2. [file 41598_2021_94963_MOESM2_ESM.pdf]

Supporting Information
Part2

Development of a CNS-permeable Reactivator for Nerve Agent Exposure: An Iterative, Multi-Disciplinary Approach

Brian J. Bennion[a], Michael A. Malfatti[a], Nicholas A. Be[a], Heather A. Enright[a], Saphon Hok[b,c], C. Linn Cadieux[d], Timothy S. Carpenter[a], Victoria Lao[a], Edward A. Kuhn[a], M. Windy. McNerney[a], Felice C. Lightstone[a], Tuan H. Nguyen[e], Carlos A. Valdez[b,c,*]

Table of Contents

Content	Page
Table of contents	S1
^{1}H NMR spectrum for LLNL-02	S11
^{13}C NMR spectrum for LLNL-02	S12
^{1}H NMR spectrum for compound **7**	S13
^{13}C NMR spectrum for compound **7**	S14
^{1}H NMR spectrum for compound **8**	S15
^{13}C NMR spectrum for compound **8**	S16
^{1}H NMR spectrum for compound **9**	S17
^{13}C NMR spectrum for compound **9**	S18
^{1}H NMR spectrum for compound **10**	S19
^{13}C NMR spectrum for compound **10**	S20
^{1}H NMR spectrum for compound **11**	S21
^{13}C NMR spectrum for compound **11**	S22
^{1}H NMR spectrum for compound **12**	S23
^{13}C NMR spectrum for compound **12**	S24
^{1}H NMR spectrum for compound **13**	S25
^{13}C NMR spectrum for compound **13**	S26
^{1}H NMR spectrum for compound **14**	S27
^{13}C NMR spectrum for compound **14**	S28
^{1}H NMR spectrum for compound **15**	S29
^{13}C NMR spectrum for compound **15**	S30
^{1}H NMR spectrum for compound **16**	S31
^{13}C NMR spectrum for compound **16**	S32
^{1}H NMR spectrum for compound **17**	S33
^{13}C NMR spectrum for compound **17**	S34
^{1}H NMR spectrum for compound **18**	S35
^{13}C NMR spectrum for compound **18**	S36
^{1}H NMR spectrum for compound **19**	S37
^{13}C NMR spectrum for compound **19**	S38
^{1}H NMR spectrum for compound **20**	S39
^{13}C NMR spectrum for compound **20**	S40
^{1}H NMR spectrum for compound **21**	S41
^{13}C NMR spectrum for compound **21**	S42

^{1}H NMR spectrum for compound **22**	S43
^{13}C NMR spectrum for compound **22**	S44
^{1}H NMR spectrum for compound **23**	S45
^{13}C NMR spectrum for compound **23**	S46
^{1}H NMR spectrum for compound **24**	S47
^{13}C NMR spectrum for compound **24**	S48
^{1}H NMR spectrum for compound **25**	S49
^{13}C NMR spectrum for compound **25**	S50
^{1}H NMR spectrum for compound **26**	S51
^{13}C NMR spectrum for compound **26**	S52
^{1}H NMR spectrum for compound **27**	S53
^{13}C NMR spectrum for compound **27**	S54
^{1}H NMR spectrum for compound **28**	S55
^{13}C NMR spectrum for compound **28**	S56
^{1}H NMR spectrum for compound **29**	S57
^{13}C NMR spectrum for compound **29**	S58
^{1}H NMR spectrum for compound **30**	S59
^{13}C NMR spectrum for compound **30**	S60
^{1}H NMR spectrum for compound **31**	S61
^{13}C NMR spectrum for compound **31**	S62
^{1}H NMR spectrum for compound **35**	S63
^{13}C NMR spectrum for compound **35**	S64

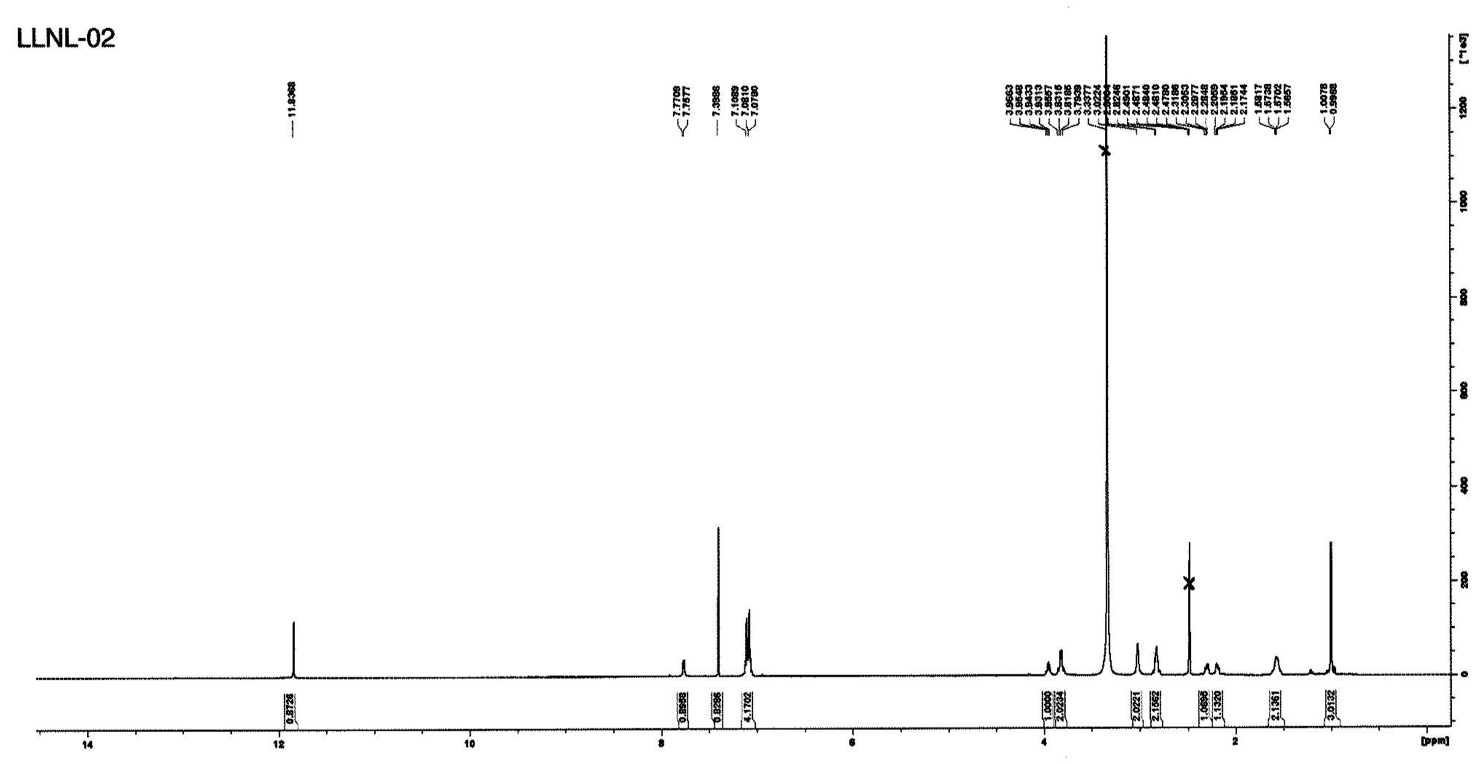

Figure S1. ^{1}H NMR for LLNL-02.

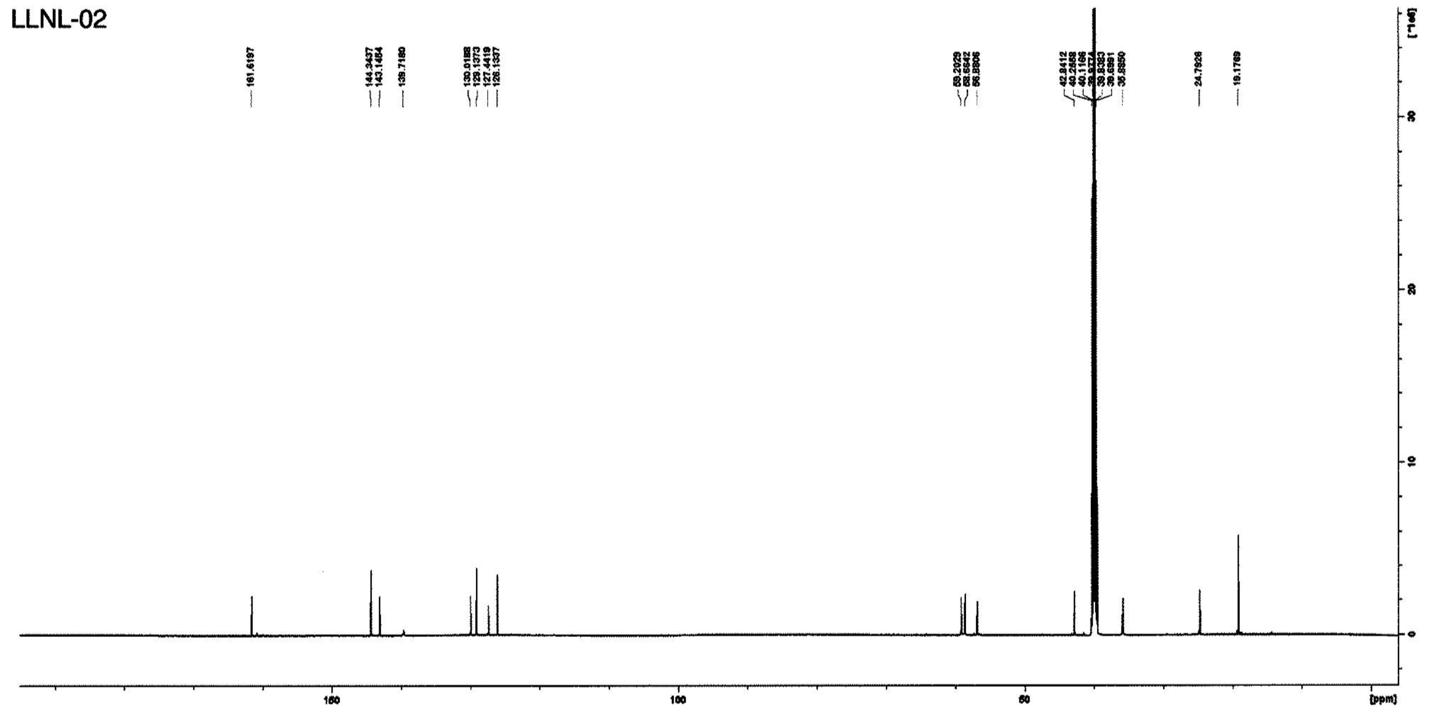

Figure S2. ^{13}C NMR for LLNL-02.

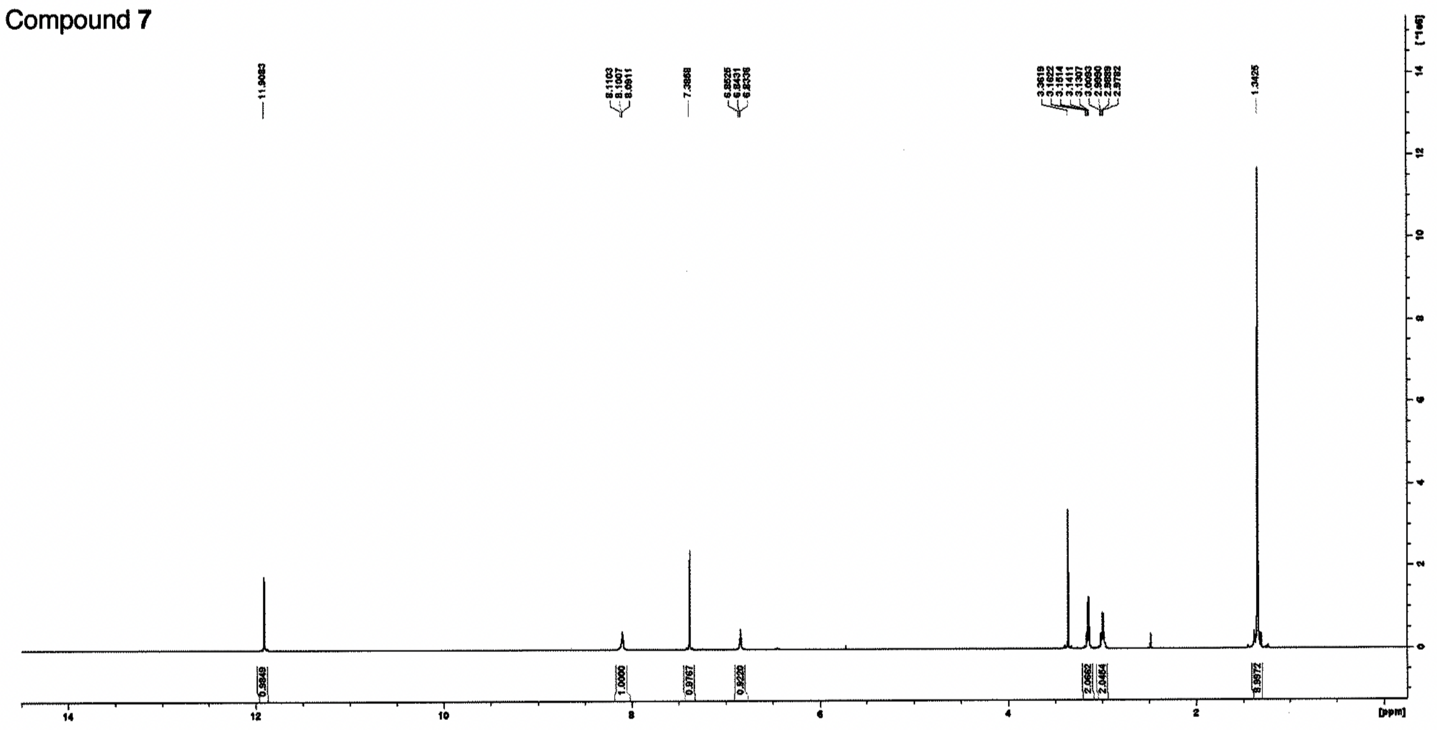

Figure S3. ^{1}H NMR for *tert*-butyl (*E*)-(2-(2-(hydroxyimino)acetamido)ethyl)carbamate.

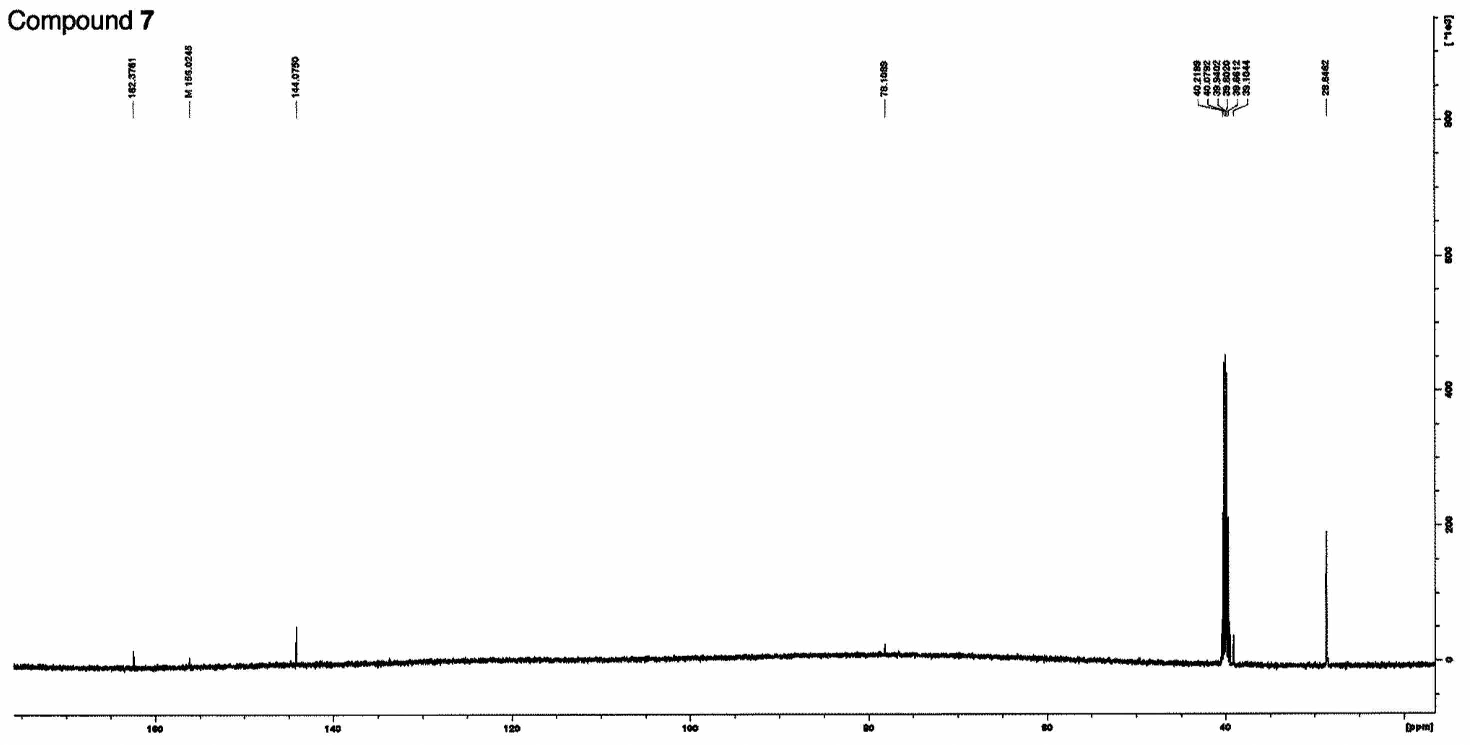

Figure S4. ^{13}C NMR for *tert*-butyl (*E*)-(2-(2-(hydroxyimino)acetamido)ethyl)carbamate.

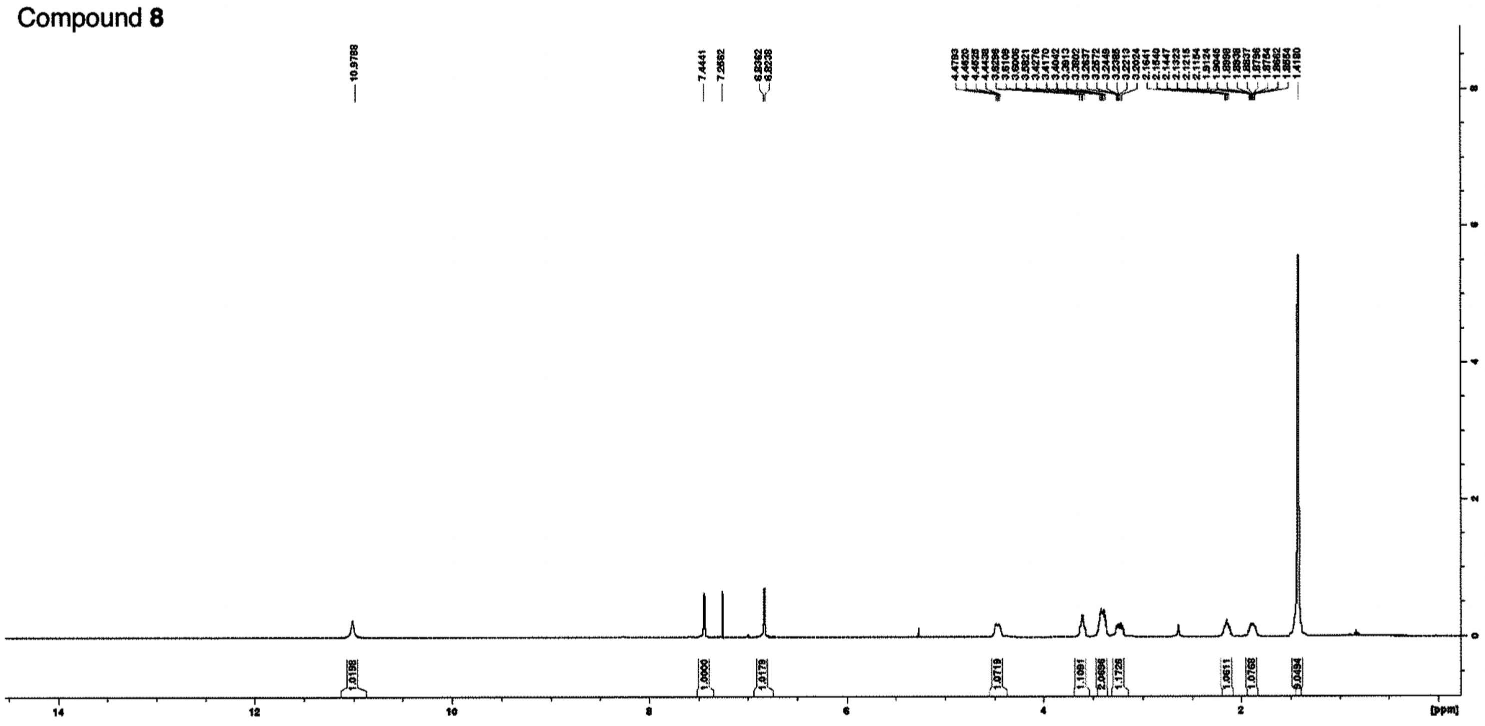

Figure S5. ^{1}H NMR for *tert*-butyl (*R*,*E*)-3-(2-(hydroxyimino)acetamido)pyrrolidine-1-carboxylate.

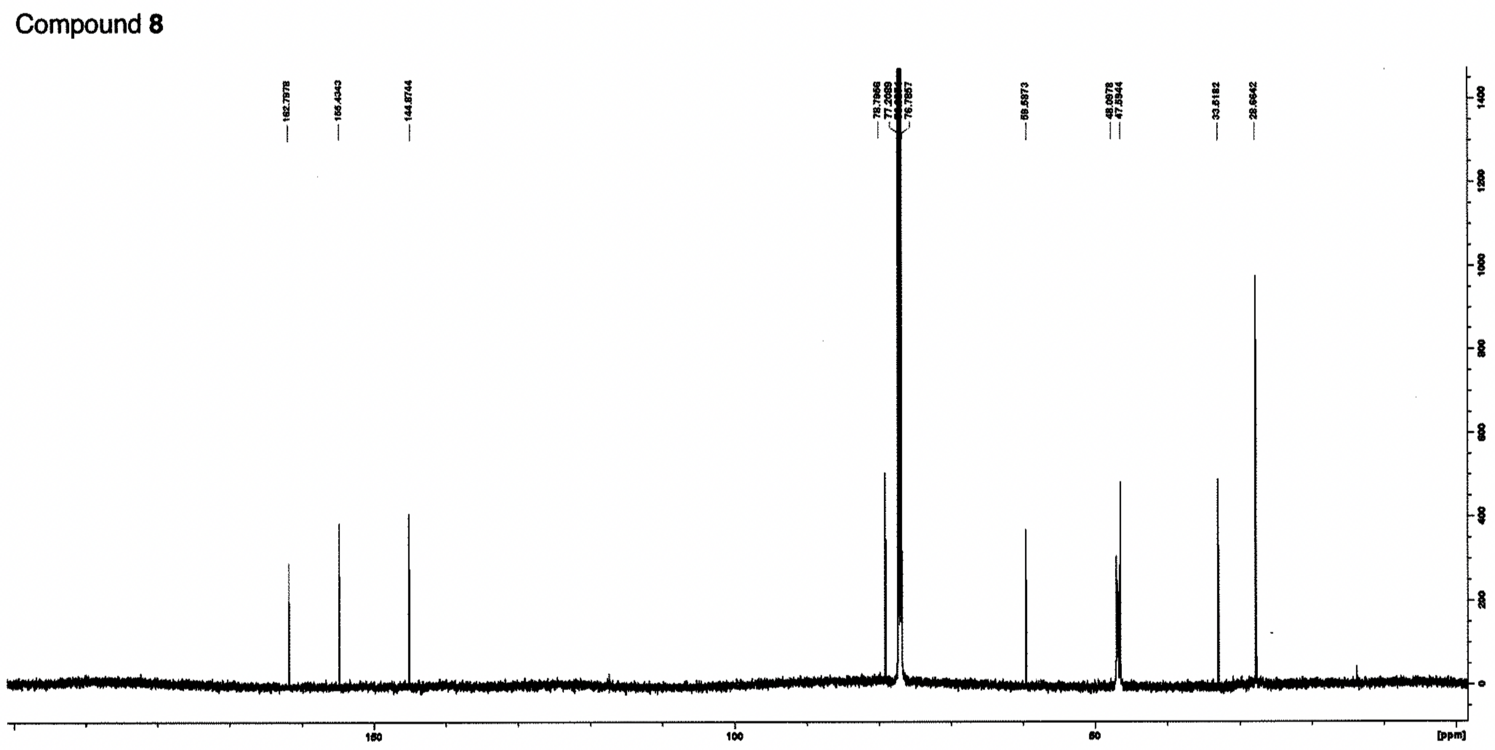

Figure S6. ^{13}C NMR for *tert*-butyl (*R*,*E*)-3-(2-(hydroxyimino)acetamido)pyrrolidine-1-carboxylate.

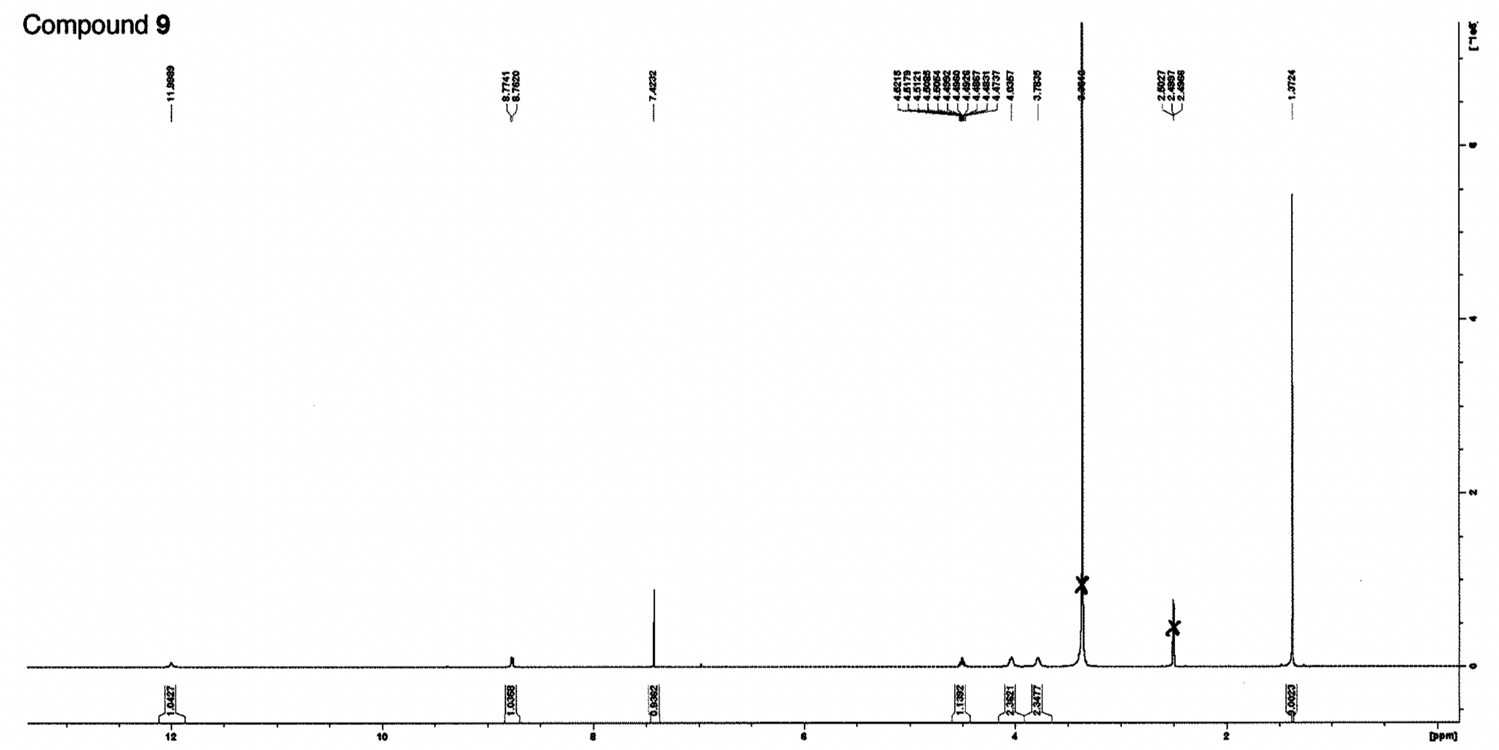

Figure S7. ^{1}H NMR for *tert*-butyl (*E*)-3-(2-(hydroxyimino)acetamido)azetidine-1-carboxylate.

Compound 9

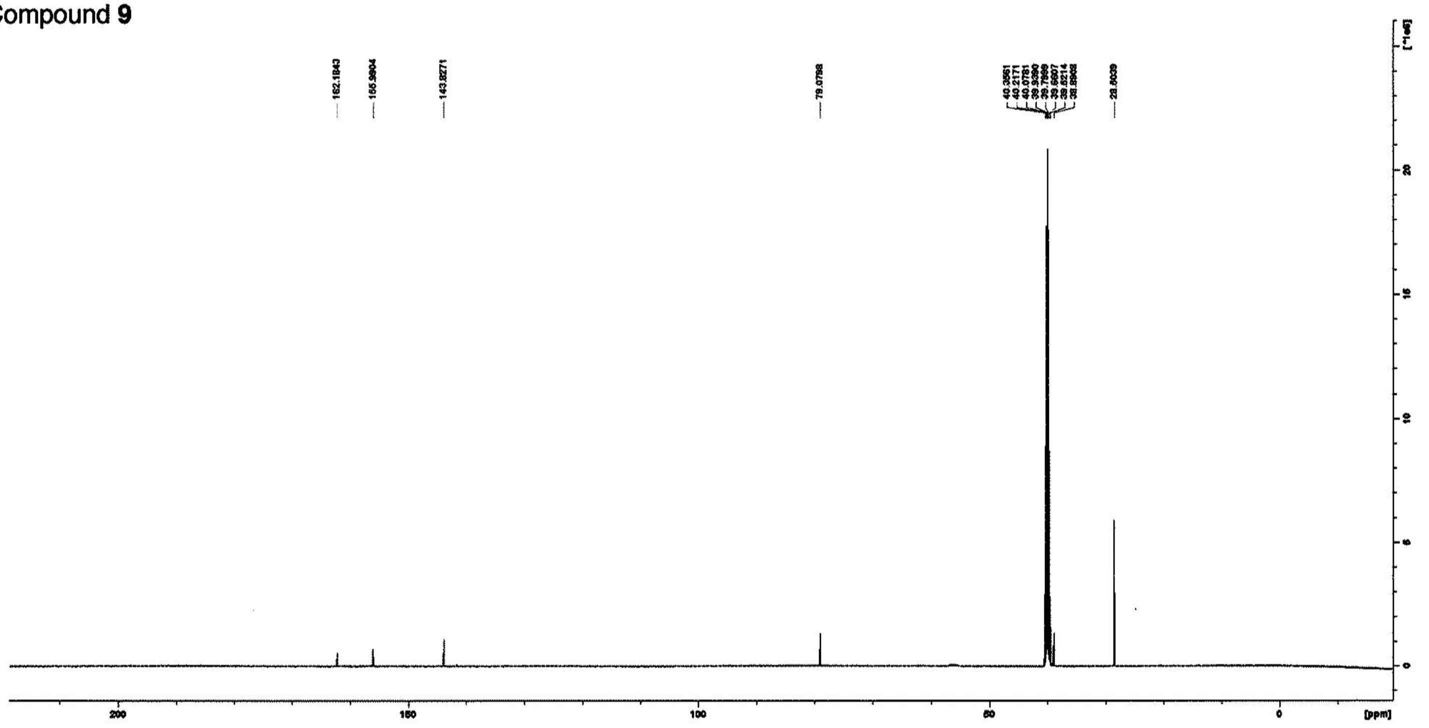

Figure S8. ^{13}C NMR for *tert*-butyl (*E*)-3-(2-(hydroxyimino)acetamido)azetidine-1-carboxylate.

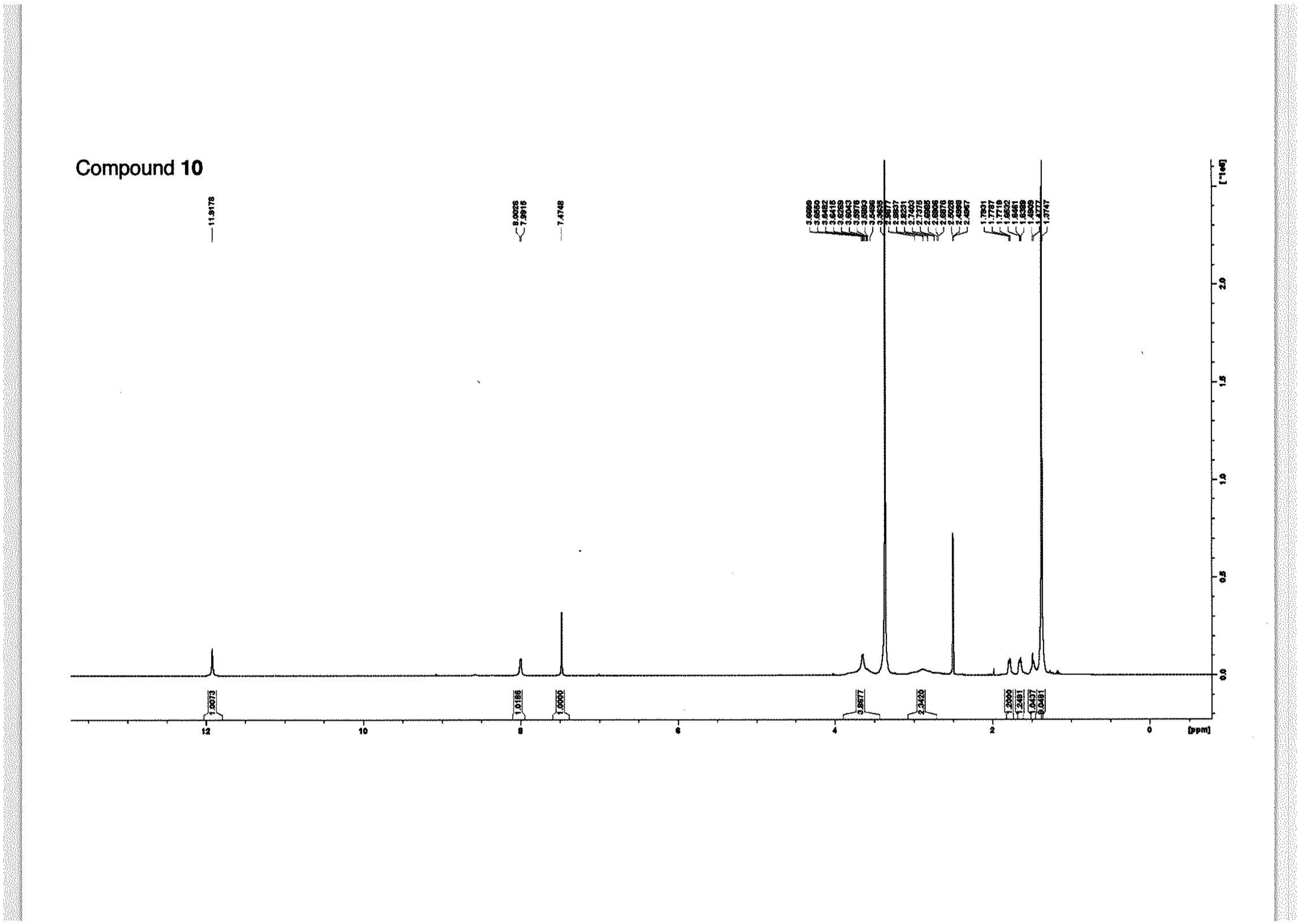

Figure S9. ^{1}H NMR for *tert*-butyl (*E*)-3-(2-(hydroxyimino)acetamido)piperidine-1-carboxylate.

Compound **10**

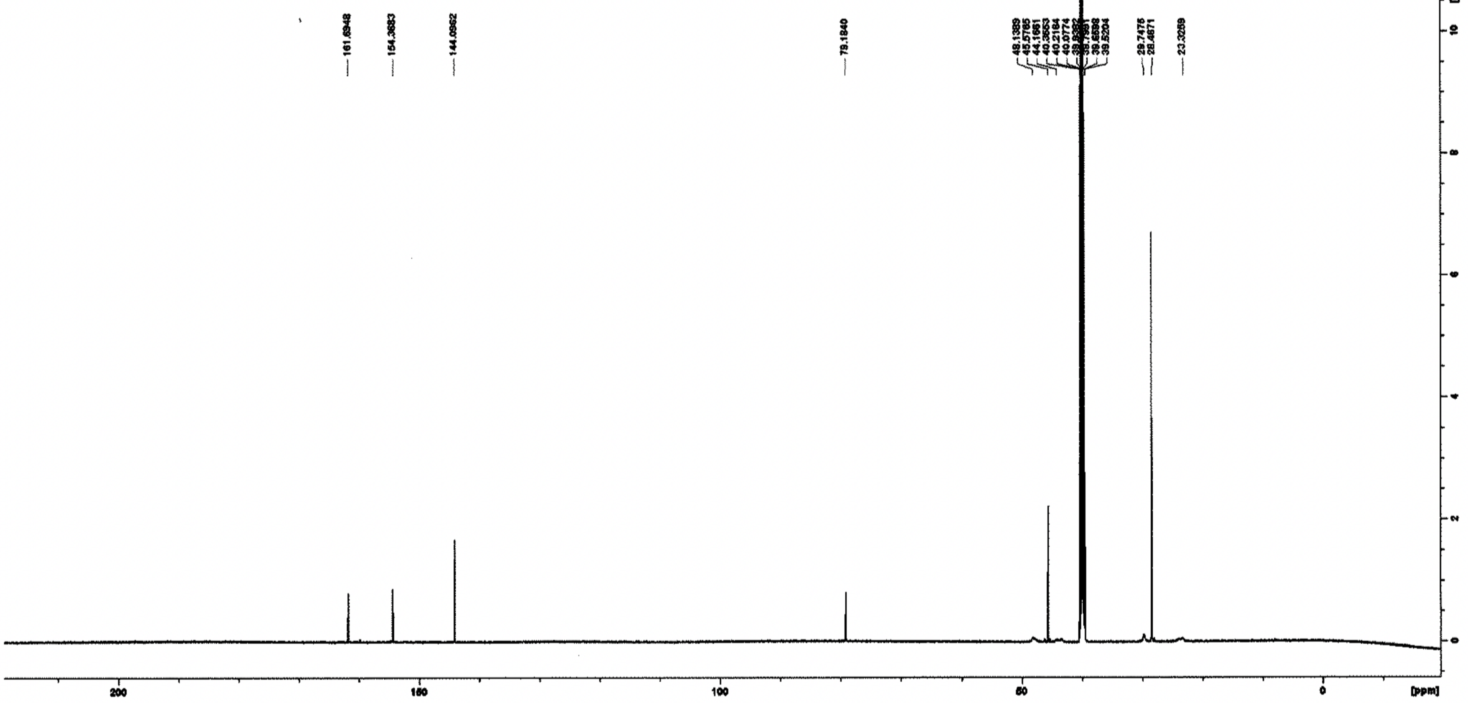

Figure S10. ^{13}C NMR for *tert*-butyl (*E*)-3-(2-(hydroxyimino)acetamido)piperidine-1-carboxylate.

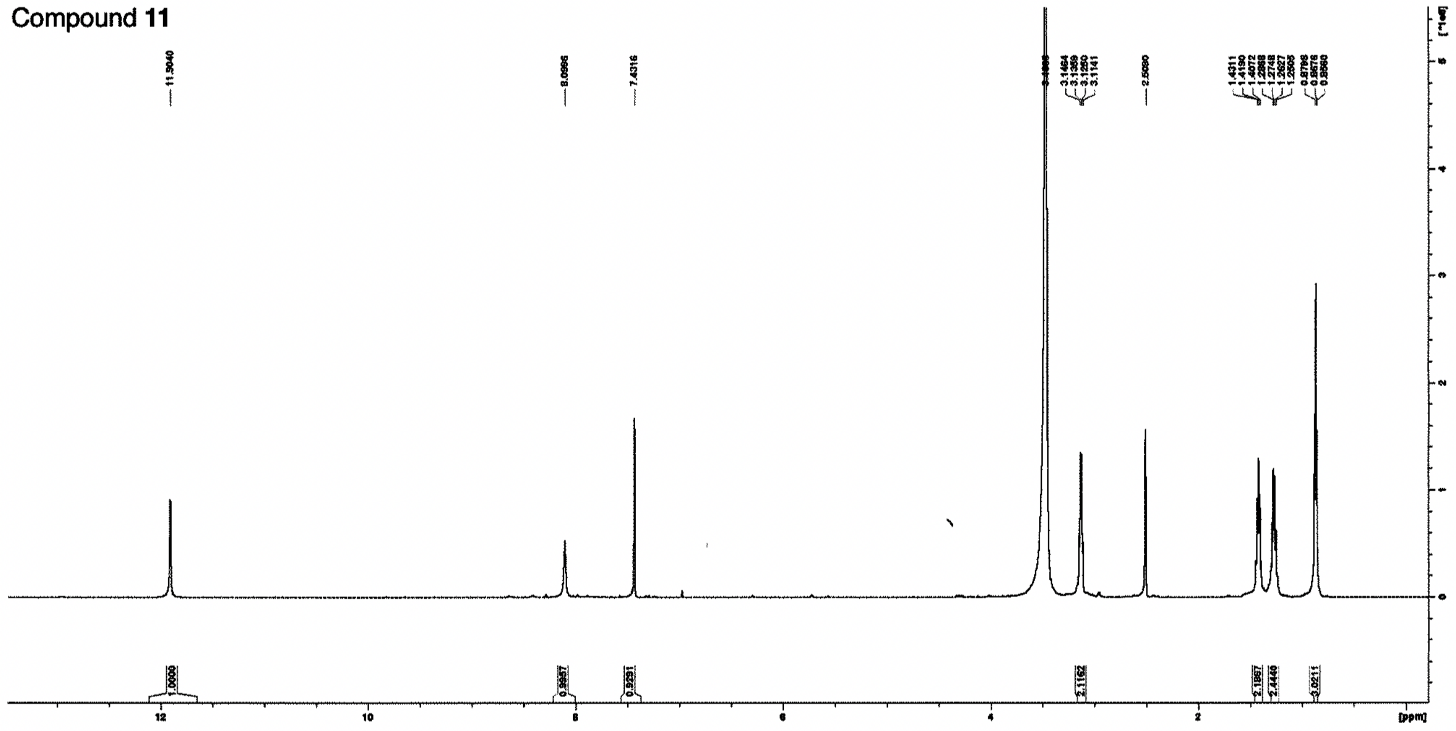

Figure S11. ^{1}H NMR for (*E*)-*N*-butyl-2-(hydroxyimino)acetamide.

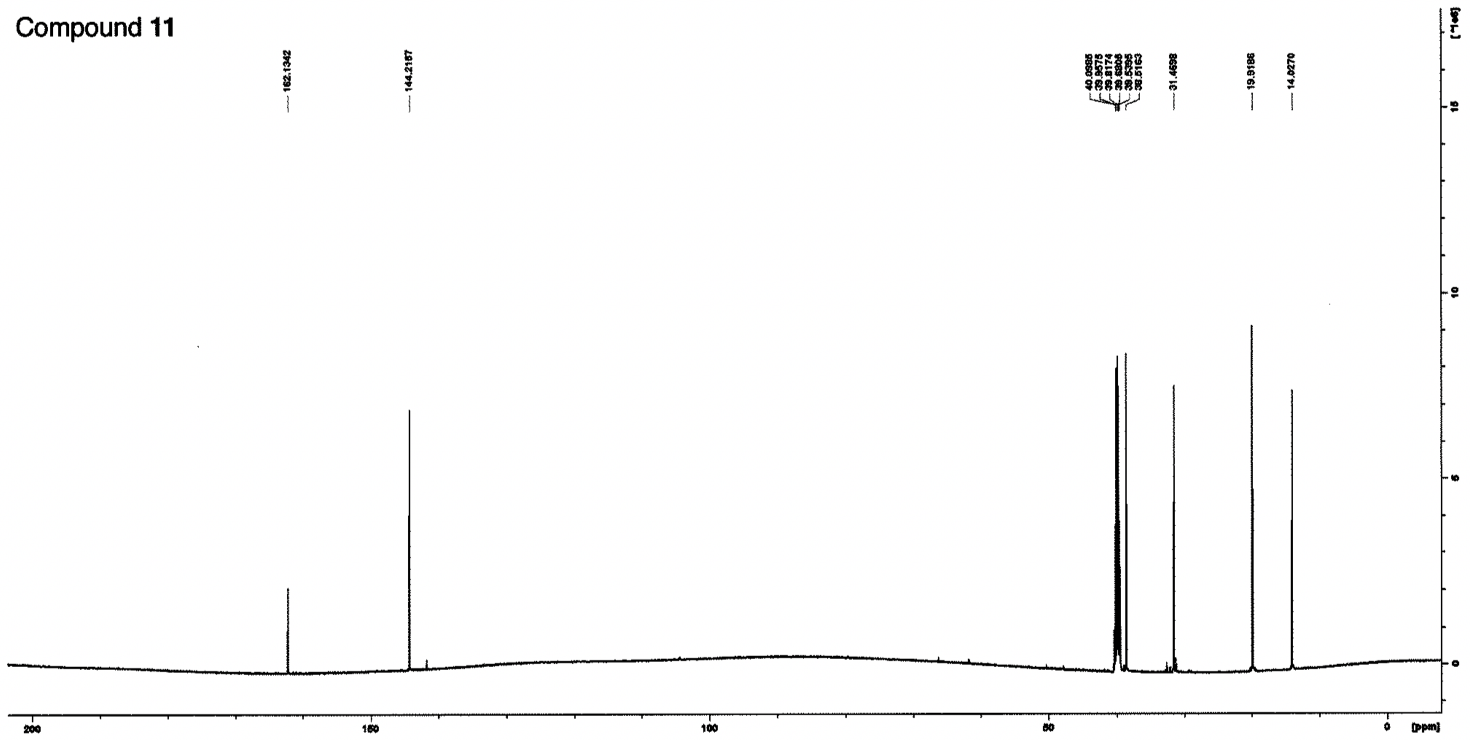

Figure S12. ^{13}C NMR for (*E*)-*N*-butyl-2-(hydroxyimino)acetamide.

Compound **12**

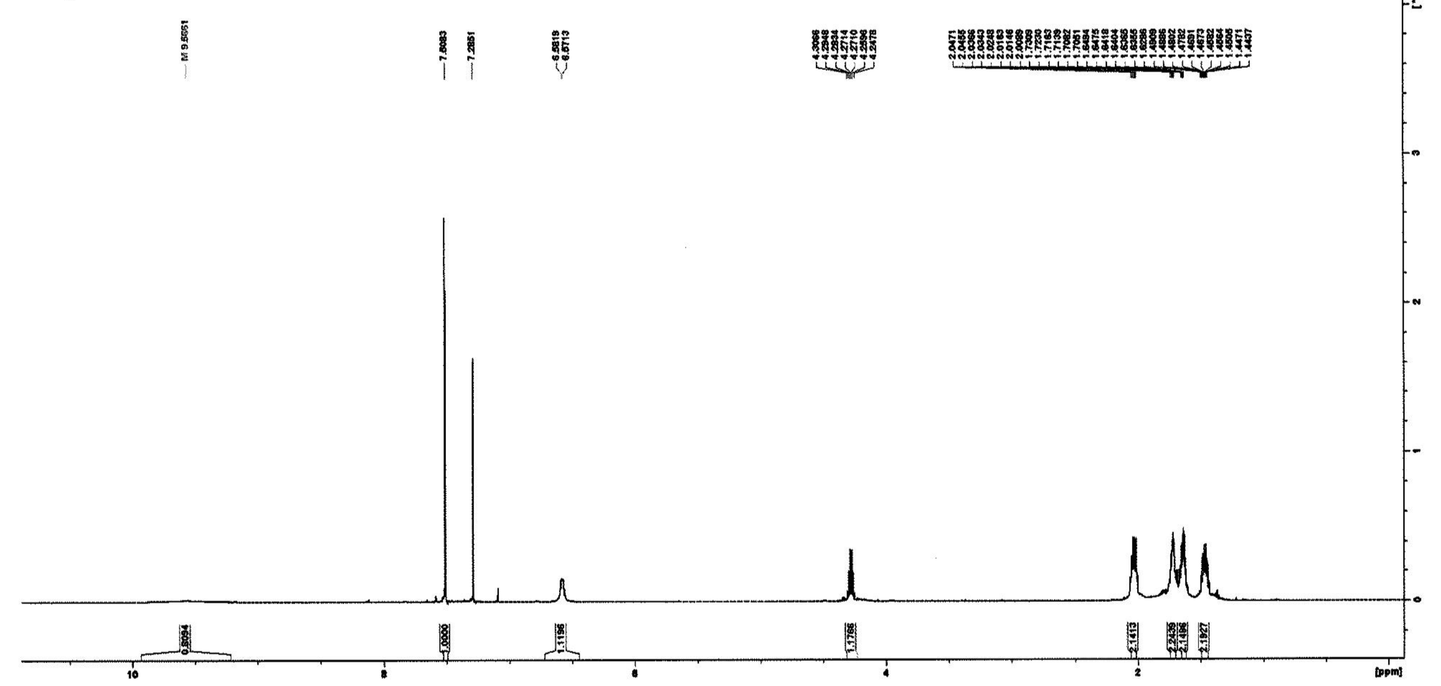

Figure S13. ^{1}H NMR for (*E*)-*N*-cyclopentyl-2-(hydroxyimino)acetamide.

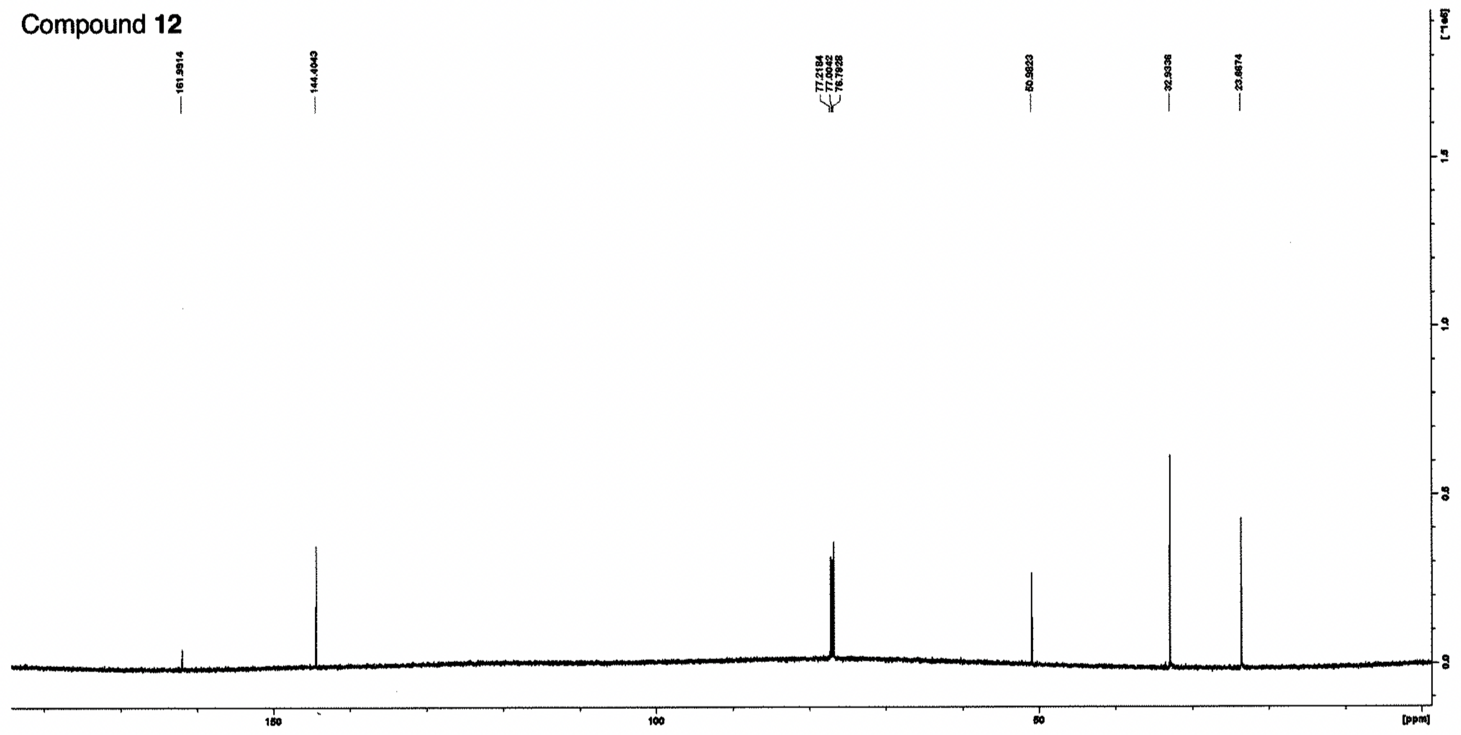

Figure S14. ^{13}C NMR for (*E*)-*N*-cyclopentyl-2-(hydroxyimino)acetamide.

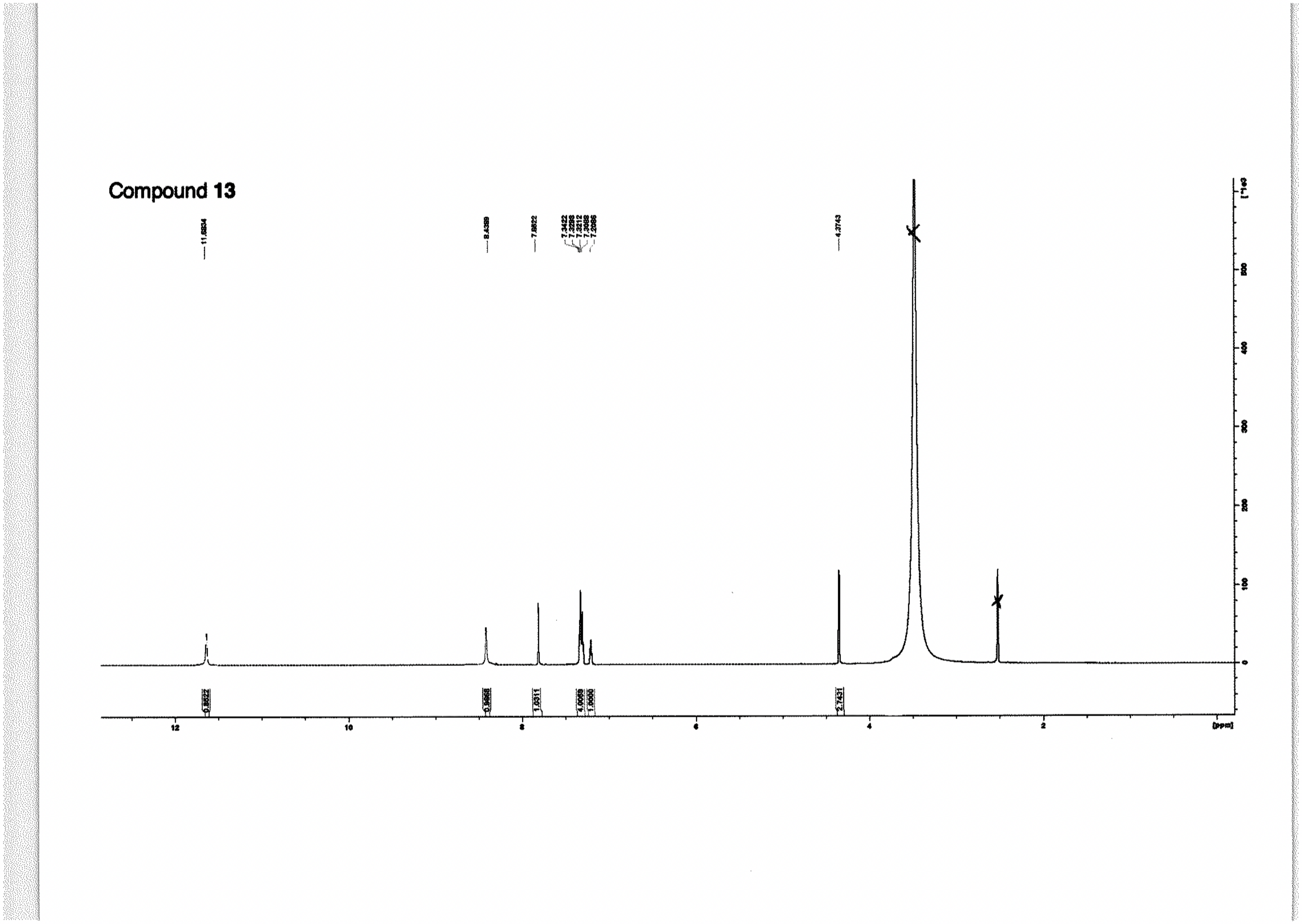

Figure S15. ^{1}H NMR for (*E*)-*N*-benzyl-2-(hydroxyimino)acetamide.

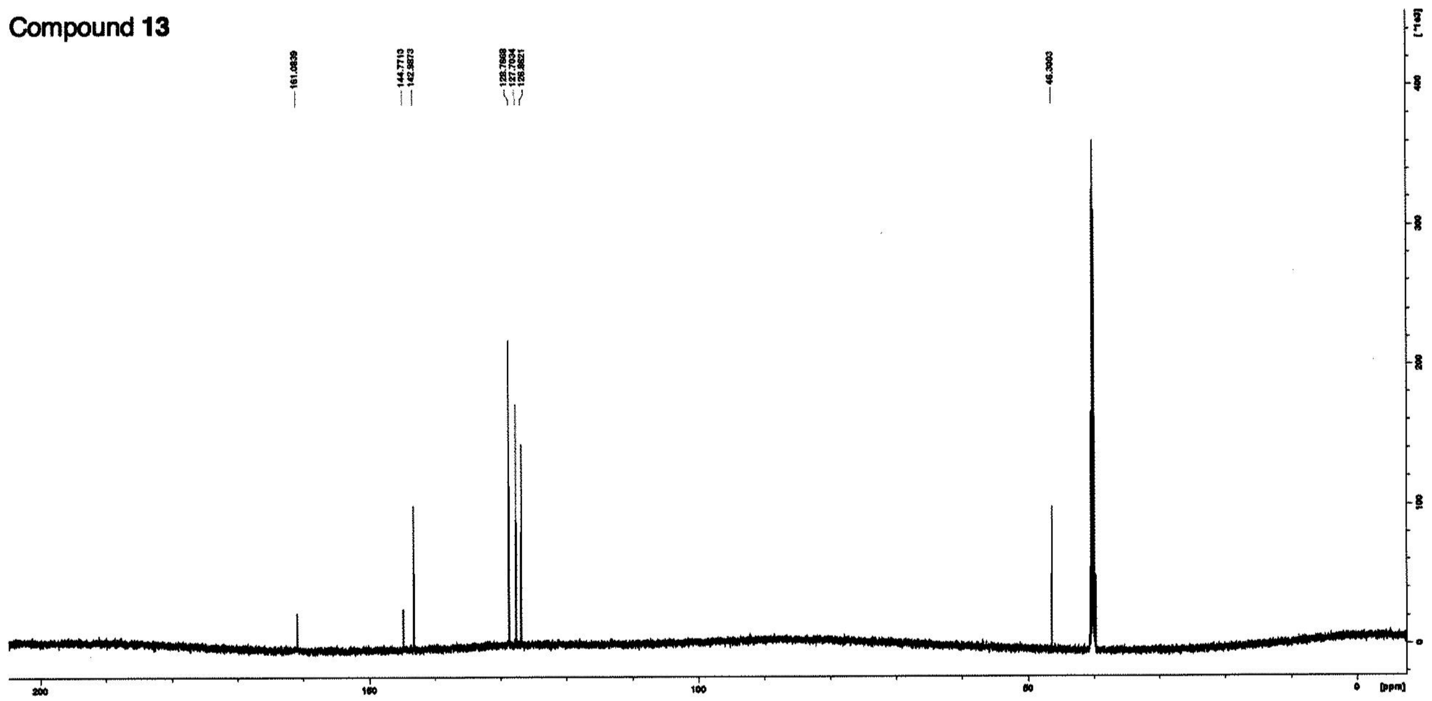

Figure S16. ^{13}C NMR for (*E*)-*N*-benzyl-2-(hydroxyimino)acetamide.

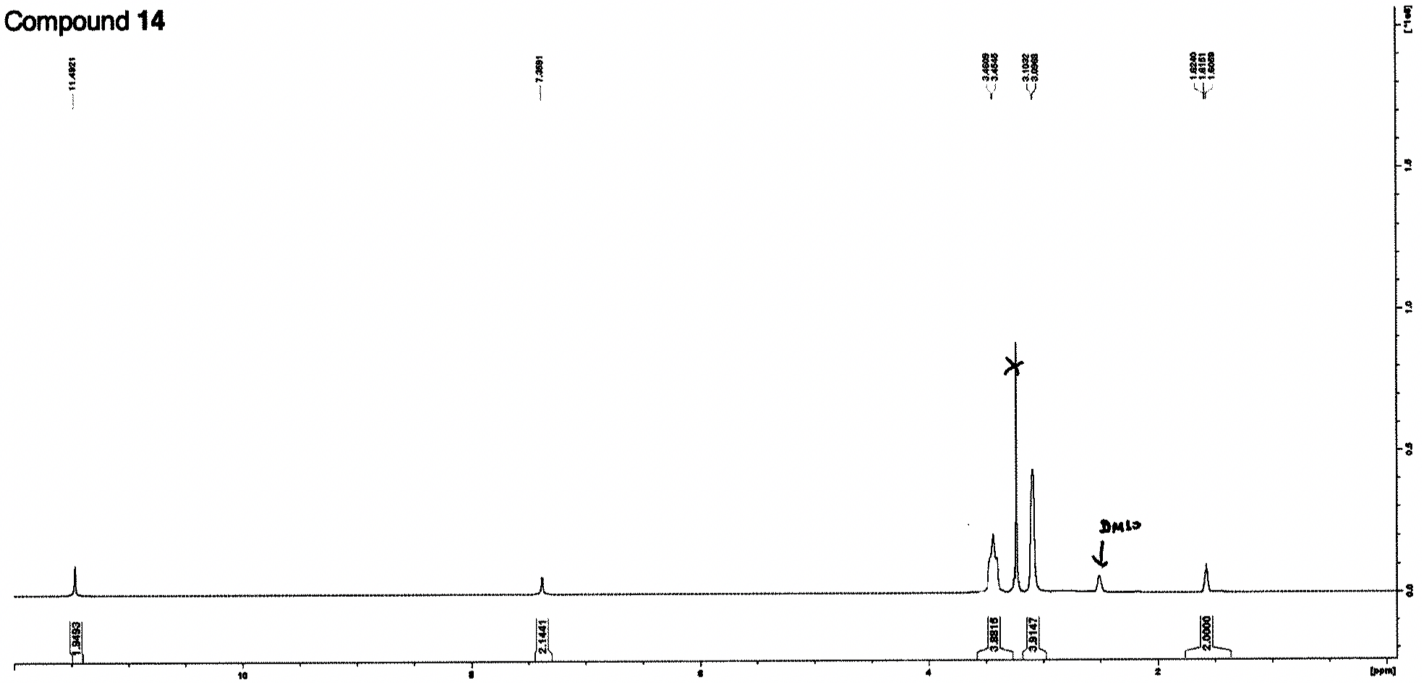

Figure S17. ^{1}H NMR for (1*E*,1'*E*)-2,2'-(1,4-diazepane-1,4-diyl)bis(2-oxoacetaldehyde) dioxime.

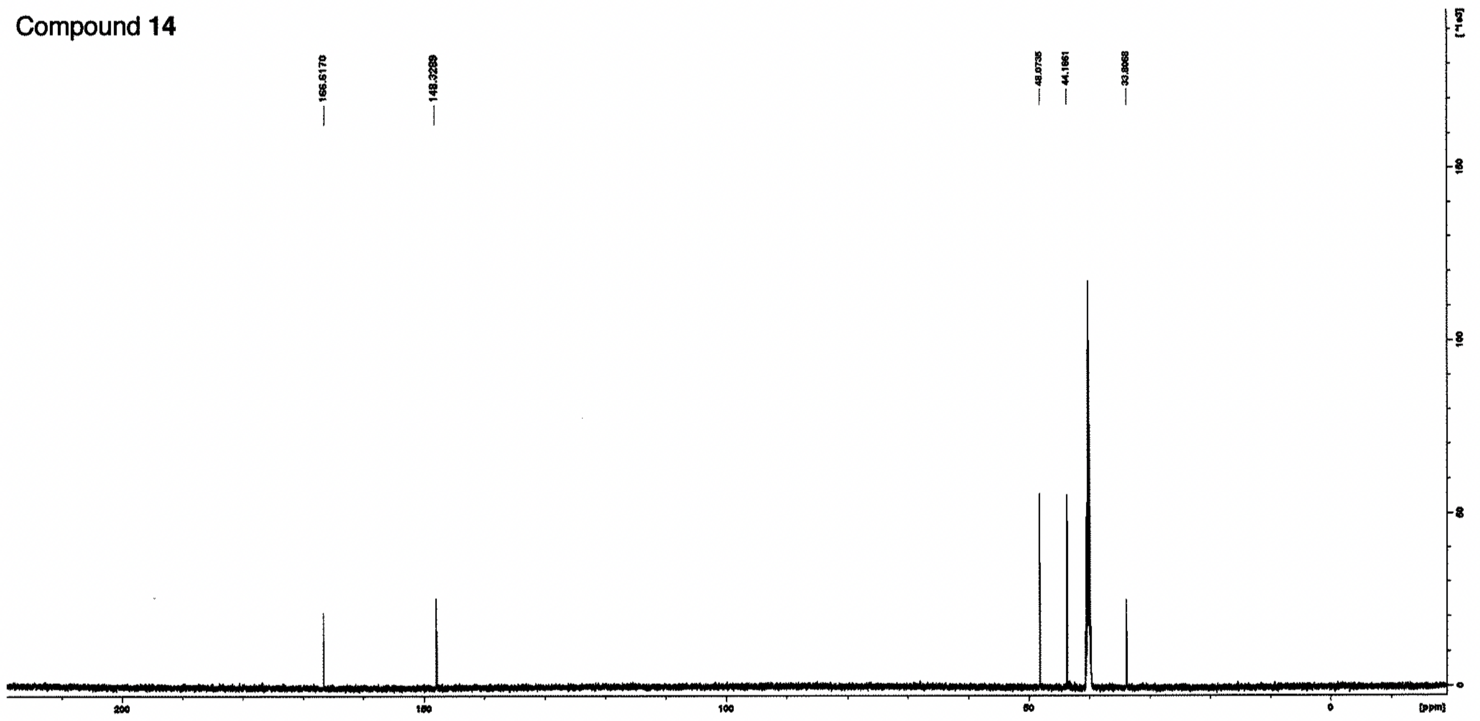

Figure S18. ^{13}C NMR for (1*E*,1'*E*)-2,2'-(1,4-diazepane-1,4-diyl)bis(2-oxoacetaldehyde) dioxime.

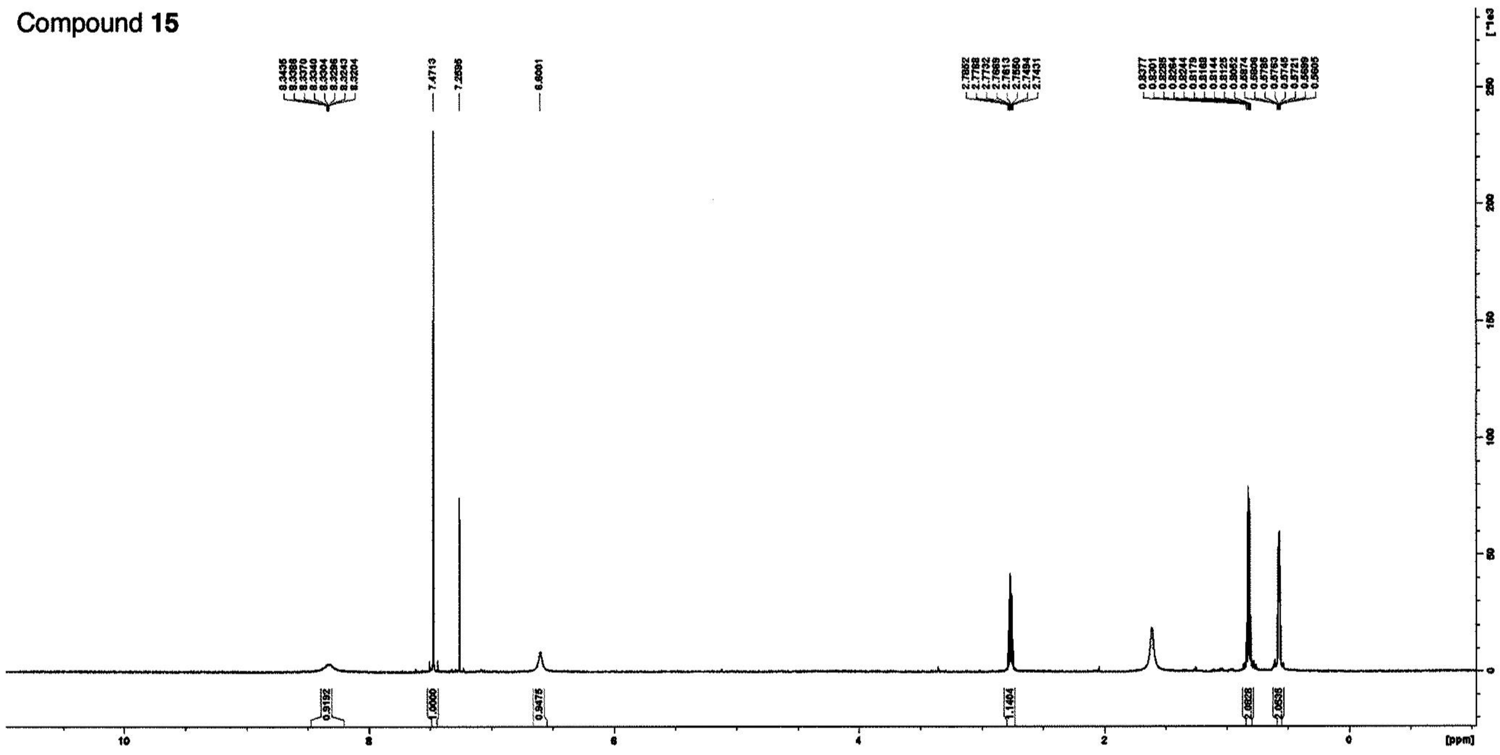

Figure S19. ^{1}H NMR for (*E*)-*N*-cyclopropyl-2-(hydroxyimino)acetamide.

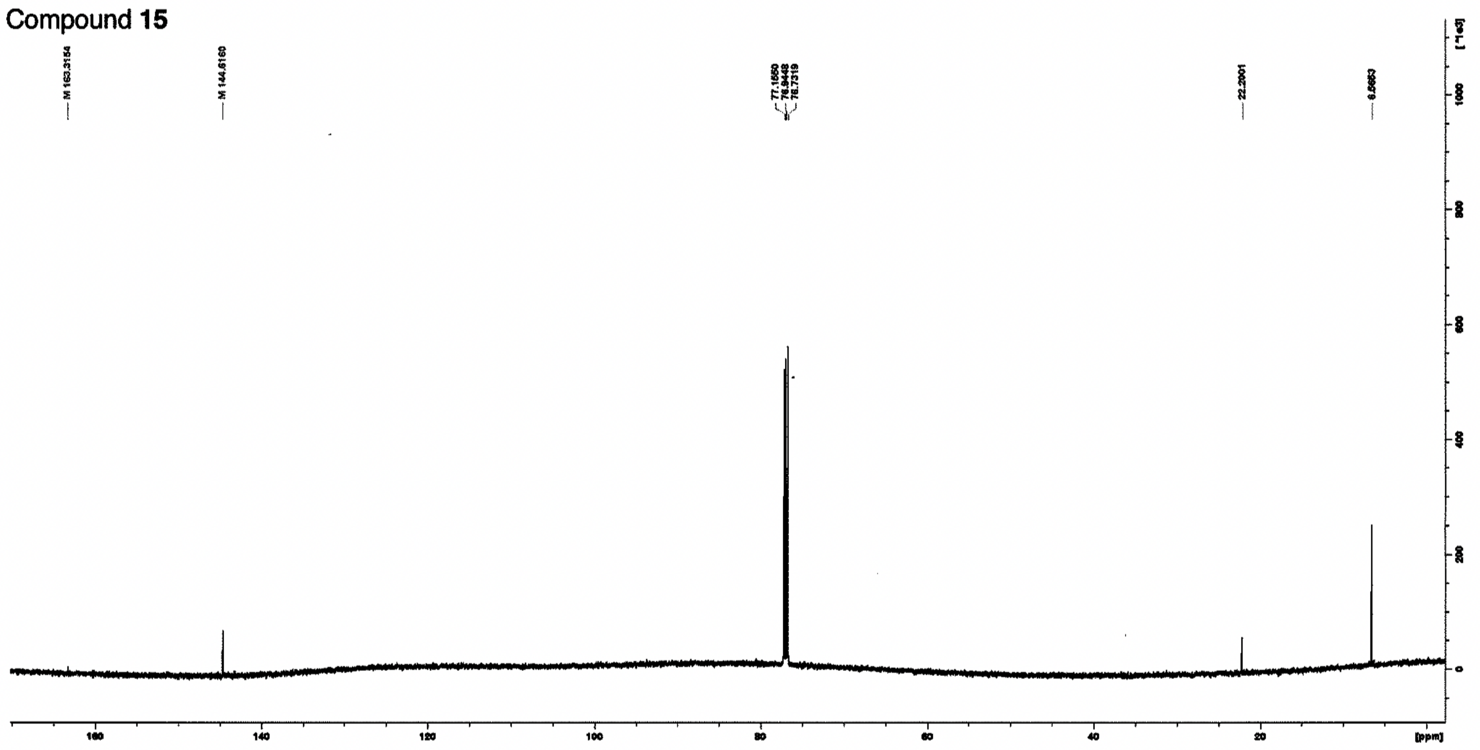

Figure S20. ^{13}C NMR for (*E*)-*N*-cyclopropyl-2-(hydroxyimino)acetamide.

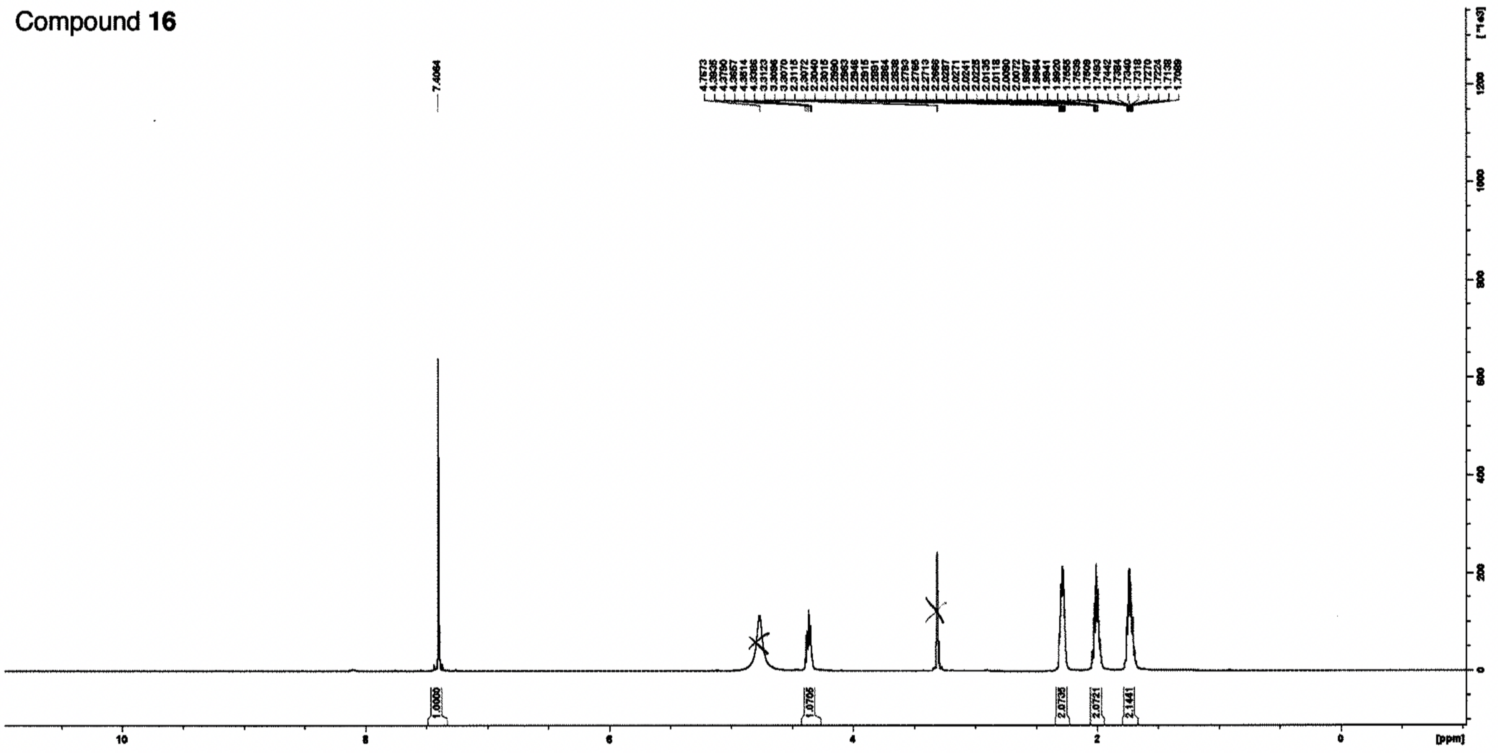

Figure S21. ^{1}H NMR for (*E*)-*N*-cyclobutyl-2-(hydroxyimino)acetamide.

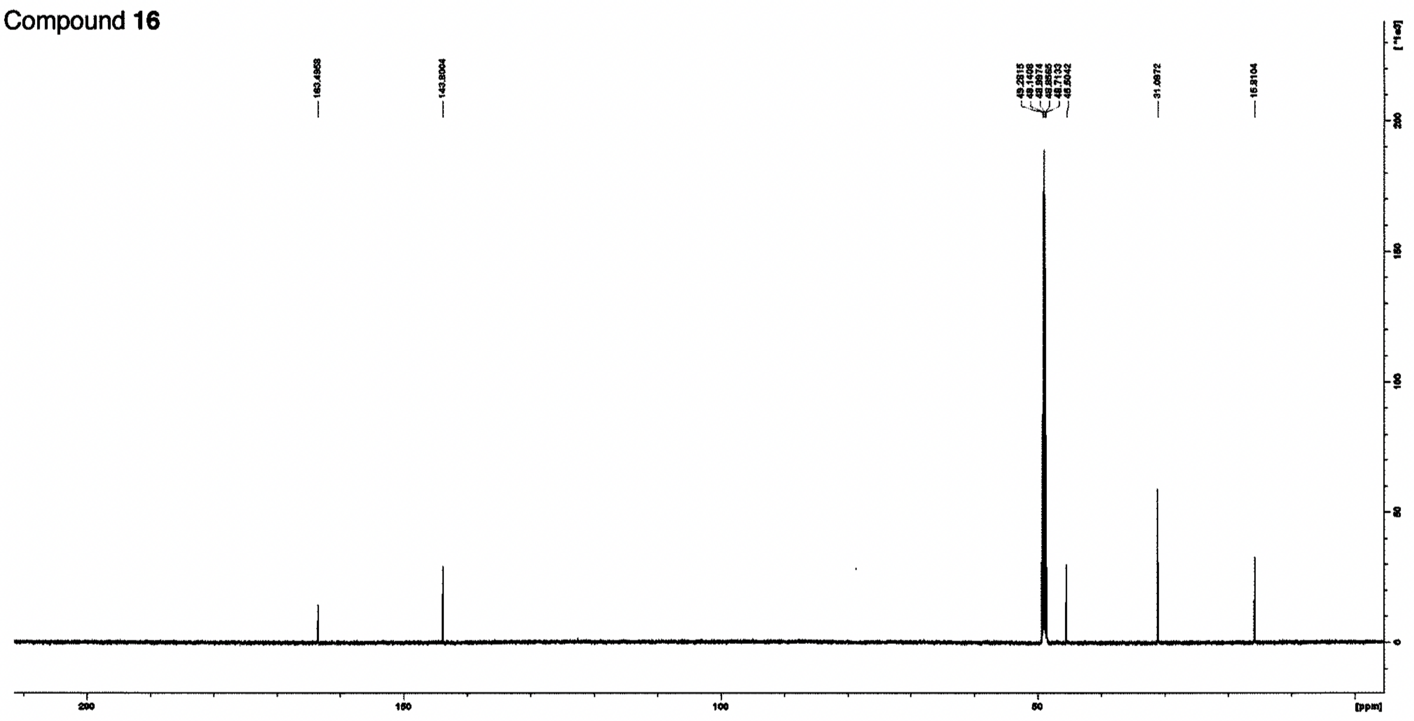

Figure S22. ^{13}C NMR for (*E*)-*N*-cyclobutyl-2-(hydroxyimino)acetamide.

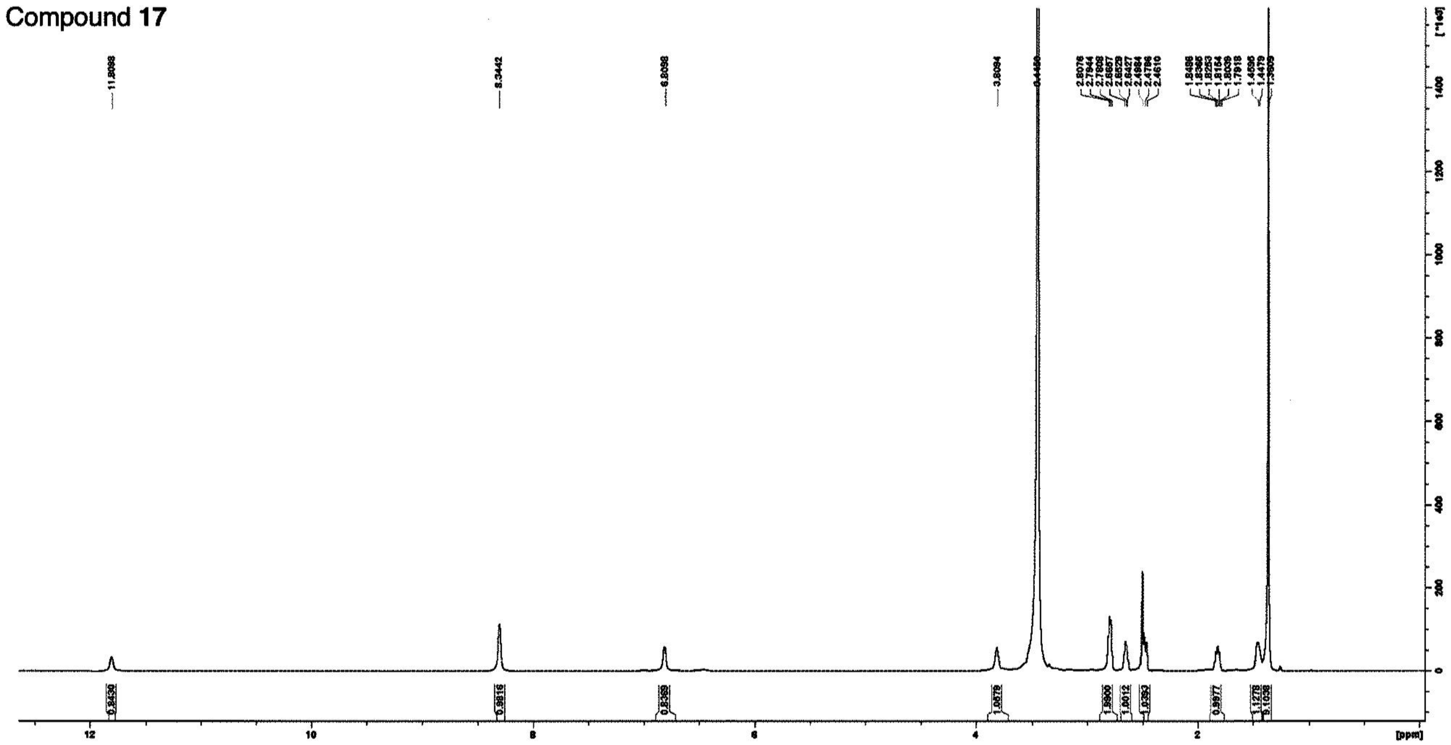

Figure S23. ^{1}H NMR for *tert*-Butyl (E)-(1-(2-(hydroxyimino)acetyl)pyrrolidin-3-yl)carbamate.

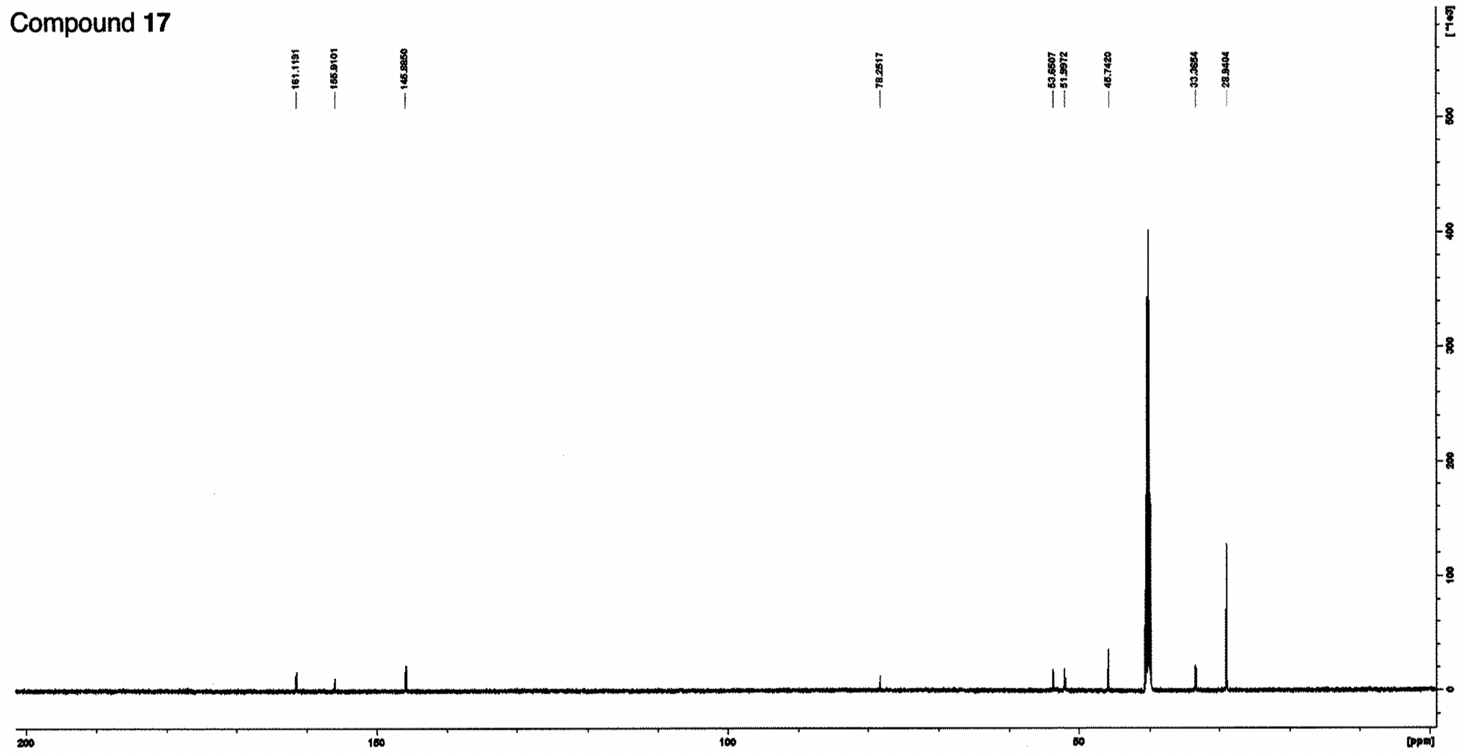

Figure S24. ^{13}C NMR for *tert*-Butyl (E)-(1-(2-(hydroxyimino)acetyl)pyrrolidin-3-yl)carbamate.

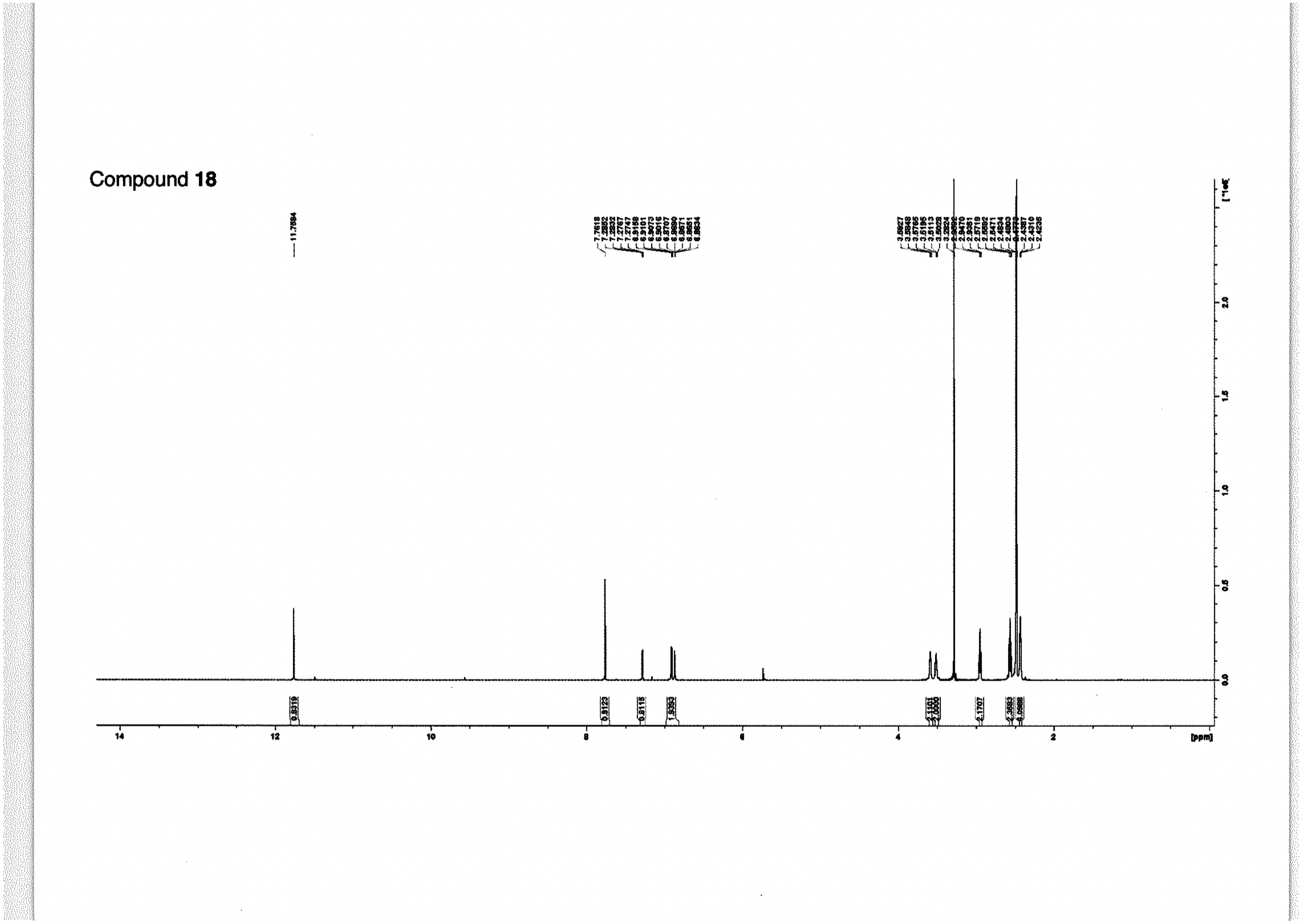

Figure S25. ^{1}H NMR for (*E*)-2-oxo-2-(4-(2-(thiophen-2-yl)ethyl)piperazin-1-yl)acetaldehyde oxime.

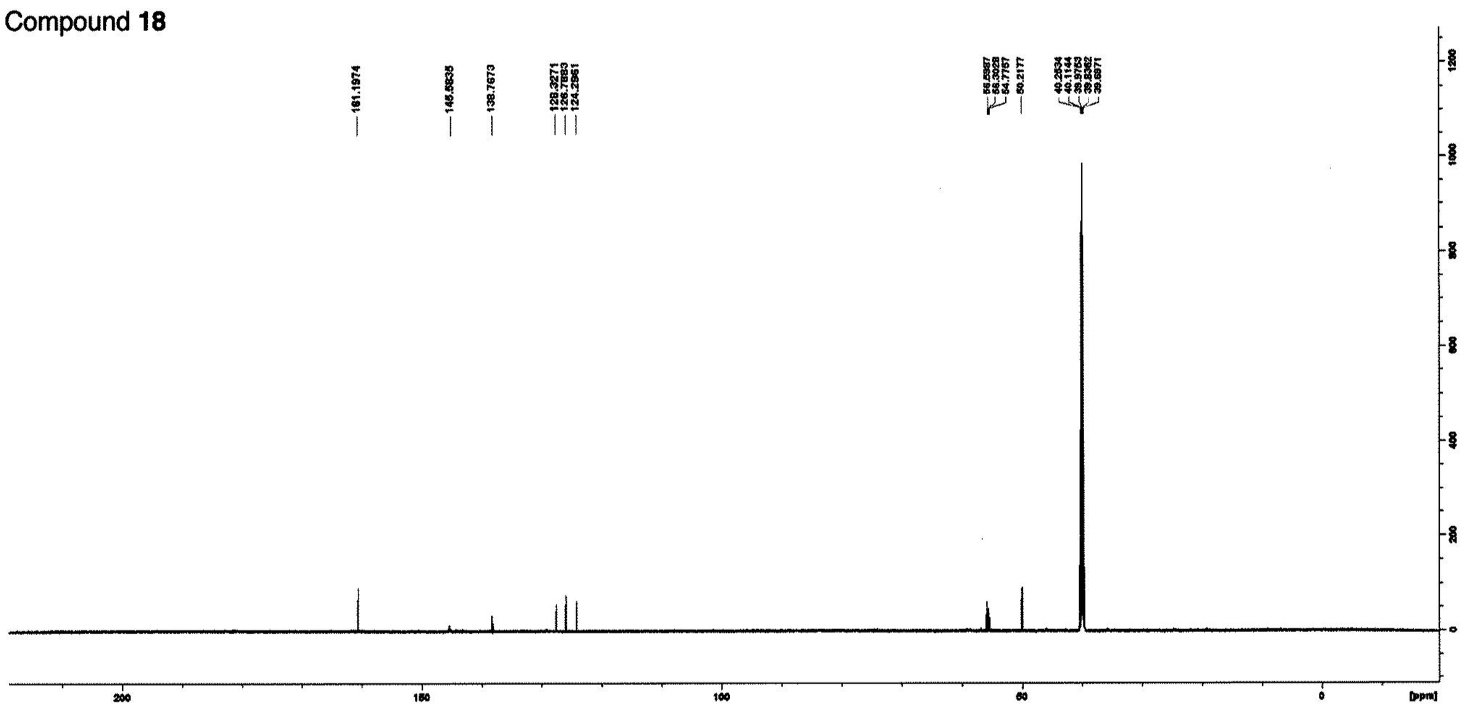

Figure S26. ^{13}C NMR for (*E*)-2-oxo-2-(4-(2-(thiophen-2-yl)ethyl)piperazin-1-yl)acetaldehyde oxime.

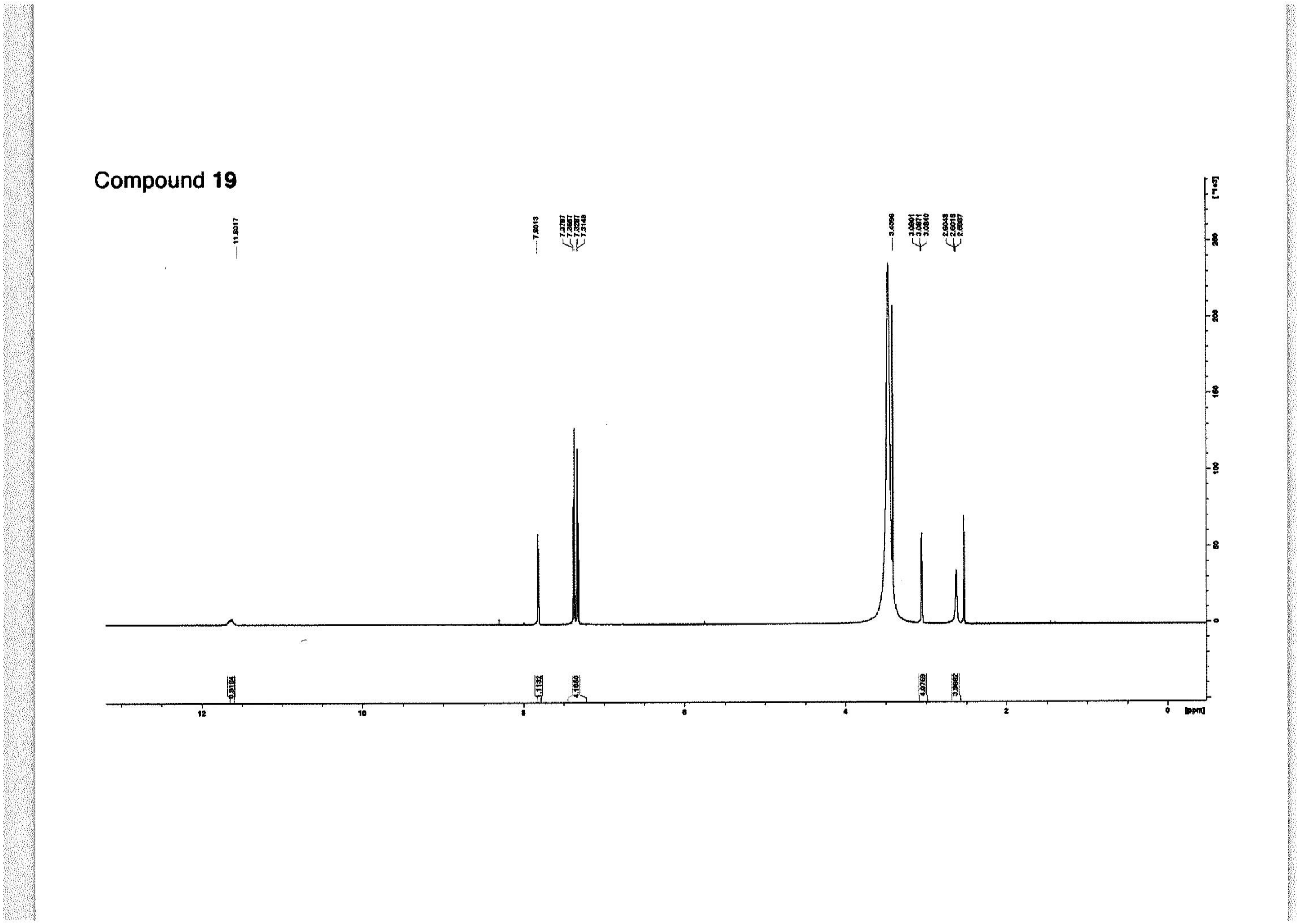

Figure S27. ^{1}H NMR for (*E*)-2-(4-(4-chlorobenzyl)piperazin-1-yl)-2-oxoacetaldehyde oxime.

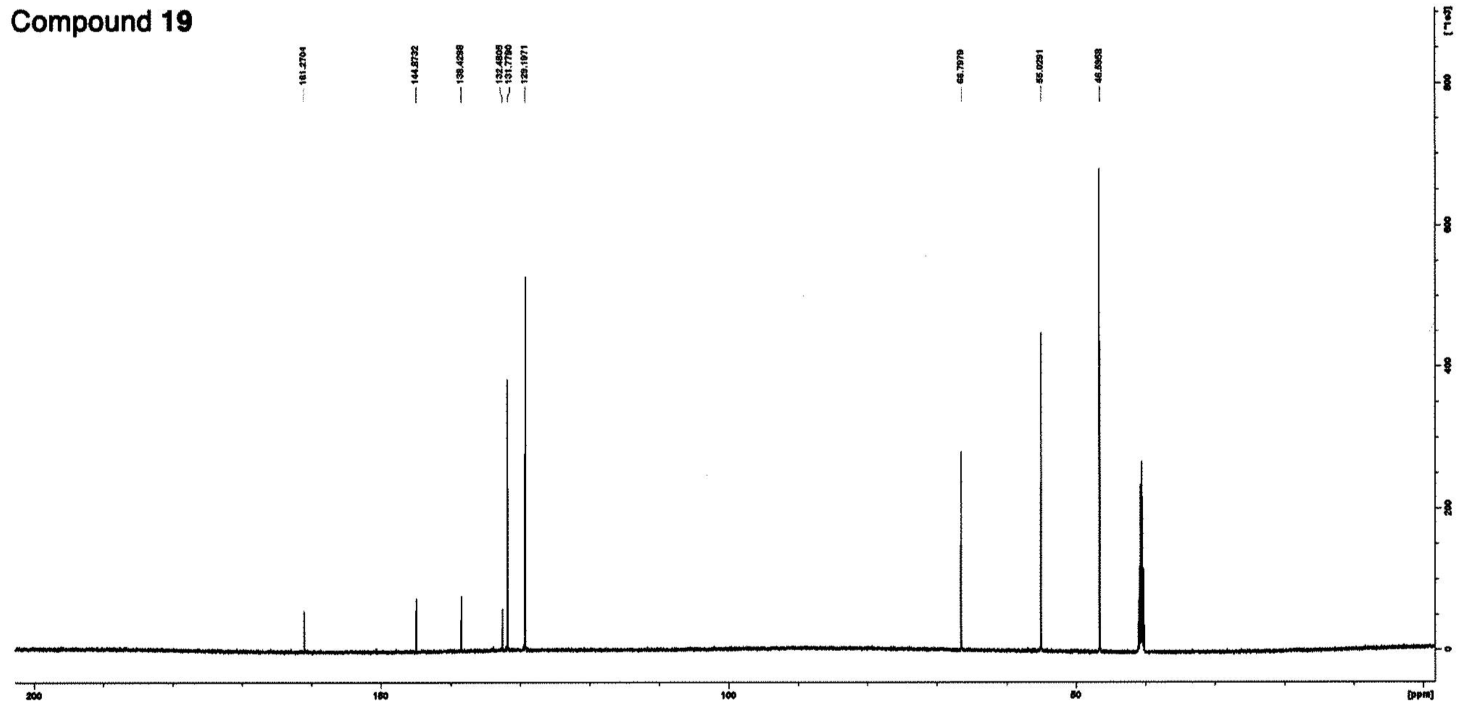

Figure S28. ^{13}C NMR for (*E*)-2-(4-(4-chlorobenzyl)piperazin-1-yl)-2-oxoacetaldehyde oxime.

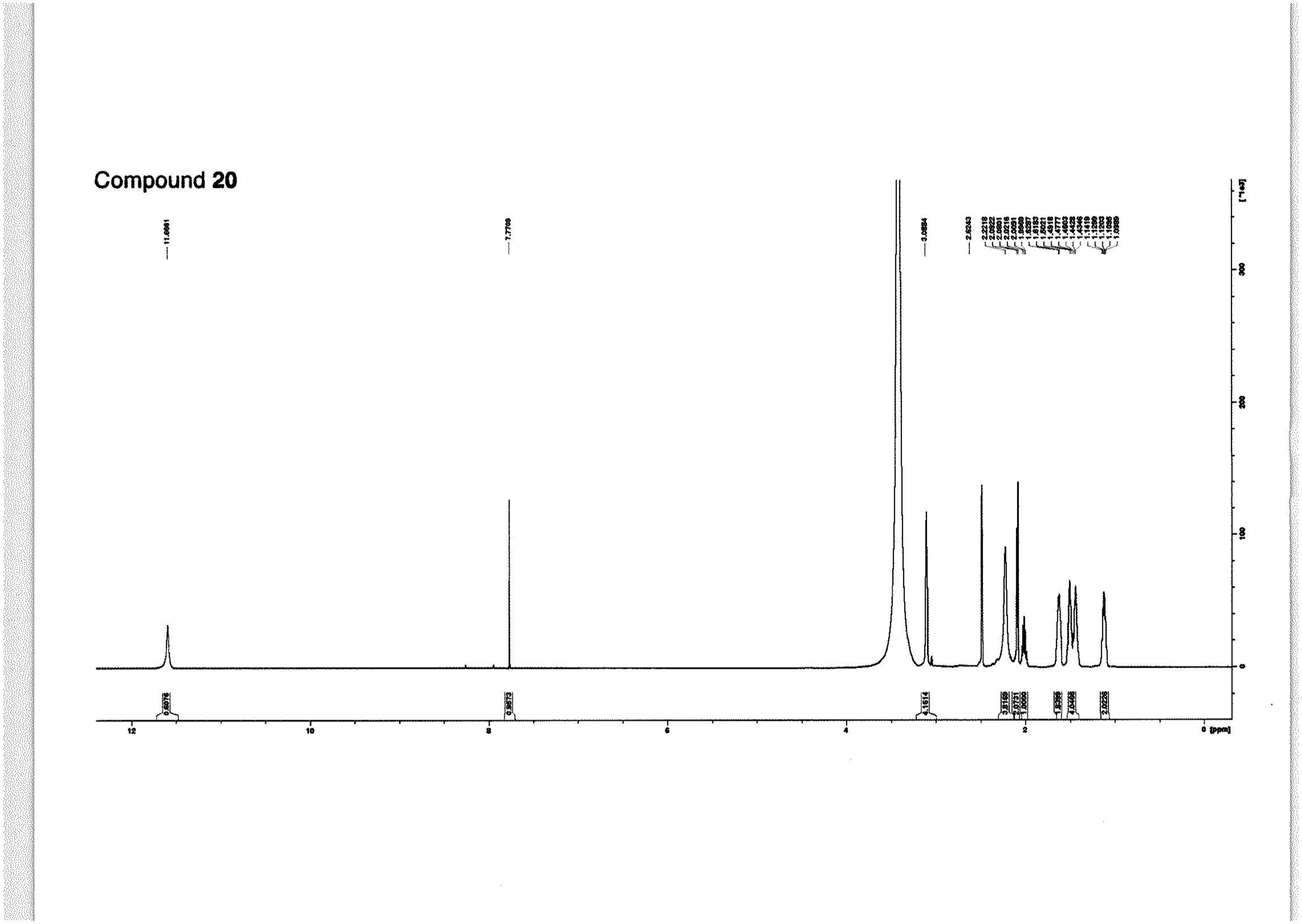

Figure S29. ^{1}H NMR for (*E*)-2-(4-(cyclopentylmethyl)piperazin-1-yl)-2-oxoacetaldehyde oxime.

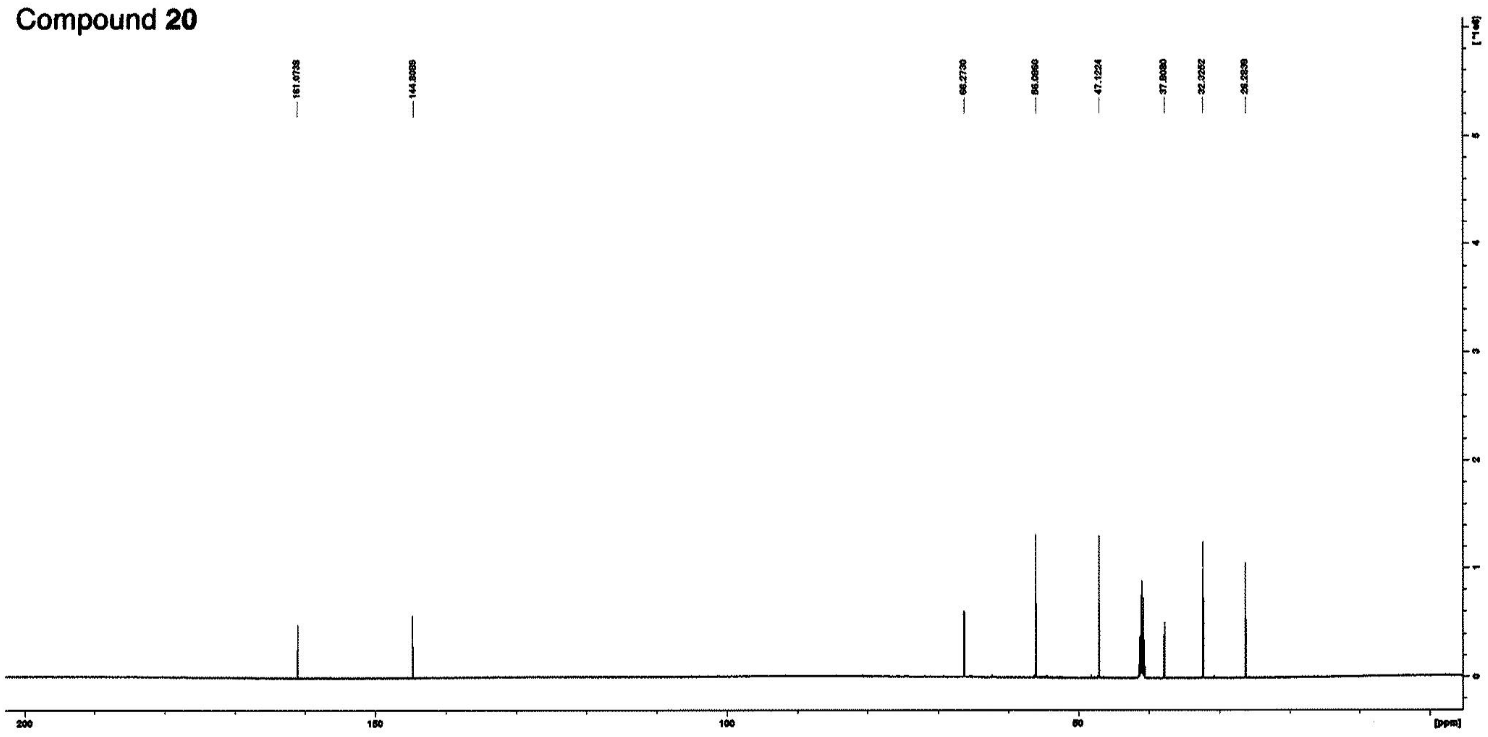

Figure S30. ^{13}C NMR for (*E*)-2-(4-(cyclopentylmethyl)piperazin-1-yl)-2-oxoacetaldehyde oxime.

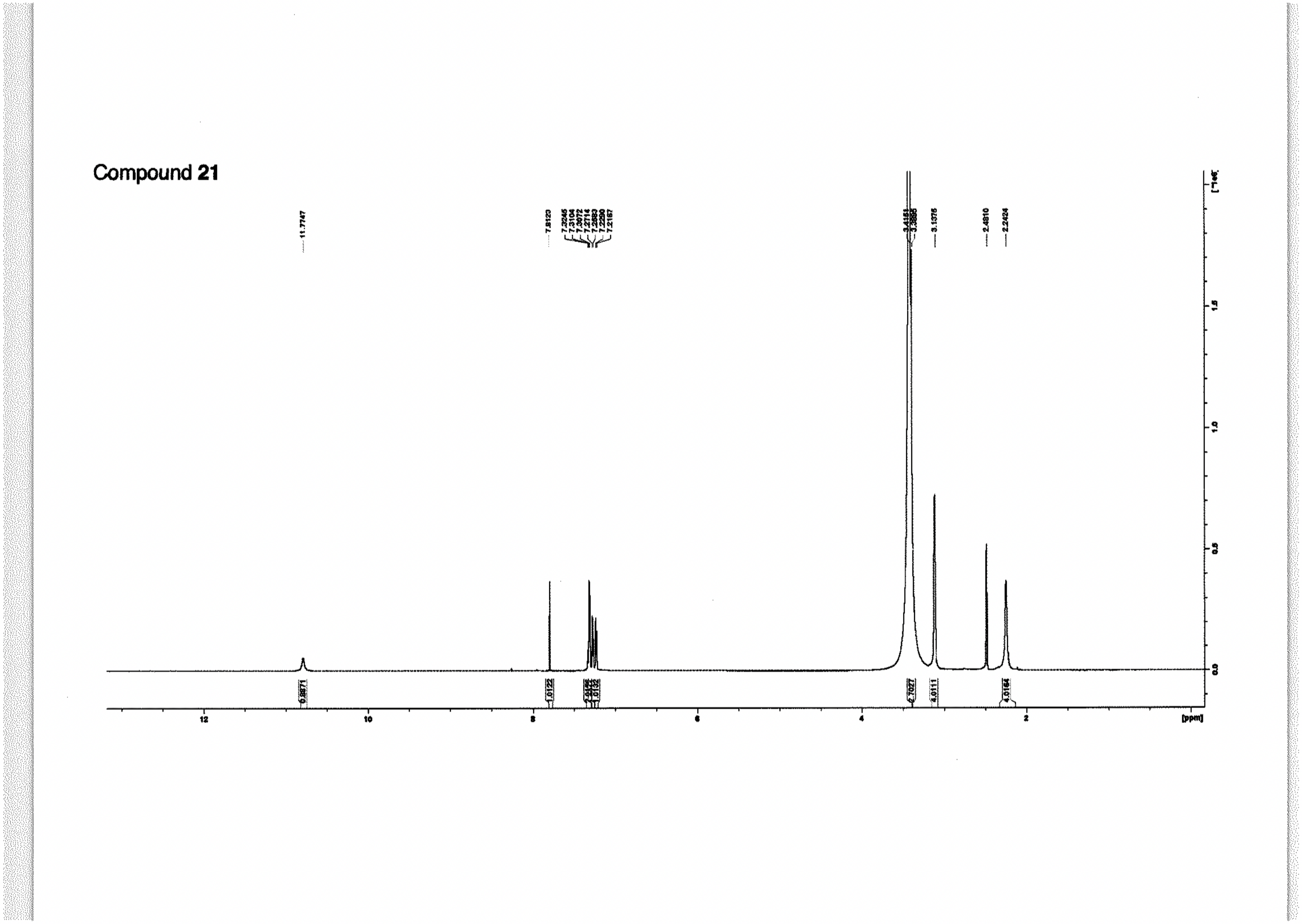

Figure S31. ^{1}H NMR for (*E*)-2-(4-(3-chlorobenzyl)piperazin-1-yl)-2-oxoacetaldehyde oxime.

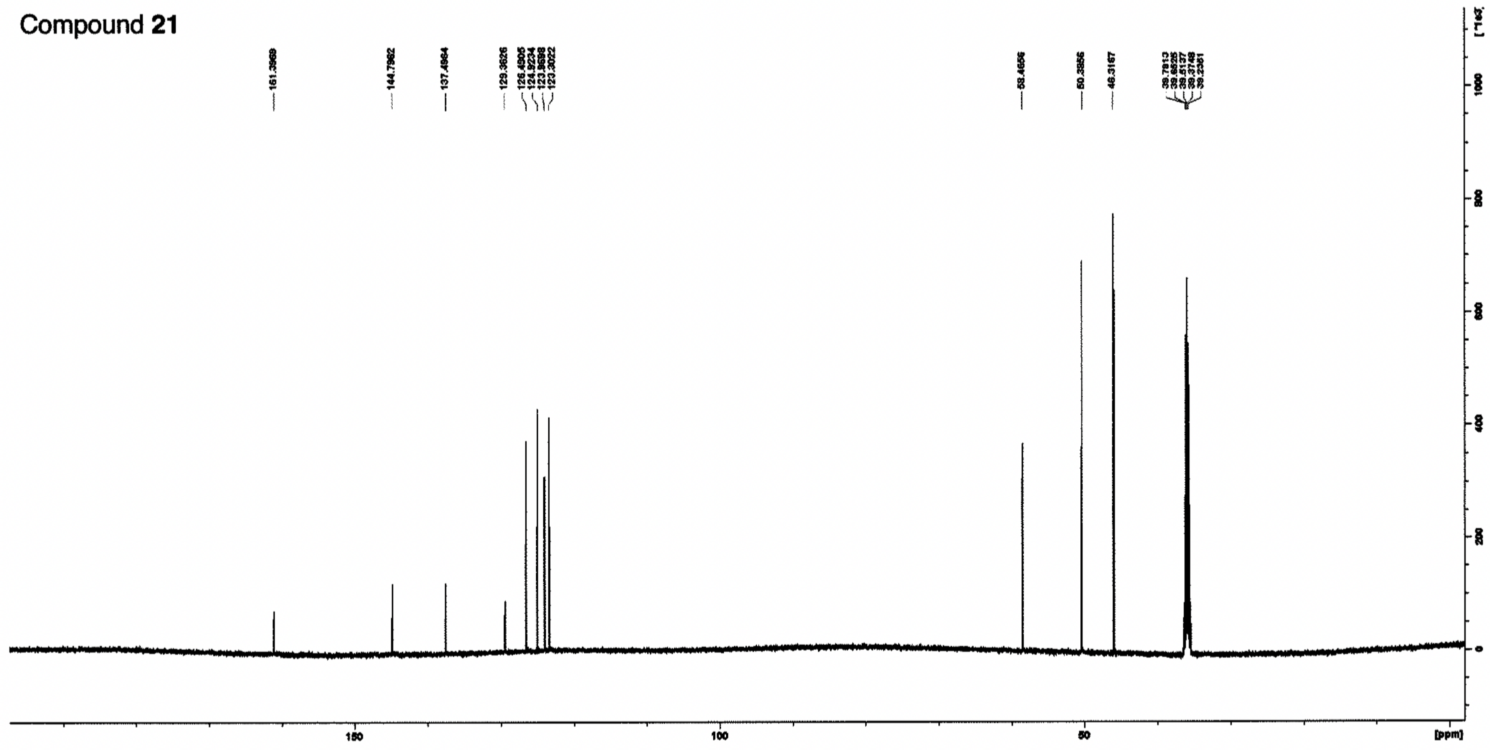

Figure S32. ^{13}C NMR for (*E*)-2-(4-(3-chlorobenzyl)piperazin-1-yl)-2-oxoacetaldehyde oxime.

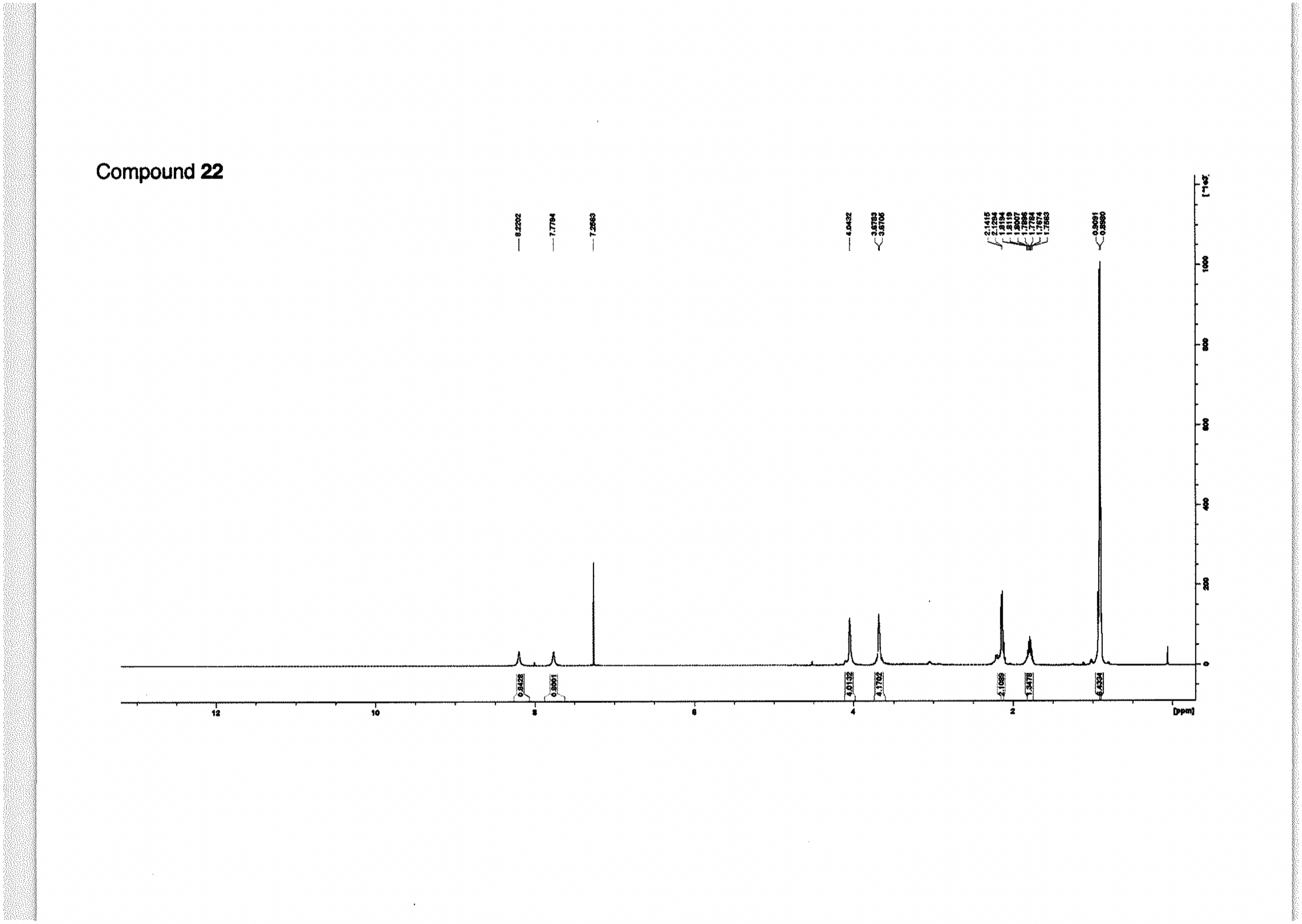

Figure S33. ^{1}H NMR for (*E*)-2-(4-isobutylpiperazin-1-yl)-2-oxoacetaldehyde oxime.

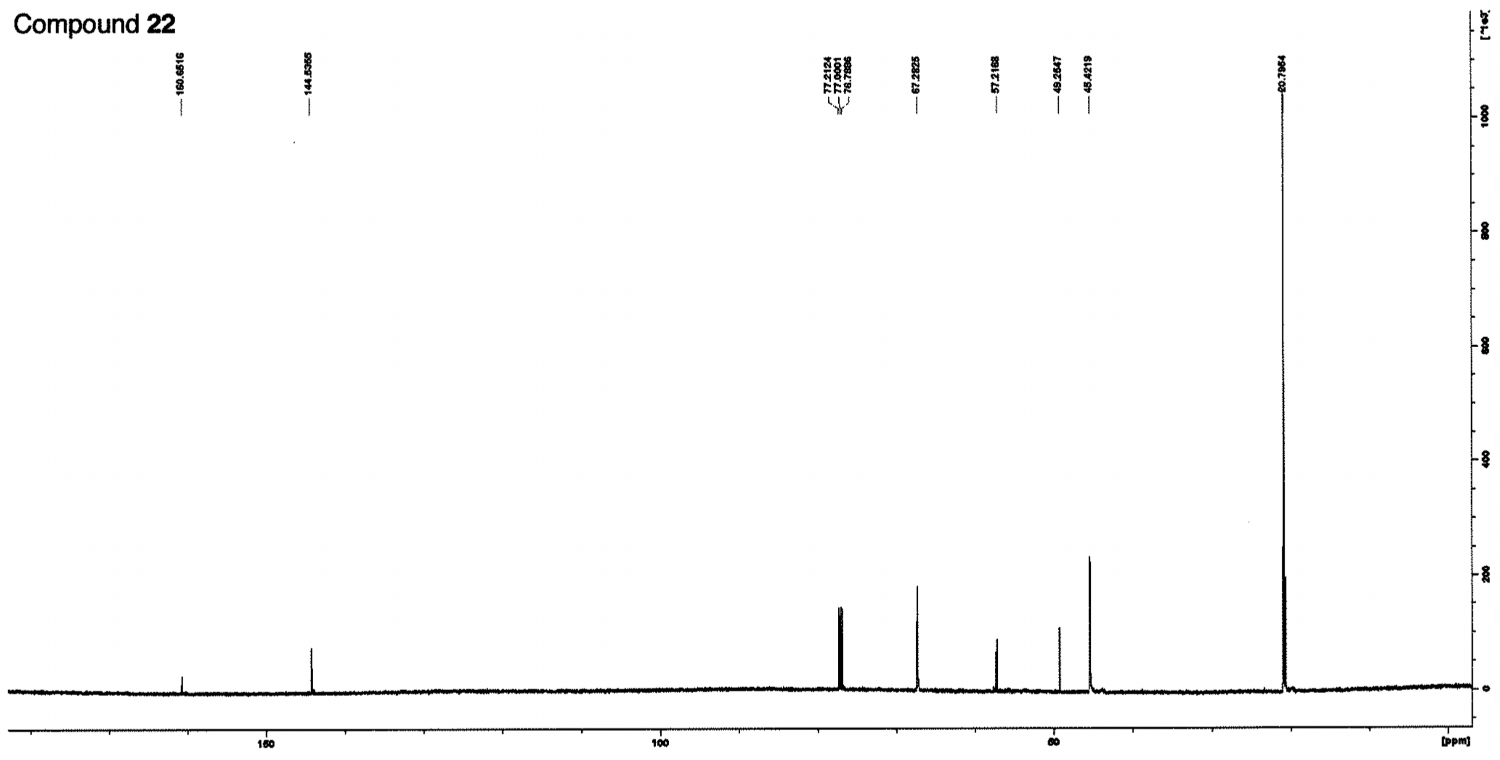

Figure S34. ^{13}C NMR for (*E*)-2-(4-isobutylpiperazin-1-yl)-2-oxoacetaldehyde oxime.

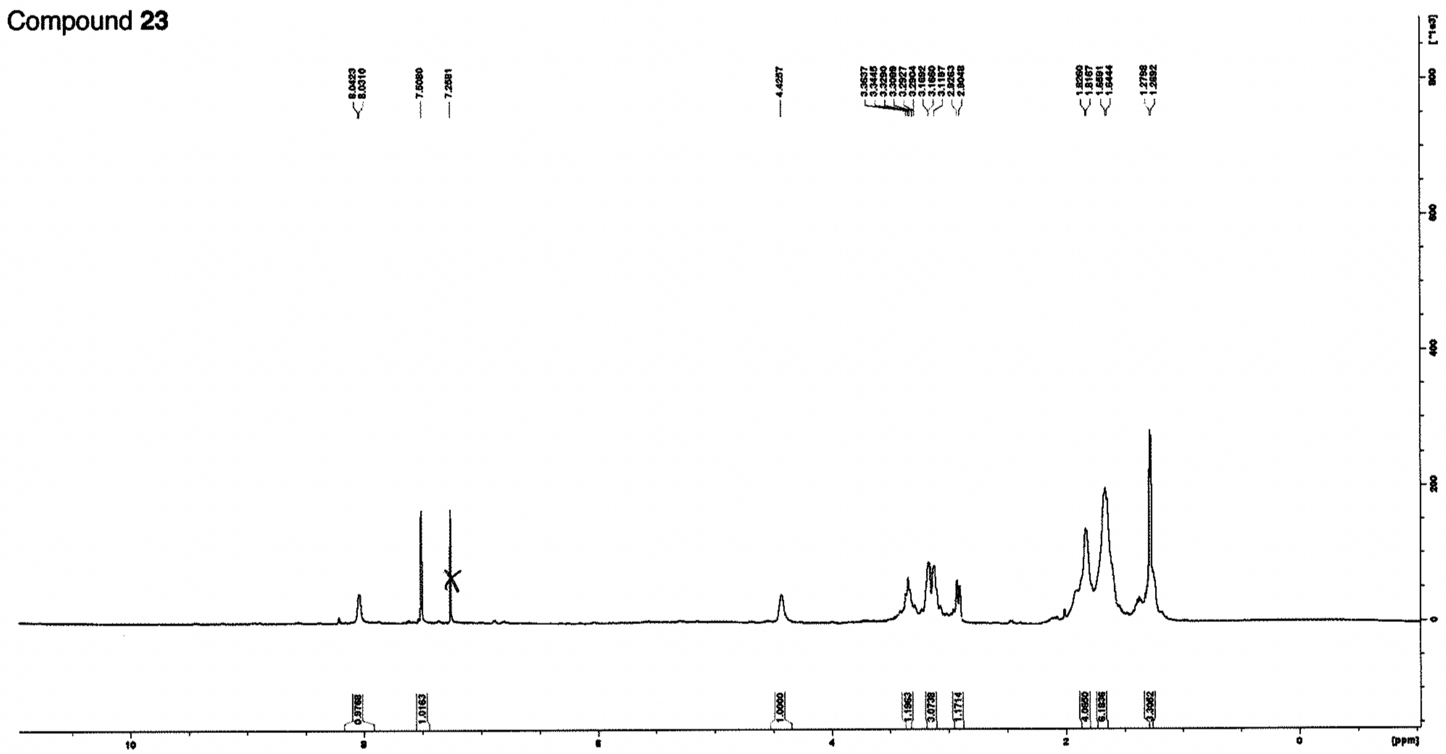

Figure S35. ^{1}H NMR for (*E*)-*N*-(1-(azepan-1-yl)propan-2-yl)-2-(hydroxyimino)acetamide.

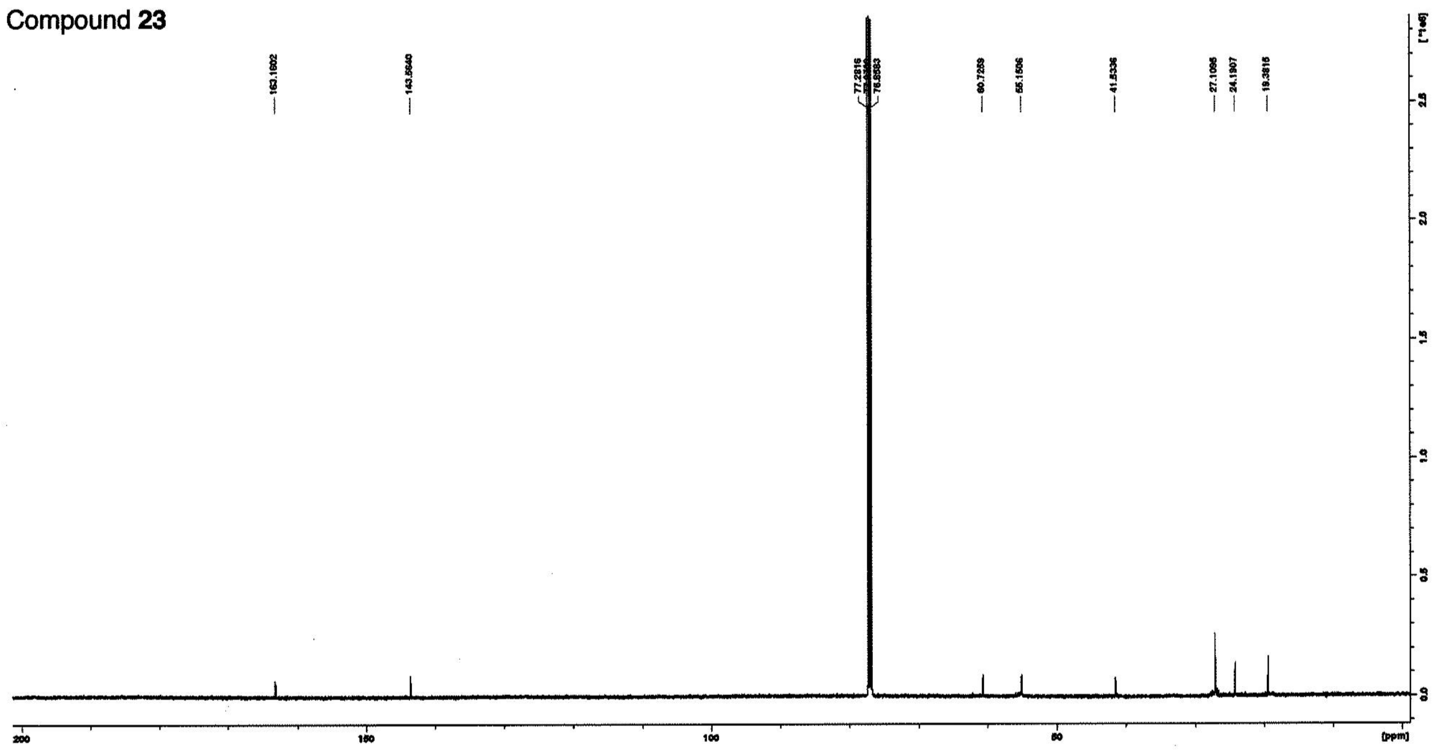

Figure S36. ^{13}C NMR for (*E*)-*N*-(1-(azepan-1-yl)propan-2-yl)-2-(hydroxyimino)acetamide.

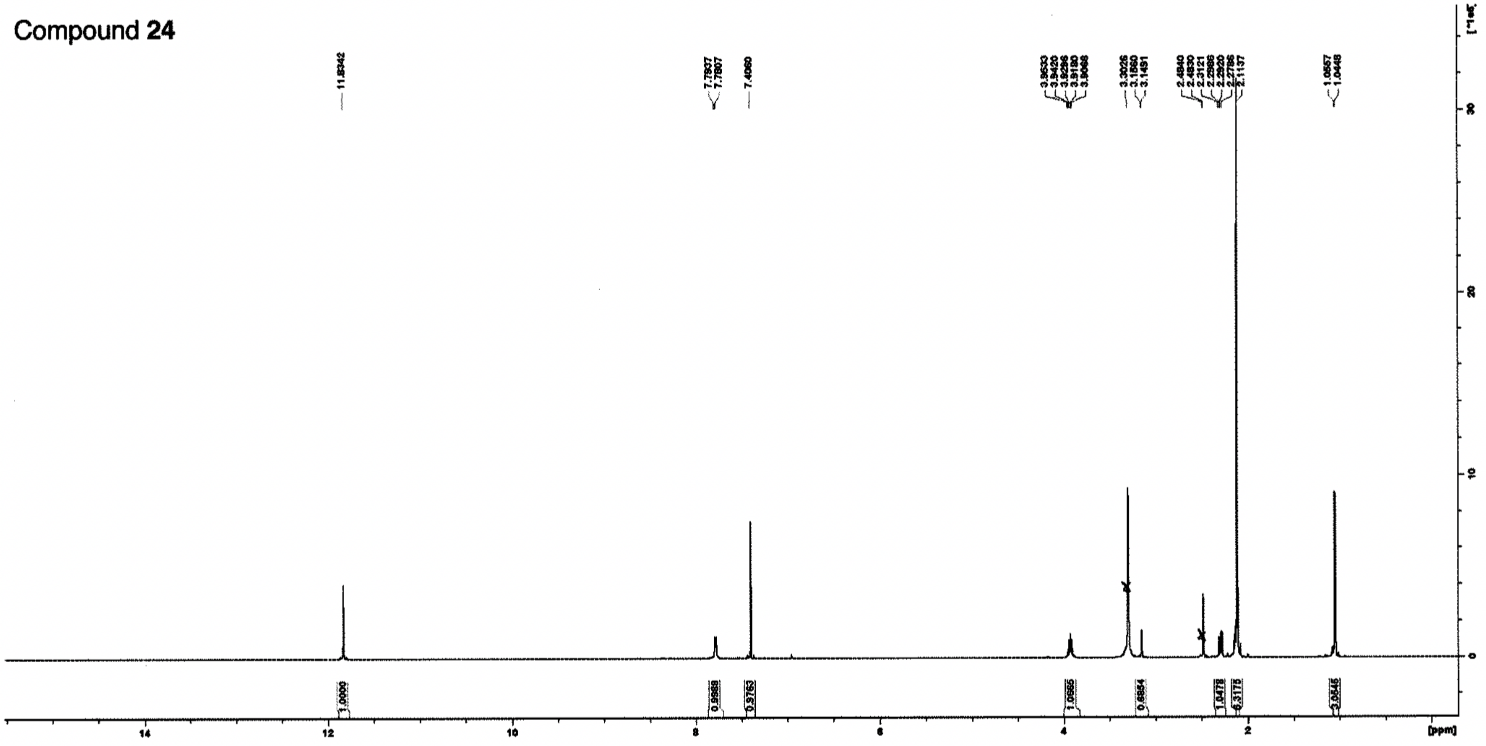

Figure S37. ^{1}H NMR for (*E*)-*N*-(1-(dimethylamino)propan-2-yl)-2-(hydroxyimino)acetamide.

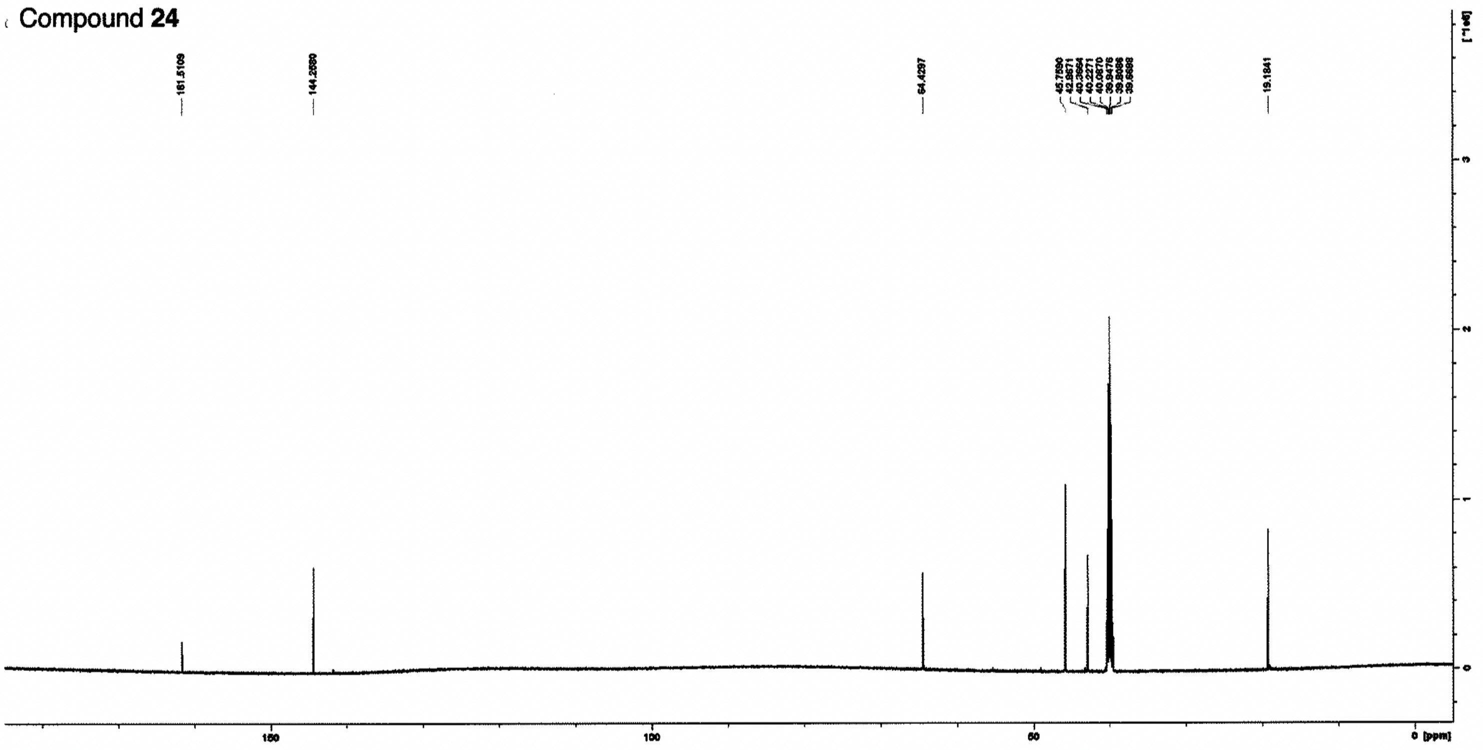

Figure S38. ^{13}C NMR for (*E*)-*N*-(1-(dimethylamino)propan-2-yl)-2-(hydroxyimino)acetamide.

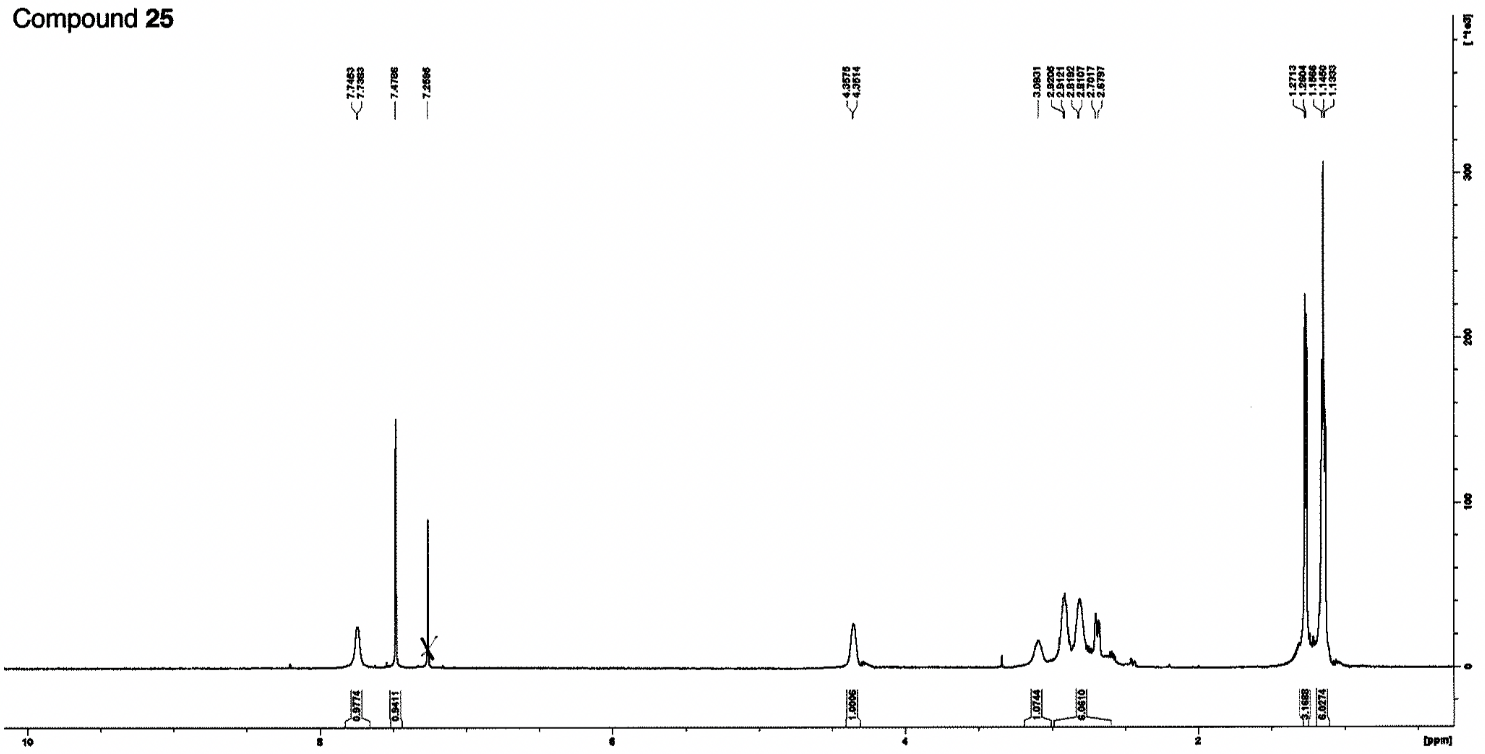

Figure S39. ^{1}H NMR for (*E*)-*N*-(1-(diethylamino)propan-2-yl)-2-(hydroxyimino)acetamide.

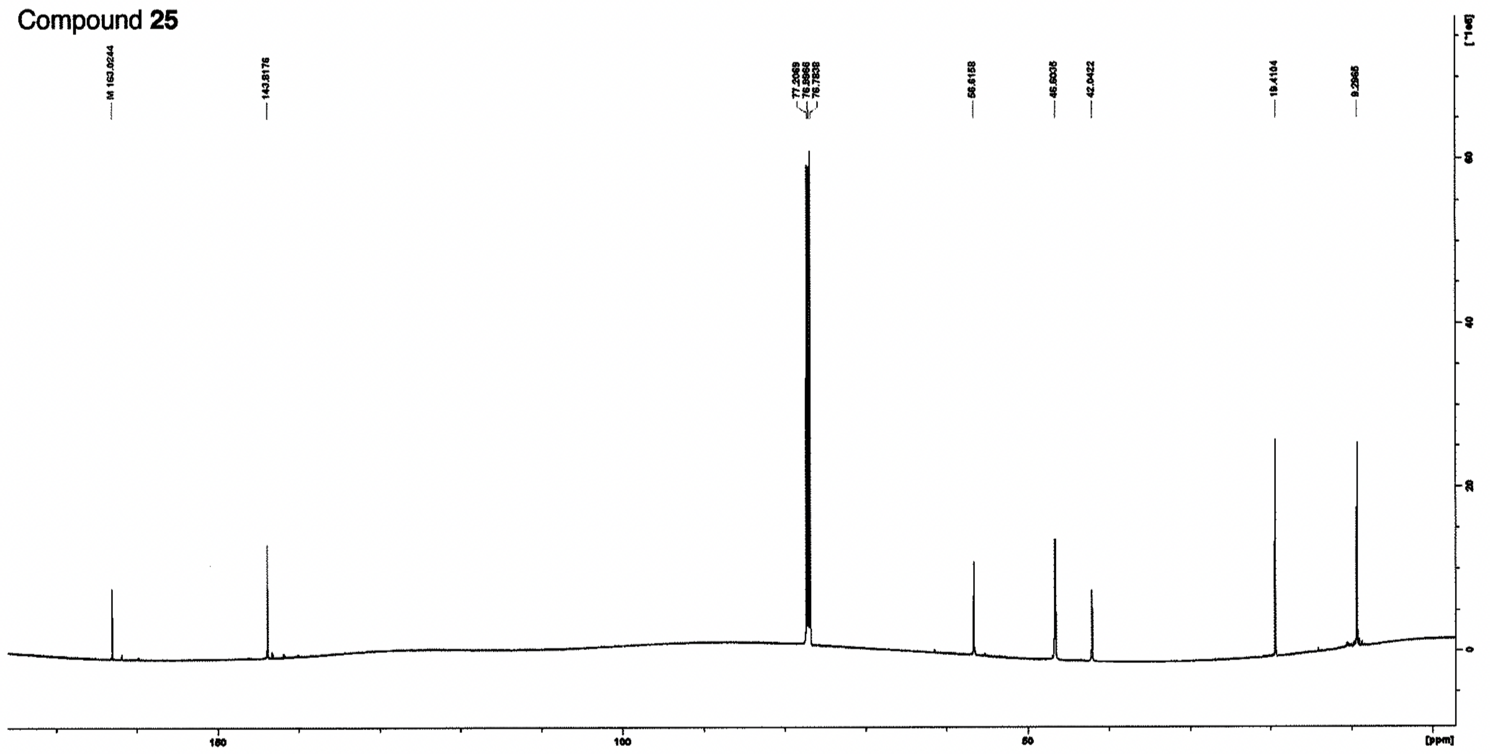

Figure S40. ^{13}C NMR for (*E*)-*N*-(1-(diethylamino)propan-2-yl)-2-(hydroxyimino)acetamide.

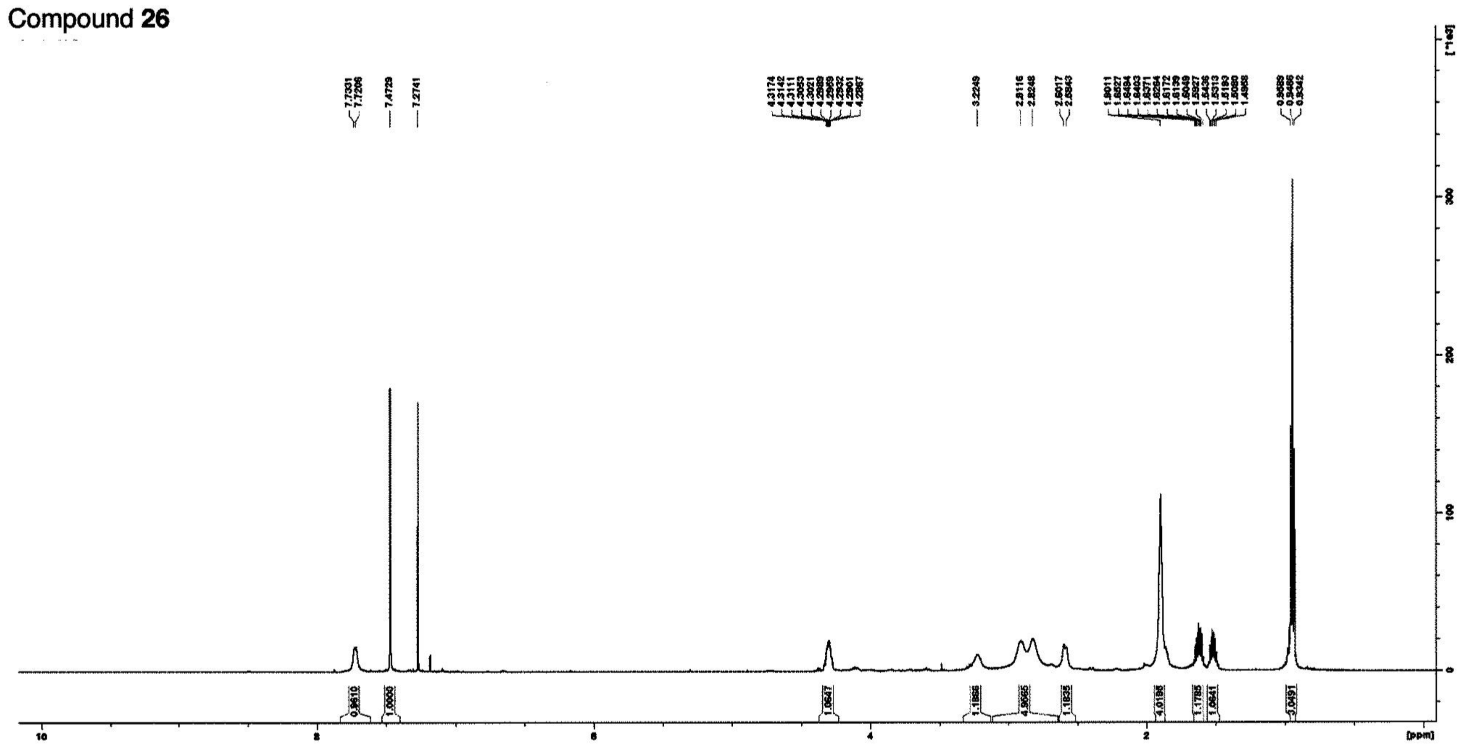

Figure S41. ^{1}H NMR for (*E*)-2-(hydroxyimino)-*N*-(1-(pyrrolidin-1-yl)butan-2-yl)acetamide.

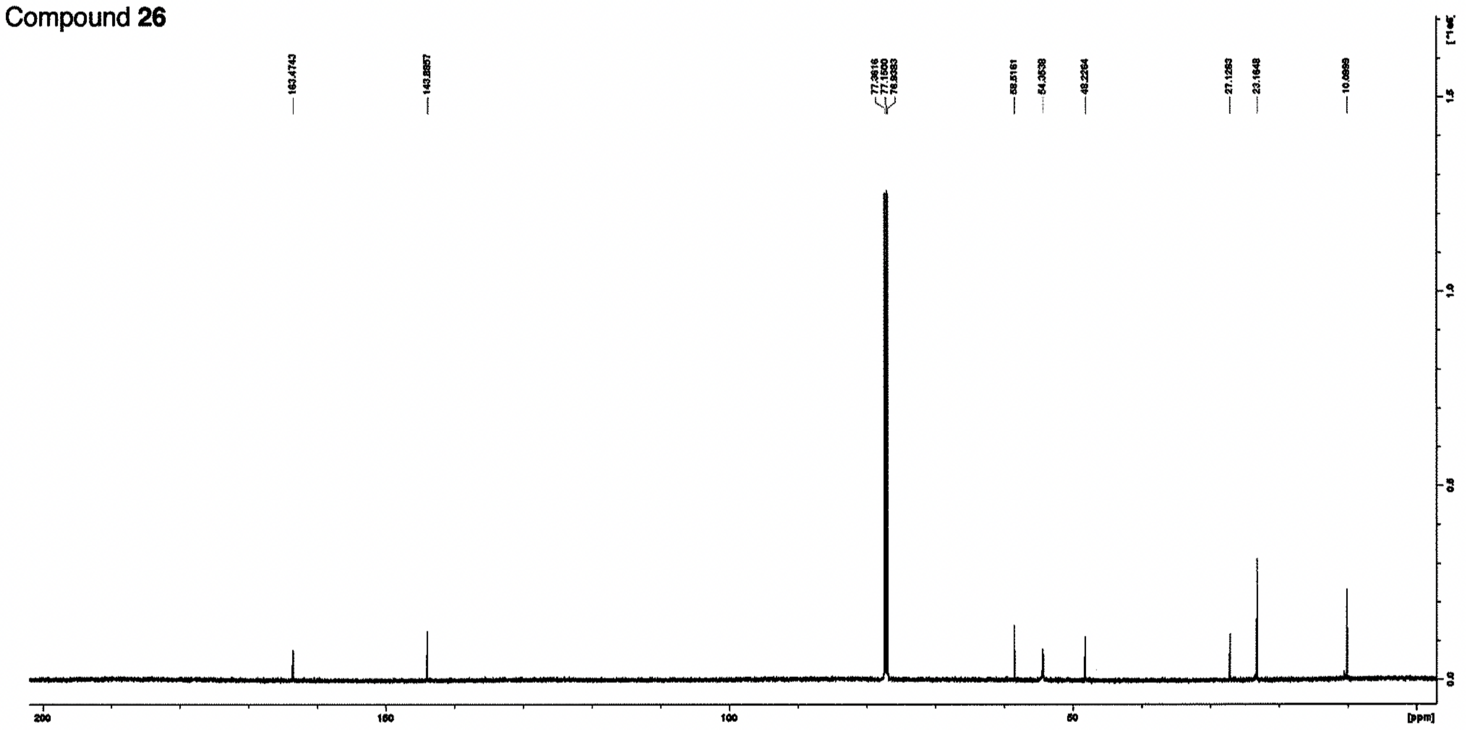

Figure S42. ^{13}C NMR for (*E*)-2-(hydroxyimino)-*N*-(1-(pyrrolidin-1-yl)butan-2-yl)acetamide.

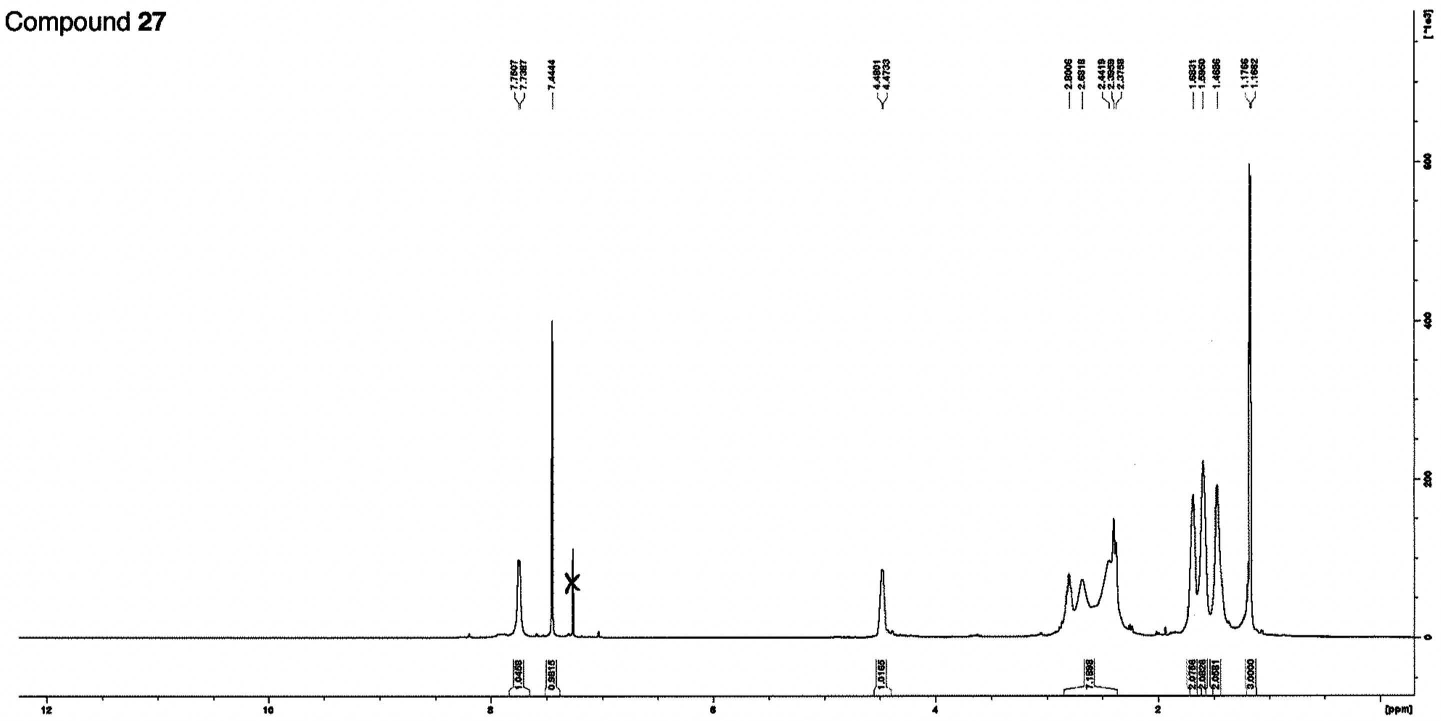

Figure S43. ^{1}H NMR for (*E*)-2-(hydroxyimino)-*N*-(1-(piperidin-1-yl)propan-2-yl)acetamide.

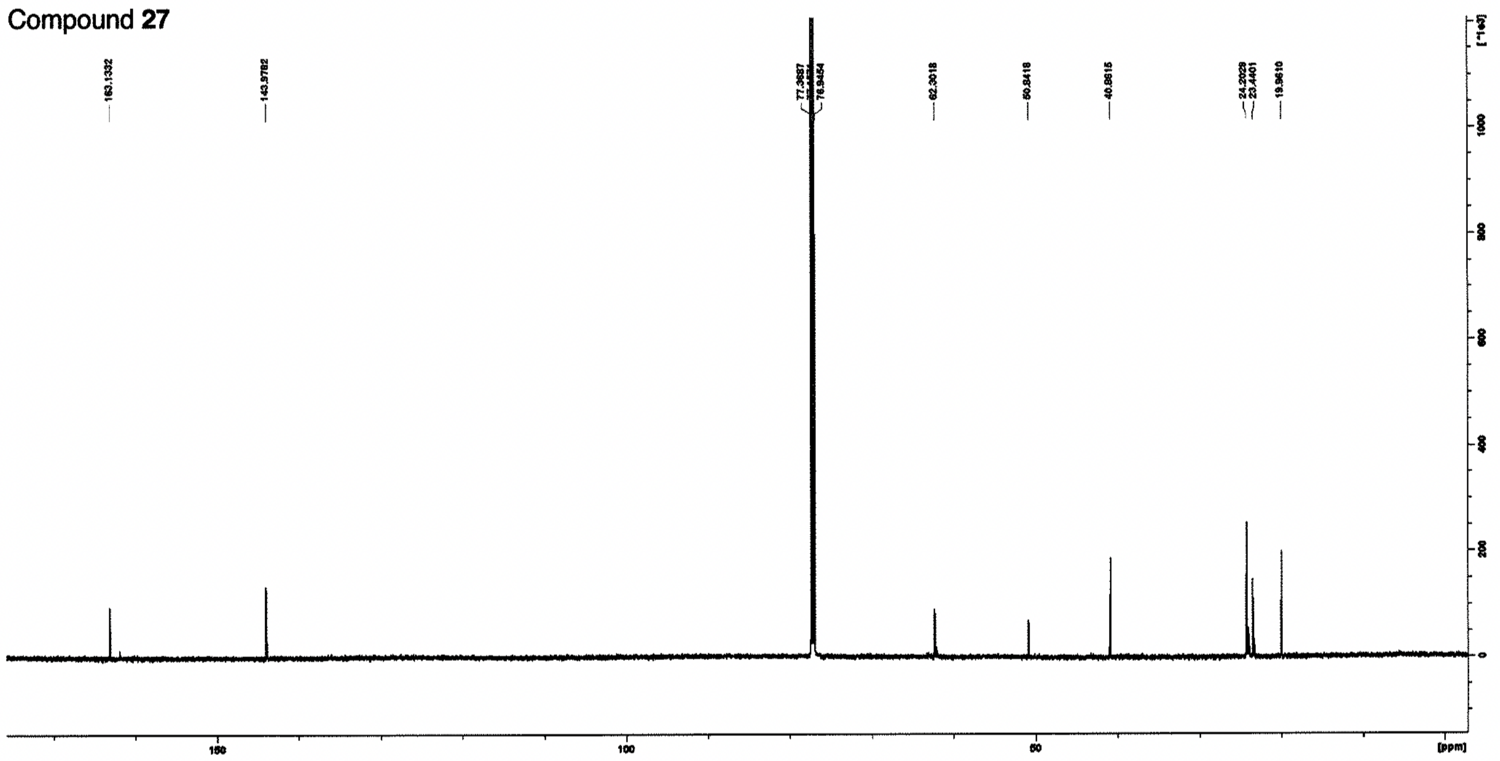

Figure S44. ^{13}C NMR for (*E*)-2-(hydroxyimino)-*N*-(1-(piperidin-1-yl)propan-2-yl)acetamide.

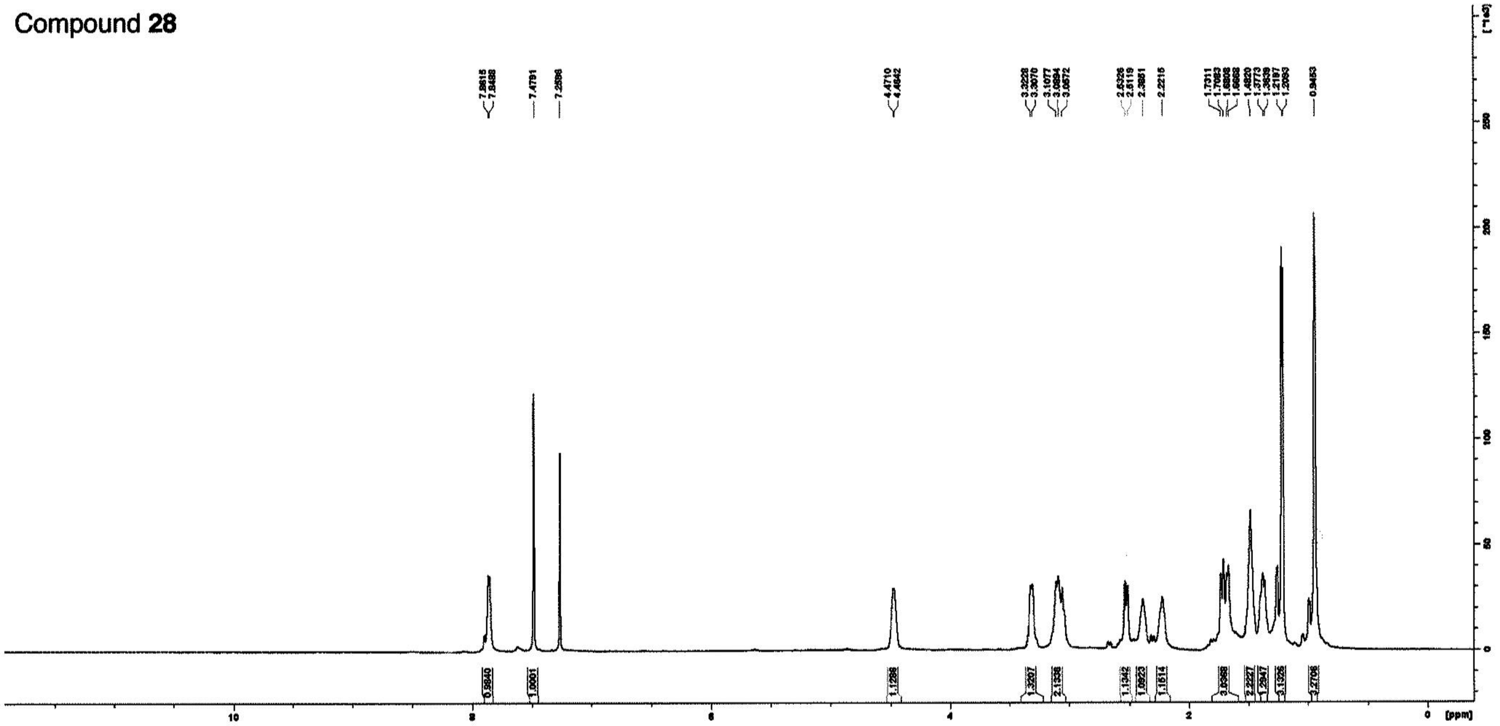

Figure S45. ^{1}H NMR for (*E*)-2-(hydroxyimino)-*N*-(1-(4-methylpiperidin-1-yl)propan-2-yl)acetamide.

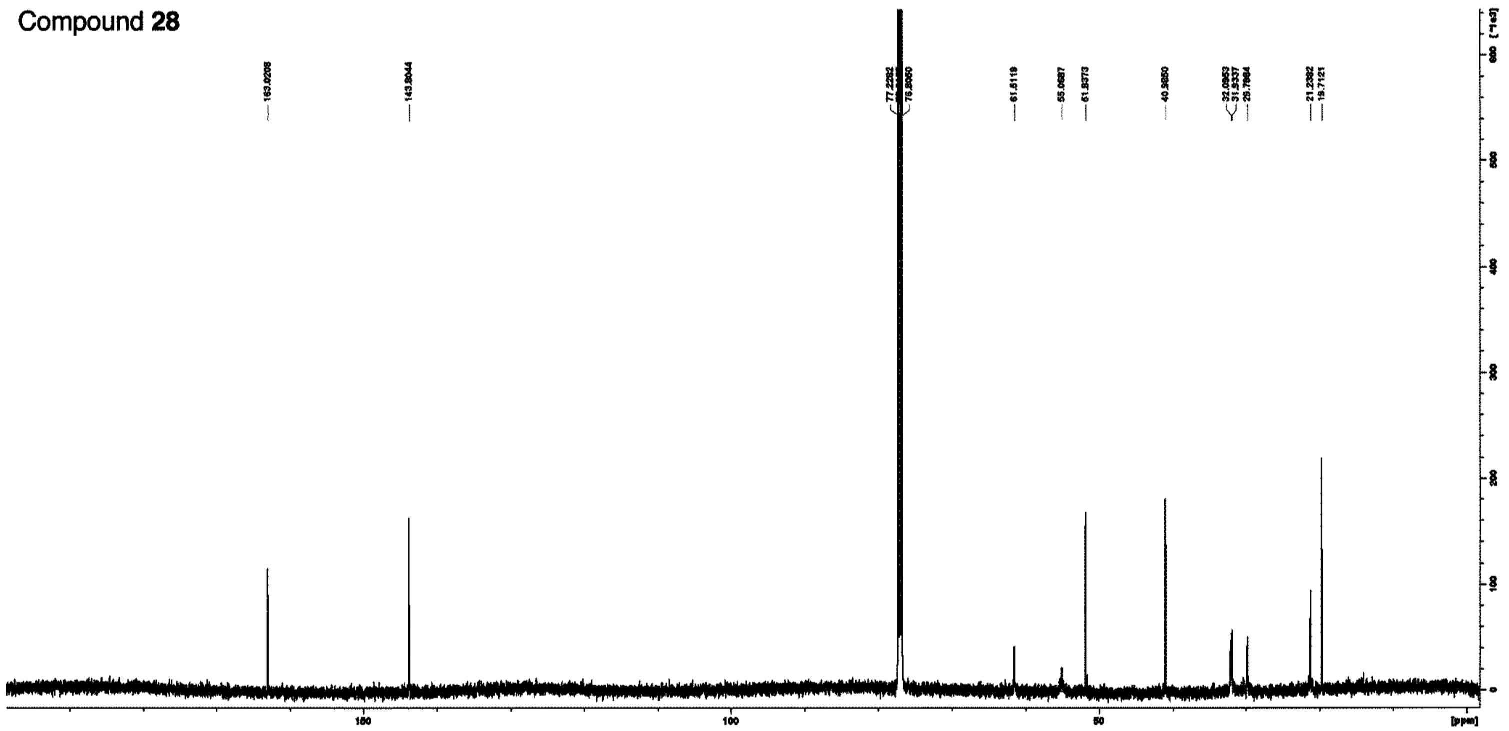

Figure S46. ^{13}C NMR for (*E*)-2-(hydroxyimino)-*N*-(1-(4-methylpiperidin-1-yl)propan-2-yl)acetamide.

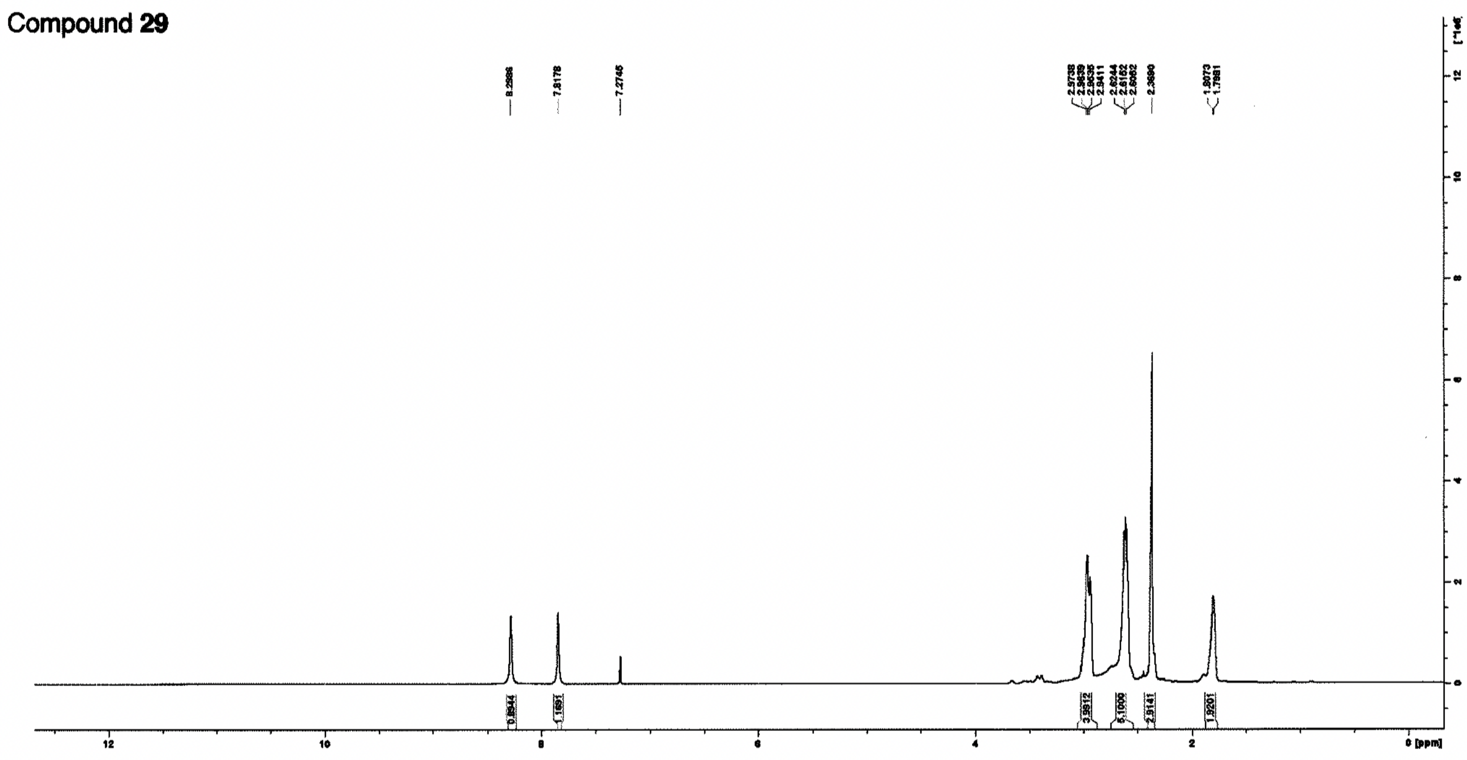

Figure S47. ^{1}H NMR for (*E*)-2-(4-methyl-1,4-diazepan-1-yl)-2-oxoacetaldehyde oxime.

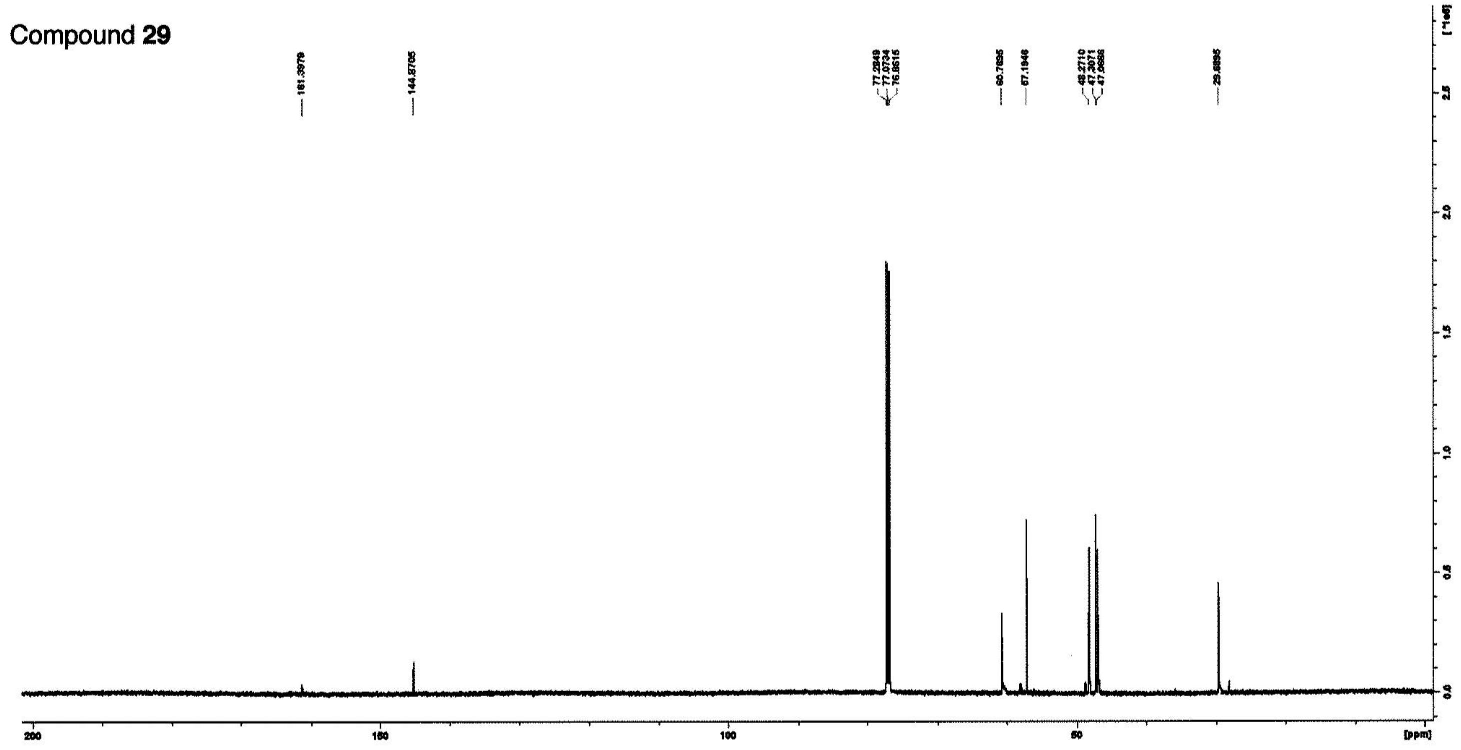

Figure S48. ^{13}C NMR for (*E*)-2-(4-methyl-1,4-diazepan-1-yl)-2-oxoacetaldehyde oxime.

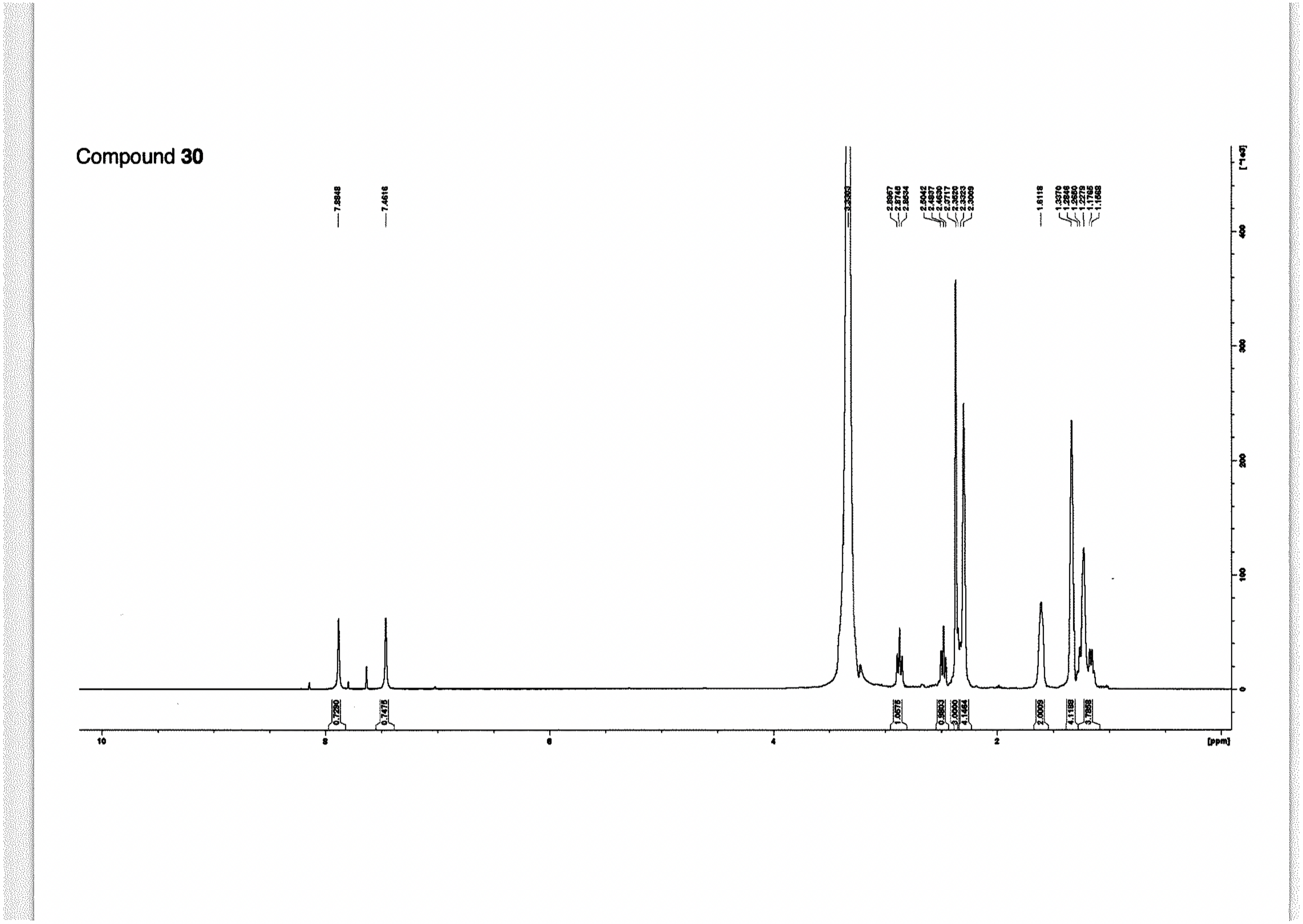

Figure S49. ^{1}H NMR for (*E*)-2-([1,4'-bipiperidin]-1'-yl)-2-oxoacetaldehyde oxime.

Compound **30**

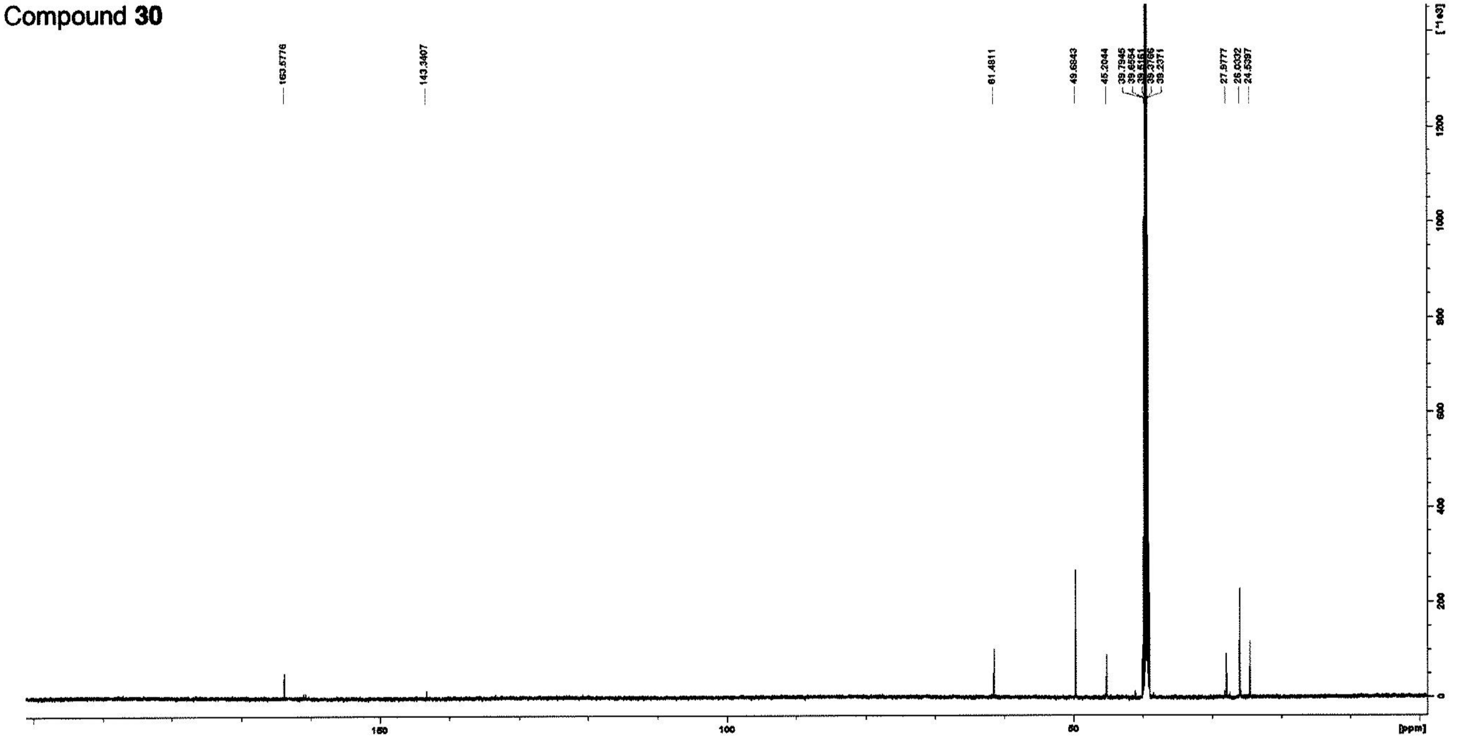

Figure S50. ^{13}C NMR for (*E*)-2-([1,4'-bipiperidin]-1'-yl)-2-oxoacetaldehyde oxime.

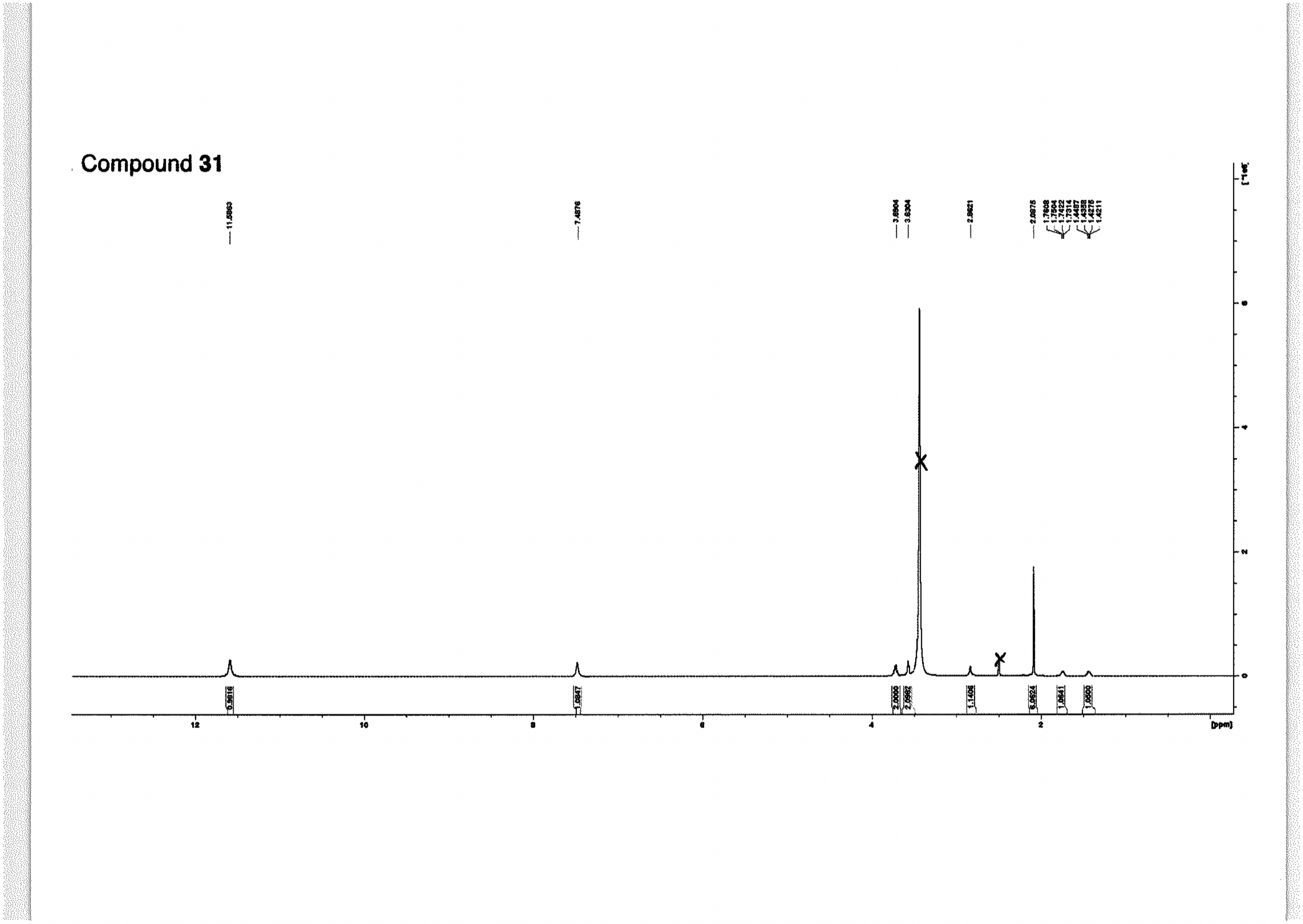

Figure S51. ^{1}H NMR for (*E*)-2-(3-(dimethylamino)pyrrolidin-1-yl)-2-oxoacetaldehyde oxime.

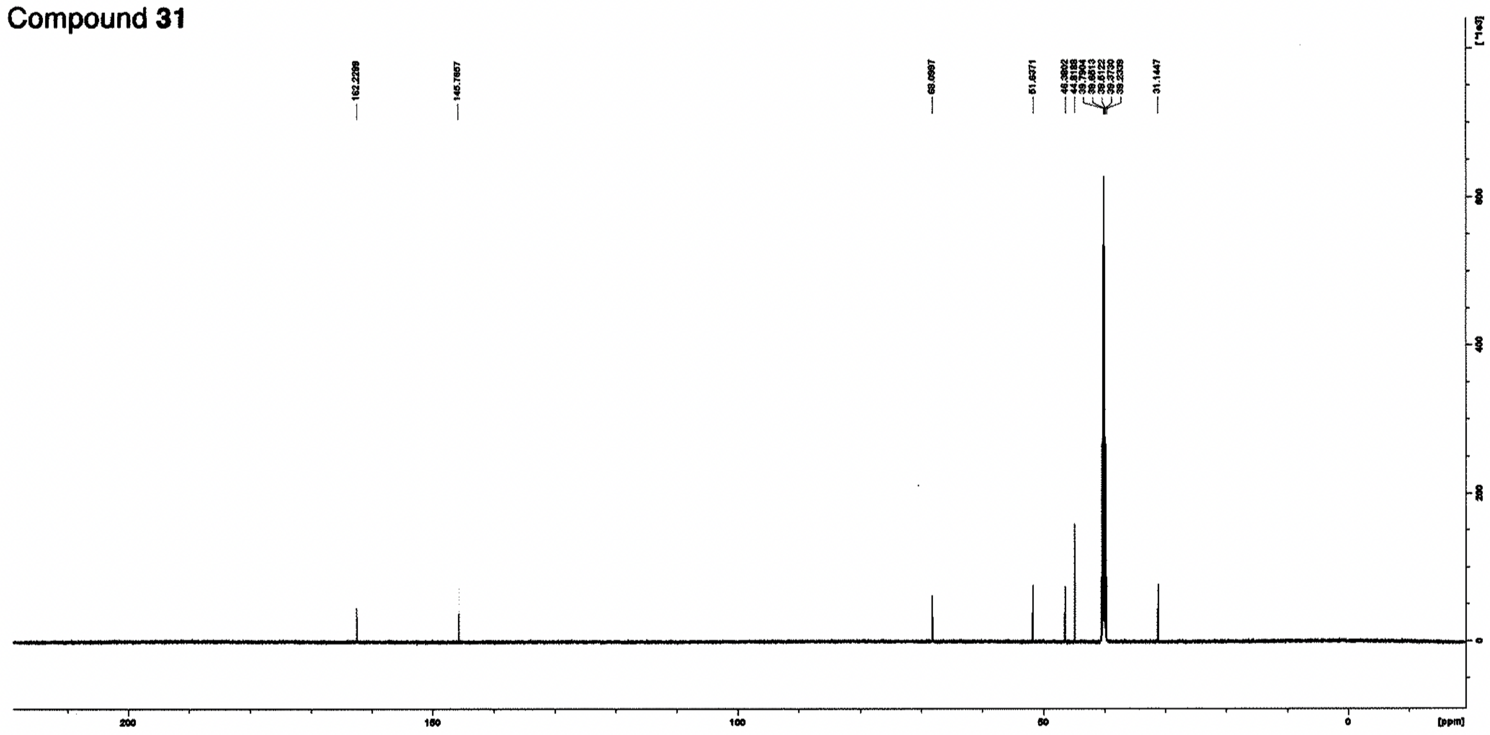

Figure S52. ^{13}C NMR for (*E*)-2-(3-(dimethylamino)pyrrolidin-1-yl)-2-oxoacetaldehyde oxime.

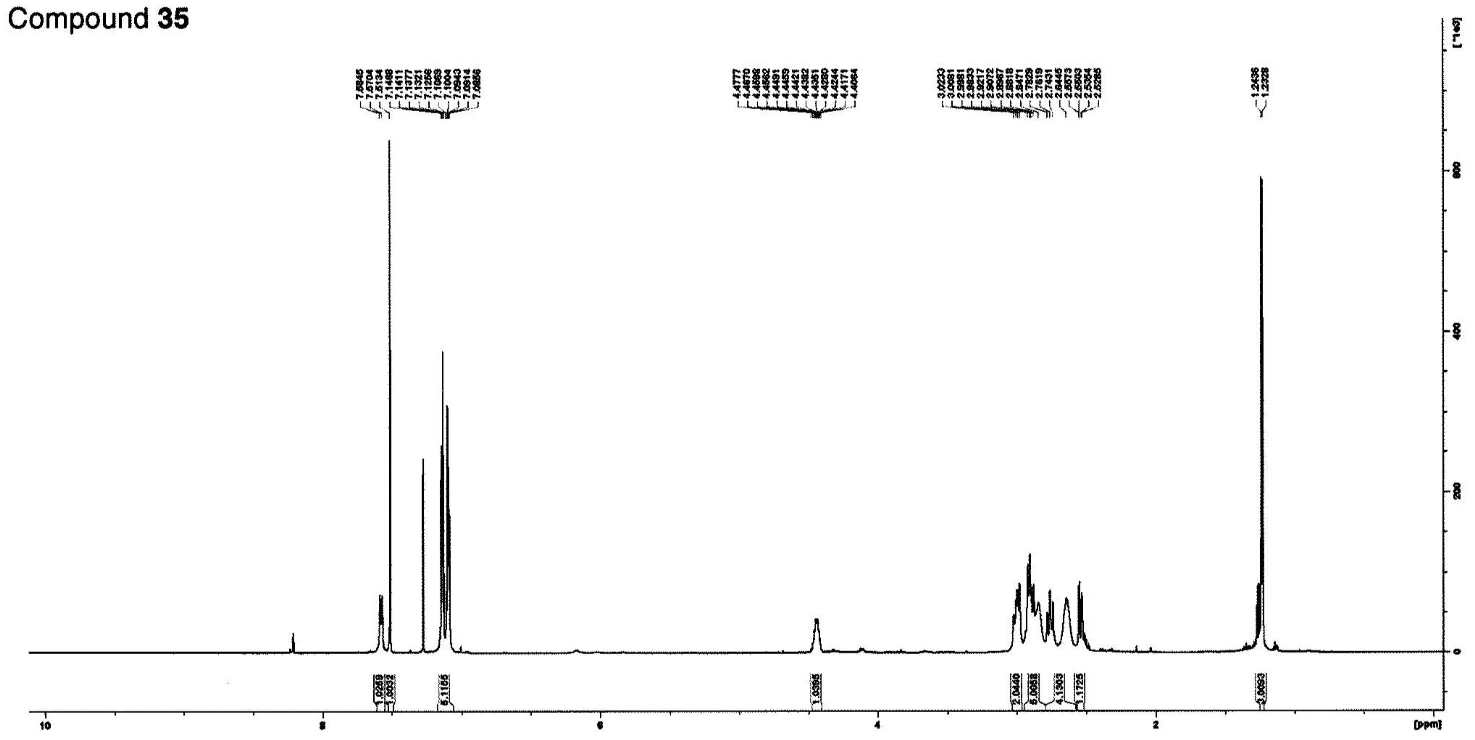

Figure S53. ¹H NMR for (*S*,*E*)-2-(hydroxyimino)-*N*-(1-(1,2,4,5-tetrahydro-3*H*-benzo[*d*]azepin-3-yl)propan-2-yl)acetamide.

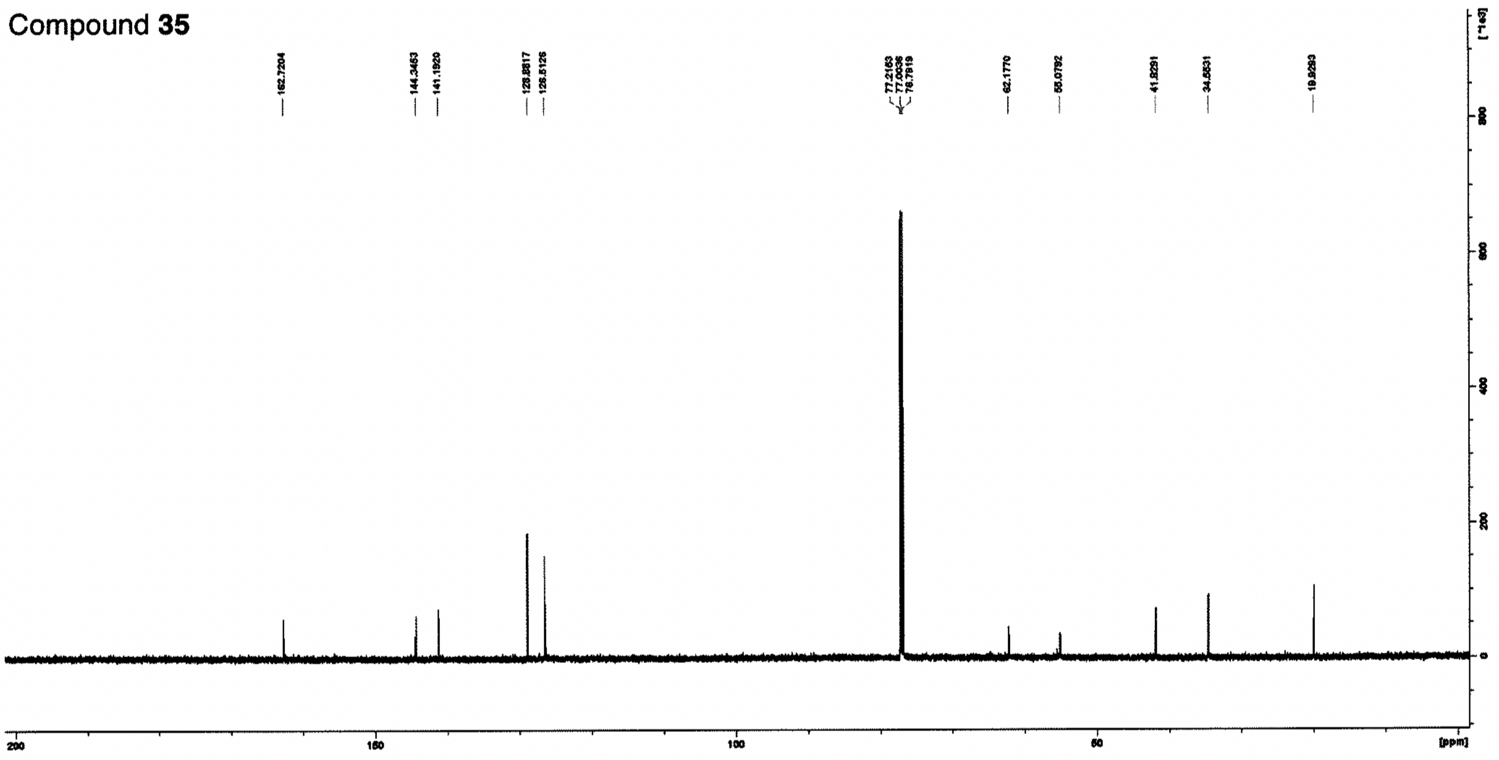

Figure S54. ^{13}C NMR for (*S*,*E*)-2-(hydroxyimino)-*N*-(1-(1,2,4,5-tetrahydro-3*H*-benzo[*d*]azepin-3-yl)propan-2-yl)acetamide.
